# The fusion protein SS18-SSX1 employs core Wnt pathway transcription factors to induce a partial Wnt signature in synovial sarcoma

**DOI:** 10.1038/srep22113

**Published:** 2016-02-24

**Authors:** Luisa Cironi, Tanja Petricevic, Victor Fernandes Vieira, Paolo Provero, Carlo Fusco, Sandrine Cornaz, Giulia Fregni, Igor Letovanec, Michel Aguet, Ivan Stamenkovic

**Affiliations:** 1 Institute of Pathology University of Lausanne and CHUV Lausanne, Switzerland; 2 Center for Translational Genomics and Bioinformatics San Raffaele Scientific Institute Milan, Italy; 3 Swiss Institute for Experimental Cancer Research Ecole Polytechnique Fédérale de Lausanne Lausanne, Switzerland

## Abstract

Expression of the SS18/SYT-SSX fusion protein is believed to underlie the pathogenesis of synovial sarcoma (SS). Recent evidence suggests that deregulation of the Wnt pathway may play an important role in SS but the mechanisms whereby SS18-SSX might affect Wnt signaling remain to be elucidated. Here, we show that SS18/SSX tightly regulates the elevated expression of the key Wnt target *AXIN2* in primary SS. SS18-SSX is shown to interact with TCF/LEF, TLE and HDAC but not β-catenin *in vivo* and to induce Wnt target gene expression by forming a complex containing promoter-bound TCF/LEF and HDAC but lacking β-catenin. Our observations provide a tumor-specific mechanistic basis for Wnt target gene induction in SS that can occur in the absence of Wnt ligand stimulation.

Synovial sarcoma (SS) is an aggressive soft tissue malignancy predominantly of young adults that can develop in virtually any organ and is believed to originate from pluripotent mesenchymal cells^1^,^2^. More than 90% of SS are associated with the chromosomal translocation t(X;18)(p11;q11) that results in fusion of the ubiquitously expressed *SYT/SS18* gene to one of the *SSX* family members, most frequently *SSX1* and 2^3^. Neither SS18 nor SSX proteins have DNA binding domains but both possess protein-protein interaction motifs that mediate association with transcriptional regulators and chromatin remodeling complexes^4^,^5^,^6^.

Mounting evidence suggests that the Wnt pathway is strongly implicated in SS pathogenesis^7^,^8^,^9^. The Wnt family of secreted proteins fulfills key evolutionarily conserved functions in normal development and adult tissue maintenance^10^,^11^,^12^,^13^ and its deregulation by alteration of expression or mutation of its key components including *β*−*catenin, adenomateous polyposis coli (APC)* and *AXIN*, is associated with development and progression of diverse cancer types^13^,^14^,^15^,^16^,^17^,^18^. Expression of SS18/SSX in HEK293 cells has been suggested to activate Wnt- β-catenin signaling. Studies on the MYF5-CRE SS18/SSX2 transgenic model of SS found that SS18-SSX2 aberrantly activates Wnt/β-catenin signaling and that genetic deletion of β-catenin blocks tumor formation^19^. They also suggested that SS18-SSX causes nuclear β-catenin accumulation, possibly by inducing autocrine signaling through its aberrant transcriptional effects. In contrast, introduction of SS18/SSX into NIH3T3 cells induced Wnt ligand-independent accumulation of β-catenin in the nucleus^7^, suggesting an alternative mechanism of SS18-SSX-dependent deregulation of Wnt. Despite elevated expression of several Wnt target genes, particularly *AXIN2*, nuclear localization of ß-catenin is seen in only 30–60% of SS^20^,^21^,^22^ and genetic screens^23^,^24^ revealed low incidence of *β*−*catenin, APC, AXIN1* and *AXIN2* mutations^25^. The molecular mechanisms whereby SS18-SSX may alter Wnt signaling and target gene expression thus remain to be fully elucidated.

Engagement of cell surface receptors by Wnt ligands triggers intracellular signaling that can activate the canonical Wnt/β-catenin and a variety of non-canonical Wnt pathways^12^. A direct consequence of Wnt ligand-receptor interaction is the inactivation of a complex composed of APC, axin and glycogen synthase kinase (GSK3)β, which in the absence of Wnt ligands targets β-catenin for proteosomal degradation^12^. Engagement of receptors by Wnt ligands causes β-catenin to accumulate in the cytoplasm, translocate to the nucleus and bind to the T cell-specific factor/lymphoid enhancer-binding factor (TCF/LEF) complex to modulate TCF/LEF-dependent transcription of target genes. In the absence of β-catenin, TCF/LEF family members form a repressor complex with co-repressor partners, primarily transducin-like enhancer of split 1 (TLE1)^26^ and histone deacetylase (HDAC)^27^,^28^. Induction of Wnt target gene expression is therefore proposed to occur in at least two steps^27^: nuclear β-catenin first engages in promoter de-repression by disrupting the repressor complex composed of HDAC, TLE and TCF/LEF; as its level continues to rise in response to Wnt ligands, β-catenin associates with TCF/LEF1 to induce transcriptional activation. TCF/LEF-1 may therefore transition from transcriptional repressors when bound to TLE and HDAC to transcriptional activators when dissociated from TLE/HDAC and bound to β-catenin^27^.

In the present work we addressed possible functionally relevant interactions between SS18-SSX and nuclear components of the Wnt signaling pathway in mouse C3H10T1/2 pluripotent mesenchymal cells. We show that in the absence of exogenous soluble Wnt ligands, SS18-SSX1 forms complexes with TCF/LEF, TLE1 and HDAC that exclude β-catenin and induces a partial Wnt signature that includes elevated expression of *AXIN2* in addition to several other Wnt target genes. Because *AXIN2* faithfully reflects Wnt pathway activation in neither tissue nor context-dependent manner and is among the most strongly upregulated Wnt target genes in SS^9^,^29^,^30^, we interrogated the functional relationship between SS18-SSX, HDAC and TCF/LEF at the *AXIN2* promoter. Our observations suggest that SS18-SSX participates in de-repression of *AXIN2* expression by inhibiting HDAC and augmenting Histone H3K9 acetylation at its promoter and propose a mechanistic basis for SS18-SSX-mediated deregulation of Wnt target gene expression in permissive cells.

## Results

### *AXIN2* transcripts in primary SS correlate with SS18-SSX expression levels

**Figure 1:**
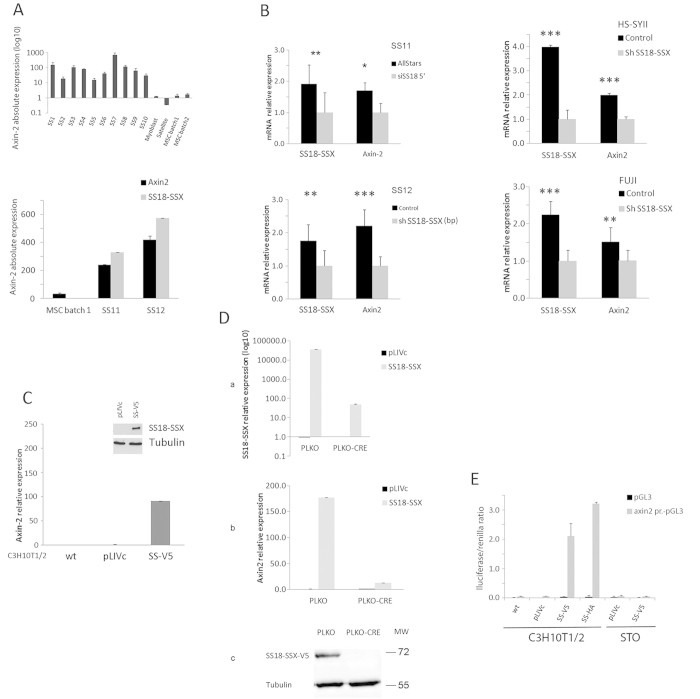
SS18-SSX regulates mRNA levels in cell context-specific manner. (**A**) Q -PCR analysis of *AXIN2* in primary human SS, satellite cells, myoblasts and MSC. (**B**) Q-PCR analysis of *AXIN2* and *SS18-SSX* (indicated) after SS18-SSX depletion in SS cells and SS cell lines. (**C,D**) Q-PCR analysis of *AXIN2* transcripts and *SS18-SSX* (indicated) in C3H10T1/2 cells. In (**C**) cells were infected with V5-tagged SS18-SSX1 (SS-V5) or empty pLIVc vector and selected for 10 days in 1 μg/ml puromycin. In (**D**) selected C3H10T1/2^*pLIVc*^ and C3H10T1/*2*^*SS18-SSX1-V5*^ cells were infected with LV-CRE pLKO.1 or an empty pLKO.1 vector and harvested 96 hours later. Protein expression and knockdown were assessed by Western blot analysis (inset in (**C**) and lowest panel in **D**) using mouse anti-V5 and HRP-conjugated goat anti-mouse IgG. Monoclonal mouse anti-tubulin antibody provided the loading control. Q-PCR results are representative of two (**B**) or three (**D**) independent experiments. Bar represents the SD of triplicate PCRs; in B the significance (indicated by asterisks) of *SS18-SSX* and *AXIN2* repression was p = 0.014 and p = 0.022, respectively, for SS11, p = 0.0130 and p = 1.03E-5 for SS12, p = 0.000738 and 6.74E-5 for HS-SYII and p = 0.000297 and 0.0103 for FUJI. (**E**) The effect of SS18-SSX1 expression on mouse *AXIN2* promoter activity was measured in STO cells stably expressing V5-tagged SS18-SSX1 protein or empty pLIVc, and in wild type C3H10T1/2, C3H10T1/2^*pLIVc*^, C3H10T1/*2*^*SS18-SSX1-V5*^ and C3H10T1/*2*^*SS18-SSX1-HA*^ cells. The ratio of *firefly* to *renilla* luciferase activity is reported. Results are representative of three independent experiments. Error bars represent the SD of triplicate tests.

Analysis, by real time PCR, of *AXIN2* expression in 10 synovial sarcoma specimens revealed one to three orders of magnitude higher levels of *AXIN2* mRNA than in populations of freshly isolated primary candidate cells of origin of SS, including human mesenchymal stem cells (MSCs), myoblasts and satellite cells (Fig. 1A upper panel). The same analysis of two additional freshly isolated SS (SS11 from the lung and SS12 from the lower leg) yielded comparable results (Fig. 1A, lower panel). To verify that elevated *AXIN2* message in SS is a direct consequence of SS18-SSX expression, we depleted of the fusion protein two SS cell lines (HS-SYII and FUJI) and cells from the two fresh surgical SS samples. We used either a pool of siRNAs directed against the 5′region of human SS18/SYT or a pool of two shRNAs in the lentiviral pLVShRNAmir plasmid targeting the SS18-SSX breakpoint (ShSS18-SSXbp) or an ShRNA already described by Kadoch and Crabtree^31^. AllStars siRNA, empty pLVShRNAmir vector or empty pLKO.1 vector provided negative controls. As assessed by qPCR, *SS18-SSX1* and *AXIN2* mRNA were depleted respectively by 48% and 40% in SS11, 43% and 54% in SS12, 75% and 50% in HS-SYII, 55% and 34% in FUJI cells (Fig. 1B), consistent with tight association between *AXIN2* transcripts and SS18-SSX expression in SS.

### SS18-SSX1 regulates *AXIN2* promoter activity in cell context specific manner

To address the mechanism whereby SS18-SSX deregulates Wnt signaling in primary cells, we sought to identify mesenchymal cells that are permissive for robust SS18-SSX-mediated induction of *AXIN2* expression. We therefore introduced HA- or V5-tagged SS18-SSX1 into a range of cell types, including primary human MSC from both bone marrow and peripheral tissues, freshly isolated human myoblasts and satellite cells, primary bone marrow-derived mouse mesenchymal progenitor cells (MPC), and pluripotent mouse mesenchymal C3H10T1/2 cells that display MSC type plasticity^32^,^33^ (Fig. 1C). Comparable expression of SS18-SSX1, as assessed by Western blot analysis, was achieved in all of the selected cell types (not shown) but only C3H10T1/2^SS18-SSX1^ cells displayed roughly 100 fold induction of *AXIN2* that mimicked the expression observed in primary SS samples (Fig. 1A,C). The effect did not differ significantly whether SS18-SSX was tagged with C-terminal V5 or N-terminal HA (data not shown) and required expression of the entire fusion protein as neither wt nor truncated SS18/SYT over-expression induced *AXIN2* expression (Fig. S1).

Further evidence that SS18-SSX regulates *AXIN2* transcript levels was provided by removing the provirus genome (flanked by two LOXp sites) through the expression of the CRE recombinase. Q-PCR analysis of SS18-SSX and *AXIN2* revealed a very strong reduction of both transcripts in C3H10T1/*2*^*SS18-SSX1-V5*^ cells expressing the CRE recombinase (Fig. 1D,a,b) and SS18-SSX protein was reduced to a level below the detection of Western blot (Fig. 1D,c).

To determine whether SS18-SSX1 affects *AXIN2* promoter activity, an *AXIN2* promoter-driven luciferase reporter (from –2954 to +2688) (Gene ID: 12006, 1^st^ ATG = +1) was constructed in the PGL3 vector. C3H10T1/2^*pLIVc*^, C3H10T1/*2*^*SS18-SSX1-V5*^ and C3H10T1/*2*^*SS18-SSX1-HA*^ cells were transiently transfected with either the reporter or an empty PGL3 plasmid. STO mouse fibroblasts (where induction of *AXIN2* following expression of SS18-SSX1 was not observed) transfected with the same constructs provided a negative control. Luciferase activity was measured 48 hours after transfection and normalized to that of *renilla* as an internal control. C3H10T1/2^*wt*^ and C3H10T1/2^*pLIVc*^ cells as well as STO^*pLIVc*^ and STO^*SS18-SSX-V5*^ containing the reporter plasmid displayed no significant increase in luciferase activity compared to their PGL3 containing counterparts. By contrast, robust induction of luciferase activity was observed in both C3H10T1/*2*^*SS18-SSX1-V5*^ and C3H10T1/2^*SS18-SSX1-HA*^ cells (Fig. 1E).

### SS18-SSX expression induces a partial Wnt signature in C3H10T1/2 cells

We next compared transcriptome changes in C3H10T1/*2*^*SS18-SSX1-V5*^ and C3H10T1/2^*pLIVc*^ cells on Affymetrix arrays and analyzed >2 fold differentially expressed probesets (Tables S1 and S2). Comparison of lists of both repressed and induced genes in C3H10T1/2^*SS18-SSX1*^ cells to existing databases revealed a significant overlap with SS signatures reported by Francis *et al.*^30^ (p = 10^−6^ for repressed and 10^−10^ for induced genes) and Baird *et al.*^34^ (p = 10^−6^ for induced genes). Thus, SS18/SSX1 induces a SS-related transcriptome in C3H10T1/2 cells (Table S3).

Over-representation of a limited selection of KEGG pathway terms for induced genes (complete lists are found on the GEO website) in C3H10T1/2^*SS18-SSX1*^ cells included Wnt signaling pathway (KEGG id 04310, p = 0.0011). Particularly significant was over-representation of Wnt signaling-associated and Wnt target genes among transcripts induced by SS18-SSX1 in C3H10T1/2 cells (p ~ 10^−13^, Table S4). To compare SS18-SSX- and Wnt ligand-induced gene signatures in C3H10T1/2, we analyzed transcriptome changes in C3H10T1/2 cells subjected to 0.1 μM recombinant Wnt3a stimulation for 24 hrs. Using three biological replicates at a 10% FDR, we identified 127 up- and 148 down-regulated probes. A highly significant overlap was found with genes differentially expressed upon introduction of SS18-SSX with p values of 3.54E-22 and 1.77E-9 for induced and repressed genes, respectively (Table S4 and S5) indicating that SS18-SSX induces a partial Wnt signature in C3H10T1/2 cells. Wnt target genes were not found among SS18-SSX1-induced or repressed transcripts in STO cells, underscoring context specificity of the SS18-SSX-induced Wnt signature (Table S6).

### In the absence of exogenous Wnt ligands, SS18-SSX1-mediated *AXIN2* induction does not require the LEF-1/β-catenin complex

**Figure 2:**
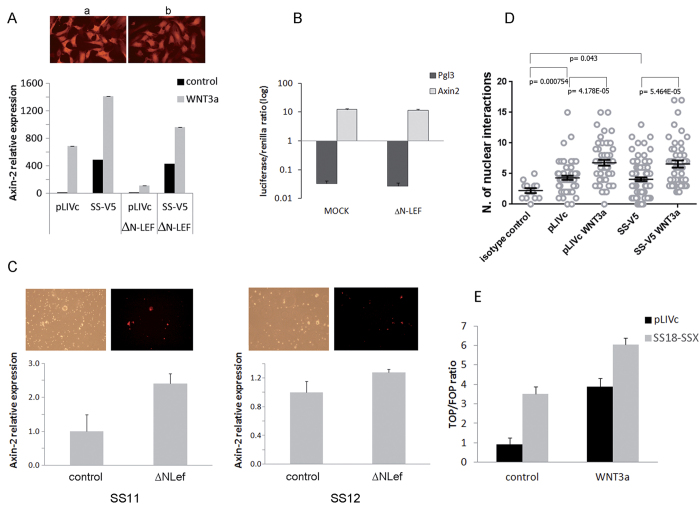
SS18-SSX1-dependent *AXIN2* promoter activity does not involve the LEF-1 β–catenin activation complex. (**A**) (upper panel) Fluorescence microscopy images of C3H10T1/2^*pLIVc*^ (a) and C3H10T1/*2*^*SS18-SSX1-V5*^ (b) cells infected with a lentiviral vector expressing both ΔNLEF-1 and RFP, 200X magnification; (lower panel) *AXIN2* message was assessed by Q-PCR after 24 hr stimulation with 100 ng/ml recombinant Wnt3a or PBS. (**B**) *mAXIN2* promoter activity in C3H10T1/*2*^*SS18-SSX1-V5*^ infected with ΔNLEF-1 or control vector. The ratio of *firefly* to *renilla* luciferase activity is reported on a logarithmic scale. Results are representative of three independent experiments. Error bars represent the SD of triplicate tests. (**C**) (upper panels) Fluorescence and bright field microscope images of cells derived from fresh SS samples infected with a lentiviral vector expressing both ΔNLEF-1 and RFP, 200X magnification; (lower panels) *AXIN2* message assessed by Q-PCR. (**D**) Graphical representation and statistical analysis of PLA using anti-β−catenin (mouse) and anti-LEF-1(goat) antibodies in unstimulated or 24 hr recombinant Wnt3a-stimulated C3H10T1/2^*pLIVc*^ (pLIVc) and C3H10T1/*2*^*SS18-SSX1-V5*^ (SS-V5) cells. PLA signal quantification was performed as described in materials and methods. Bars represent the S.E.M. (**E**) Luciferase activity after transient transfection with TOP- and FOP-Flash plasmids of resting or 24 hr recombinant Wnt3a-stimulated C3H10T1/2^*pLIVc*^ (pLIVc) and C3H10T1/*2*^*SS18-SSX1-V5*^ (SS-V5) cells. Results are reported as the ratio of TOP-Flash:FOP-Flash activity and are representative of three independent experiments. Error bars represent the SD of triplicate tests.

*AXIN2* is a direct target of a complex that includes TCF/LEF transcription factors and β-catenin^29^. To determine whether the β-catenin –TCF/LEF complex is implicated in *AXIN2* induction by SS18-SSX1, we expressed a dominant negative (ΔN) mouse LEF-1 mutant, which lacks the β-catenin binding domain but retains intact DNA binding activity^35^, in C3H10T1/2^*pLIVc*^ and C3H10T1/*2*^*SS18-SSX1-V5*^ cells. Lentiviral infection efficiency was assessed by red fluorescent protein (RFP) signal intensity co-expressed by the same bicistronic vector (Fig. 2A upper panel), and *AXIN2* mRNA levels were determined by qRT-PCR (Fig. 2A lower panel). A <10% decrease in *AXIN2* mRNA levels was observed in the presence of ΔN LEF-1 in C3H10T1/*2*^*SS18-SSX1-V5*^ cells (Fig. 2A black histograms), meaning that the robust induction of *AXIN2* by SS18-SSX was not significantly affected. The same held true for other Wnt target genes induced by SS18-SSX, including *LGR5, EDN1, RHOU, NRP2* and *DACT1* (data not shown). To verify that ΔN LEF-1 inhibits LEF-1/β-catenin complex activity at the *AXIN2* promoter in C3H10T1/2 cells, we compared *AXIN2* mRNA in C3H10T1/2^*pLIVc*^ and C3H10T1/*2*^*SS18-SSX1-V5*^ cells expressing or not ΔN LEF-1 stimulated with recombinant mouse Wnt3a (Fig. 2A gray histograms). *AXIN2* message was induced roughly 700 fold by Wnt3a in C3H10T1/2^*pLIVc*^ cells and the induction was strongly blunted by ΔN LEF-1 (Fig. 2A). In C3H10T1/*2*^*SS18-SSX1-V5*^ cells, Wnt3a stimulation increased SS18-SSX-induced *AXIN2* expression about 3 fold. The observed increase was attenuated by ΔN LEF-1 but *AXIN2* expression remained elevated, consistent with the lack of a significant inhibitory effect of ΔN LEF-1 on SS18-SSX-dependent *AXIN2* induction (Fig. 2A). Accordingly, the luciferase reporter system described above in C3H10T1/*2*^*SS18-SSX1-V5*^ cells expressing either ΔN LEF-1 or an empty vector showed that the robust luciferase activity induced by SS18-SSX1 was unaffected by the presence of ΔN LEF-1 (Fig. 2B). In primary SS11 and SS12 cells, ΔN LEF-1 not only failed to repress but tended to enhance *AXIN2* expression (Fig. 2C).

Interaction between β-catenin and LEF-1 was assessed *in vivo* in C3H10T1/2^*SS18-SSX-V5*^ and C3H10T1/2^*pLIVc*^ cells by proximity ligation assay (PLA), using a polyclonal goat anti-LEF-1 and a mouse anti-β-catenin antibody. Cells stimulated with recombinant mouse Wnt3a for 16 hours provided a positive control. In baseline conditions, the number of fluorescent foci, that represent interactions, in C3H10T1/*2*^*SS18-SSX1-V5*^ was comparable to that in resting C3H10T1/2^*pLIVc*^ cells (Fig. 2D). The same held true for interactions between β-catenin and TCF3/4 as assessed using rabbit anti-β-catenin and mouse anti-TCF3/4 antibodies (data not shown). The number of foci increased comparably in the nuclei of Wnt3a stimulated C3H10T1/*2* and C3H10T1/*2*^*SS18-SSX1-V5*^ cells (Fig. 2D). SS18-SSX expression in C3H10T1/2 cells therefore does not prevent β-catenin – TCF/LEF complex formation in response to Wnt ligands.

To verify that TCF/LEF participate in SS18-SSX1-mediated induction of *AXIN2* mRNA, we measured transcriptional TCF/LEF activity in C3H10T1/2^*pLIVc*^ and C3H10T1/*2*^*SS18-SSX1-V5*^ stimulated with 0.1 μM recombinant WNT3a or vehicle (PBS), using TOP-FLASH luciferase reporter and corresponding negative control FOP-FLASH plasmids (Fig. 2E). TOP luciferase activity was low in resting C3H10T1/2^*pLIVc*^ cells, consistent with negligible Wnt signaling. By contrast, C3H10T1/*2*^*SS18-SSX1-V5*^ cells displayed an increase in the TOP/FOP reporter ratio, comparable to that induced by WNT3a stimulation of parental cells. Combined SS18-SSX expression and WNT3a stimulation had a synergistic effect. Together with luciferase-based assay results that showed no effect of ΔN LEF-1 on SS18-SSX-dependent *AXIN2* expression (Fig. 2B), these observations suggest that LEF-1 may participate in SS18-SSX-mediated induction of *AXIN2* without binding to β-catenin. We therefore interrogated β-catenin implication in SS18-SSX-dependent *AXIN2* expression in the absence of exogenous Wnt signals.

**Figure 3:**
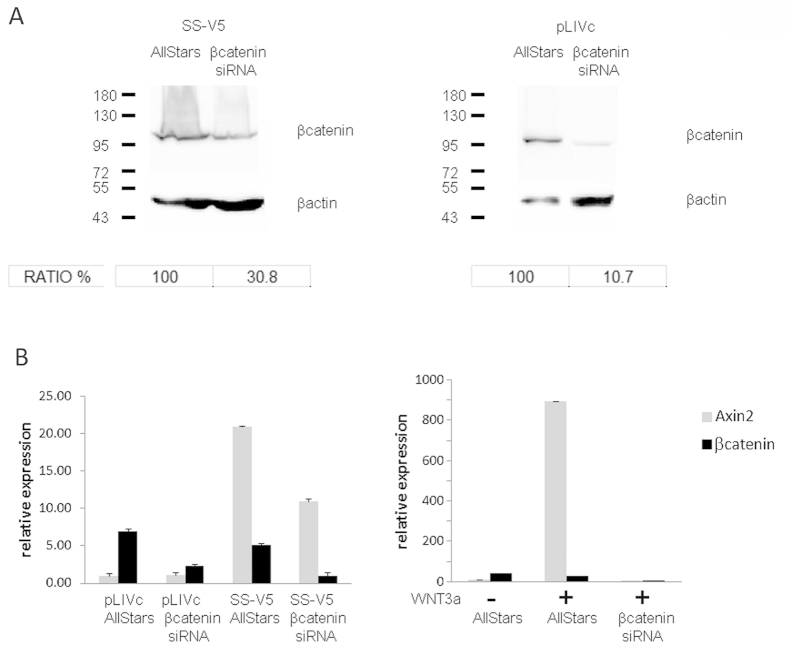
β−catenin participates in SS18-SSX1-induced *AXIN2* promoter activity. C3H10T1/2^*pLIVc*^ (pLIVc) and C3H10T1/*2*^*SS18-SSX1-V5*^ (SS-V5) cells were depleted of β−catenin using a pool of siRNAs or control siRNA (AllStars). Depletion was verified by Western blot and densitometric analysis using imageJ (Fig. 3A) or by qPCR (Fig. 3B). The effect on *AXIN2* transcript levels was measured by Q-PCR (Fig. 3B).

Depletion of β-catenin was achieved using either a pool of siRNAs (Fig. 3) or inducible shRNA (not shown) and its effect was tested on both SS18-SSX and WNT3a mediated *AXIN2* induction in C3H10T1/2 cells. The siRNA pool produced robust depletion of the protein as assessed by Western blot analysis (Figure 3 panel A), but the depletion was slightly less efficient in cells expressing SS18-SSX. In the presence of SS18-SSX a 75–80% decrease in β-catenin mRNA, as assessed by qRT-PCR, resulted in a 50% decrease in *AXIN2* message (Figure 3 panel B). In Wnt3a stimulated cells β-catenin depletion, assessed at 95% by qRT-PCR, resulted in the return of *AXIN2* expression to baseline levels (Figure 3B right panel). Similar results were obtained using inducible shRNA in SS18-SSX expressing cells (not shown).

Together these observations suggest that whereas SS18-SSX does not prevent β-catenin-TCF/LEF association in response to Wnt ligand stimulation, in the absence of Wnt ligands it induces TCF-LEF-dependent *AXIN2* expression without involving β-catenin-TCF/LEF complexes. Nevertheless, β-catenin may contribute to SS18-SSX-induced *AXIN2* expression, possibly by promoter de-repression.

### *AXIN2* repressor complexes in C3H10T1/2 cells

Although the four TCF family members (TCF1, TCF3, TCF4 and LEF-1) share the same protein interaction domains, mounting evidence suggests that each member has individual properties that may be responsible for unique functions in defined contextual settings^28^,^36^. Thus, the same TCF/LEF family members may behave as transcriptional repressors or activators in different cell types and may transition from transcriptional repressors to transcriptional activators in response to Wnt ligands. In addition, inactivation alone of their repressor activity has been reported to suffice to drive target gene expression^36^. We therefore addressed the implication of each TCF family member in transcriptional repression and activation of *AXIN2* in resting, Wnt3a-stimulated and SS18-SSX-expressing C3H10T1/2 cells and then assessed the implication of HDAC in *AXIN2* repression.

#### TCF/LEF family members

**Figure 4:**
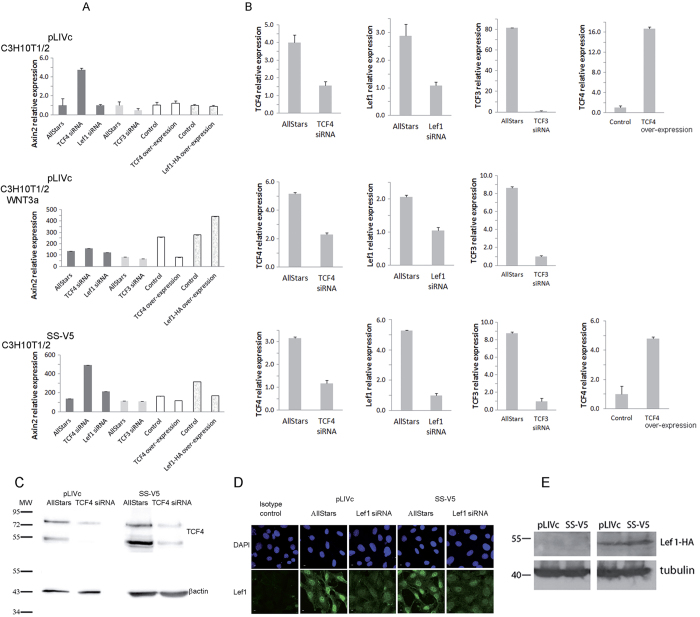
Implication of TCF/LEF family members in regulating *AXIN2* expression. (**A**) Q-PCR analysis of *AXIN2* in unstimulated or Wnt3a-stimulated (0.1 μM, 24 hrs) C3H10T1/2^*pLIVc*^ (pLIVc) and C3H10T1/*2*^*SS18-SSX1-V5*^ (SS-V5) cells depleted of or over-expressing individual TCF members as indicated. Depletion was achieved using a pool of specific or control siRNA (AllStars). (**B)** (first 3 columns): Depletion of each TCF/LEF (as indicated) was verified in each cell population by Q-PCR. B (right columns): Over-expression of TCF4 was measured by Q –PCR. (**C**) TCF4 depletion was verified by Western blot analysis using an anti-TCF4 antibody and anti-βactin as loading control; (**D**) LEF1 depletion was verified by immunofluorescence microscopy using an anti-LEF1 antibody. (**E**) Over-expression of HA-tagged LEF1 was assessed by Western blot analysis using an anti-HA antibody and anti-tubulin antibody as loading control.

The implication of TCF/LEF family members in *AXIN2* expression in C3H10T1/2 cells was addressed by their individual depletion or overexpression (Fig. 4). TCF1 was neither expressed nor induced by SS18-SSX in C3H10T1/2 cells (not shown) and was not considered further. Depletion of TCF3 and TCF4/LEF-1was achieved using a pool of 4 and pools of 3 siRNAs, respectively. HA-tagged LEF-1 and TCF4 were over-expressed using lentiviral infection and lipid-mediated transfection, respectively. Depletion and over-expression were assessed by qRT-PCR and Western blot analysis (TCF4 and LEF-HA, Fig. 4C,E) and by immunofluorescence (endogenous LEF-1, Fig. 4D).

In the absence of exogenous stimuli, changes in LEF-1 expression levels did not affect *AXIN2* expression significantly (Fig. 4A top panel). Over-expression of TCF4 also had no effect but its depletion consistently induced *AXIN2* (Fig. 4A top panel). By contrast, TCF3 depletion caused mild AXIN2 repression, below statistical significance. These observations suggest that in resting C3H10T1/2 cells complexes containing TCF4 participate in *AXIN2* promoter silencing whereas TCF3 and LEF-1 do not appear to be implicated in any significant manner.

Upon Wnt3a stimulation of C3H10T1/2 cells TCF4 continued to exert repression as reflected by moderate *AXIN2* up- and downregulation following its depletion and overexpression, respectively (Fig. 4A middle panel). LEF-1 and TCF3 by contrast participated in activation as depletion of TCF3 decreased *AXIN2* expression and LEF1 over-expression enhanced it (Fig. 4A middle panel).

In the presence of SS18-SSX, TCF4 displayed similar activity: its depletion increased whereas its over-expression mildly repressed *AXIN2* expression (Fig. 4A lower panel). In contrast to WNT3a stimulation, LEF-1 was associated with a repressor function in the presence of SS18-SSX, as LEF-1 depletion increased whereas LEF1-HA over-expression decreased *AXIN2* expression (Fig. 4A lower panel). TCF3 did not play a relevant role.

#### Histone deacetylase

HDAC involvement in *AXIN2* promoter silencing in C3H10T1/2 cells was verified by analysis of Histone H3 lysine9 acetylation (H3K9^Ac^) and by assessing the effect of the HDAC inhibitor trichostatin A (TSA) on *AXIN2* transcripts (Fig. S2). Treatment for 6 (or 16) hrs with 250 nM TSA induced a 6 fold increase in *AXIN2* mRNA in C3H10T1/2 cells, whereas in resting STO cells, which display a 12 fold higher *AXIN2* expression (Fig. S2B), the same treatment produced only a 2 fold increase (Fig. S2A). Comparison of H3K9^Ac^ at the *AXIN2* promoter in these two cell types revealed a higher content in STO cells (Fig. S2C). Thus, HDAC participates in regulating *AXIN2* promoter repression in resting C3H10T1/2 cells.

### *In vivo* validation of interactions implicated in the formation of complexes that regulate AXIN2 promoter activity

**Figure 5:**
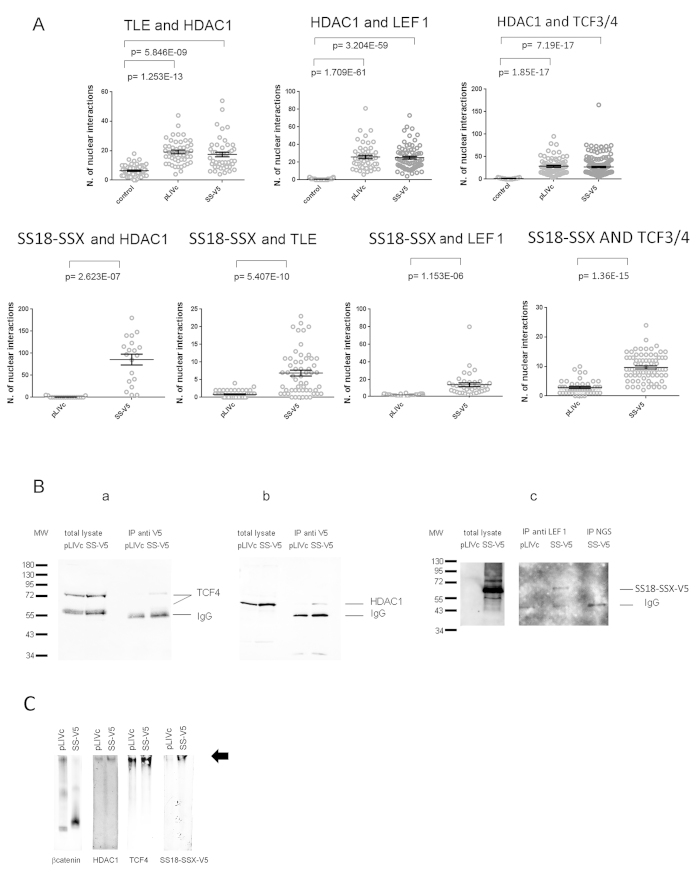
Interactions among SS18-SSX, HDAC1, TCFs, TLE and LEF-1. (**A**) Graphical representation and statistical analysis of PLAs using antibodies against the indicated proteins in C3H10T1/2^*pLIVc*^ (pLIVc) and C3H10T1/*2*^*SS18-SSX1-V5*^ (SS-V5) cells. PLA signal quantification was performed as described in materials and methods. Corresponding representative fluorescence microscopy images are shown in Fig. S3. (**B**) Immunoprecipitation (IP) was done on C3H10T1/2^*pLIVc*^ (pLIVc) and C3H10T1/*2*^*SS18-SSX1-V5*^ (SS-V5) cell lysates using anti-V5 monoclonal antibody (a and b), normal goat serum (NGS) or a goat polyclonal α-LEF-1 antibody (c). Immunoprecipitates were subjected to 10% SDS-PAGE together with total lysates and revealed using anti- TCF4 (a), anti-HDAC1 (b) or monoclonal α-V5 antibody (c).TCF4, HDAC1, SS18-SSX-V5, LEF1, immunoglobulins and protein size markers are indicated. (**C**) Native gel electrophoresis of C3H10T1/2^*pLIVc*^ (pLIVc) and C3H10T1/*2*^*SS18-SSX1-V5*^ (SS-V5) cell lysates. Equal amounts of each sample were loaded onto four wells on the same 5% gel, subjected to electrophoresis and transferred onto nitrocellulose membranes. After blotting the nitrocellulose membrane was cut and incubated with the indicated antibodies. The arrow indicates the co-migration position of HDAC1, TCF4 and SS18-SSX-V5.

*In vivo* interactions between HDAC and LEF-1, HDAC and TLE and TLE and LEF-1 were analyzed by PLA using rabbit anti-HDAC, goat anti-LEF-1 and mouse or rabbit anti-TLE antibody (Fig. 5A upper panel and Fig. S3). Assessment of the distribution of TLE, LEF-1, TCF3/4 and HDAC by immunofluorescence confirmed nuclear localization of TLE, prevalent nuclear localization of LEF-1and TCF3/4 and nuclear and cytoplasmic localization of HDAC (Figs S4 and 4D). Anti-LEF-1 and anti-HDAC and anti-TCF3/4 and anti-HDAC antibodies revealed a high number of foci in 100% of cells, as did anti-TLE and anti-HDAC antibodies (Fig. 5A upper panel), whereas the number of foci associated with anti-TLE and anti-LEF-1 antibodies was barely above the background (Fig. S3). Repression of the *AXIN2* promoter in C3H10T1/2 cells thus appears to be maintained primarily by HDAC/LEF-1, HDAC/TCF3/4 and HDAC/TLE-containing complexes. We therefore explored SS18-SSX association with the different repressor and activator complex components *in vivo*.

### SS18-SSX interacts with TLE, LEF-1, TCF3/4 and HDAC but not with β−catenin

*In vivo* association of SS18-SSX with complex components implicated in *AXIN2* promoter silencing was assessed by PLA (Fig. 5A lower panel, Figs S3 and S4). Anti-TLE1, 2, 3, 4 and anti-V5 antibodies produced nuclear foci significantly above the background but only in about 30% of cells, far below the fraction of SS18-SSX-V5 positive cells. Anti-V5 and anti-HDAC1 antibodies generated a high number of nuclear foci in all of the cells expressing SS18-SSX-V5, consistent with robust association between the fusion protein and HDAC. Interactions between SS18-SSX and LEF-1 were detected in C3H10T1/2^*SS18-SSX1-V5*^ cells stably expressing C-terminal HA-tagged LEF-1(Fig. 4E and data not shown) and association between SS18-SSX and endogenous LEF-1 was confirmed by comparison of PLAs in C3H10T1/2^*pLIVc*^ and C3H10T1/*2*^*SS18-SSX1-V5*^ cells using mouse anti-V5 and goat-anti LEF-1 antibody (Fig. 5A lower panel and S3). Rabbit anti-V5 and mouse anti TCF3/4 antibody revealed interactions between SS18-SSX and endogenous TCFs as well (Fig. 5A lower panel and S3). However, anti-β-catenin and anti-V5 antibody did not show increased numbers of foci compared to the background, suggesting absence of SS18-SSX-β-catenin interaction in C3H10T1/2 cells (Fig. S3). Importantly, all of the interactions observed in C3H10T1/2 cells were context-independent and were detected in the synovial sarcoma cell lines FUJI and HS-SYII expressing a V5-tagged SS18-SSX1 protein (Fig. S5) as well as in STO fibroblasts (data not shown).

All of the observed interactions were then tested *in vitro*. Co-immunoprecipitation from C3H10T1/2^*pLIVc*^ and C3H10T1/*2*^*SS18-SSX1-V5*^ cell lysates was performed using either an anti-V5 (Fig. 5B,a,b) or an anti-LEF-1 antibody (Fig. 5B,c). Western blot analysis revealed HDAC1and TCF4 in anti-V5 antibody immunoprecipitates and SS18-SSX1 in anti-LEF-1 antibody immunoprecipitates.

The relationship between β-catenin and SS18-SSX-containing complexes was further assessed using native gel electrophoresis of C3H10T1/2^*pLIVc*^ and C3H10T1/2^*SS18-SSX*^ and probing the resulting blots with anti-TCF4, anti-HDAC, anti-V5 and anti-β-catenin antibodies (Fig. 5C). In resting C3H10T1/2^*pLIVc*^ cells β-catenin, TCF4 and HDAC1 co-migrated in the gel consistent with their participation in the same complex. The same approach in C3H10T1/2^*SS18-SSX*^ cells revealed that SS18-SSX co-migrates with TCF4 and HDAC but not with β-catenin, which migrates to a distinct location in the gel. These observations support the existence of SS18-SSX-associated complexes that contain both HDAC and TCF from which β-catenin is excluded.

### SS18-SSX, TCF4 and HDAC are enriched at the *AXIN2* promoter

**Figure 6:**
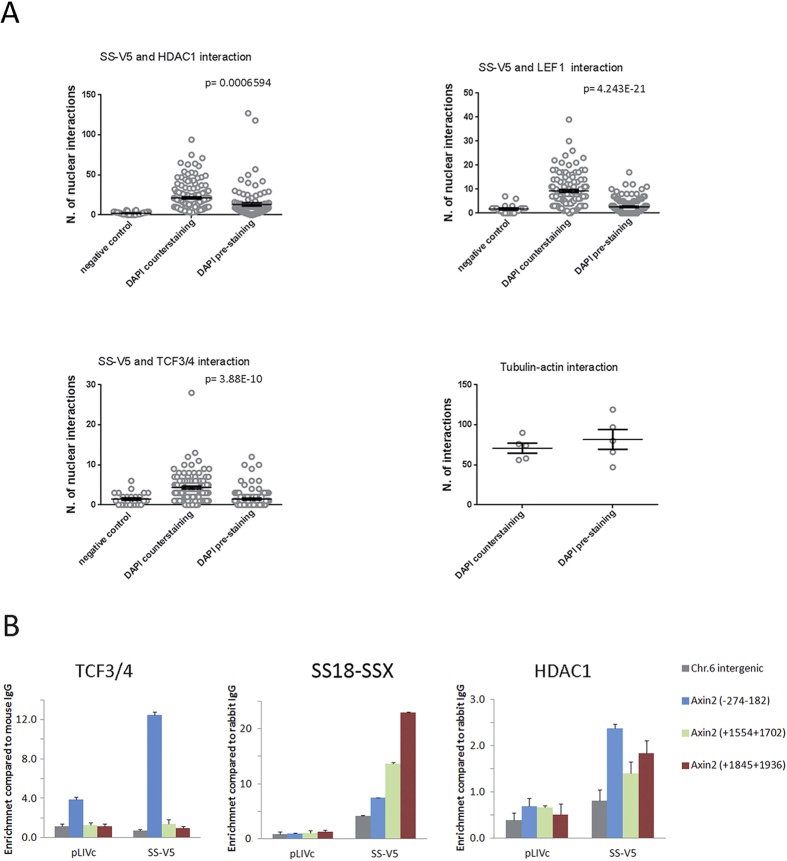
DNA dependence of SS18-SSX interactions. (**A**) Graphical representation and statistical analysis of PLAs using antibodies against the indicated proteins. C3H10T1/*2*^*SS18-SSX1-V5*^ cells were pre-stained with 5 μM DAPI for 30 min and subjected to PLA or subjected to PLA and then counterstained with DAPI. PLA signal quantification was performed as described in materials and methods. (**B**) ChIP using anti-TCF3/4, anti-V5 and anti-HDAC1 antibodies in C3H10T1/2^*pLIVc*^ and C3H10T1/*2*^*SS18-SSX1-V5*^ cells. Results are expressed as fold enrichment of values obtained with rabbit or mouse IgG immunoprecipitates, after normalization for the total amount of input chromatin. Results are representative of three independent experiments. Error bars represent the SD of triplicate PCR tests.

We next asked whether the observed associations are DNA-dependent. PLAs using paired antibodies for the identified interactors were conducted in C3H10T1/2^*pLIVc*^ and C3H10T1/*2*^*SS18-SSX1-V5*^ cells followed by DAPI counterstaining or in cells pre-stained with 5 μM DAPI to disrupt DNA prior to fixation. DAPI penetration of living cells was verified by fluorescence microscopy and DNA-independent interactions between actin and tubulin were assessed to exclude possible effects of DAPI pre-staining on PLA itself (Fig. 6A). Immunofluorescence staining of SS18-SSX, HDAC, LEF-1 and TCF3/4 using the same antibodies was done in parallel in both conditions to ascertain that DAPI pre-staining did not affect antibody reactivity (Fig. S6). PLA revealed that SS18-SSX association with HDAC, LEF-1 and TCF3/4 was strongly reduced in cells pre-treated with DAPI consistent with DNA-dependence of all of the observed interactions (Fig. 6A).

SS18-SSX, TCF3/4 and HDAC presence at the *AXIN2* promoter was verified by chromatin immunoprecipitation (ChIP) using rabbit anti-V5, anti-HDAC and anti-TCF3/4 antibodies (Fig. 6B). Capture of the entire complex was optimized using a dual crosslinking protocol for HDAC ChIP with 1% PFA and ethylene glycol-bis (EGS)^37^ or an SDS-free lysis buffer. Enrichment of SS18-SSX was observed in all regions analyzed. In the presence of SS18-SSX, TCF3/4 enrichment occurred primarily at the region upstream of the first ATG close to the T2 TCF/LEF binding site (−274 to −182), whereas HDAC enrichment was also observed downstream, within the region containing the T6 TCF/LEF binding site (+1554 +1936)^29^. Together, these observations indicate DNA-dependence of SS18-SSX interaction with HDAC and TCF/LEF and association of the resulting complexes with the *AXIN2* promoter.

### SS18-SSX does not dissociate repressor complexes

Assessment of possible SS18-SSX-mediated disruption of repressor complexes was conducted using PLA to compare interactions between LEF-1 and HDAC, TCF3/4 and HDAC, TLE and HDAC and TLE and LEF-1 in C3H10T1/2^*pLIVc*^ and C3H10T1/*2*^*SS18-SSX1-V5*^ cells (Fig. 5A upper panel). SS18-SSX expression did not significantly alter interactions between HDAC and LEF-1, TCF3/4 or TLE. Wnt3a stimulation of C3H10T1/2^*pLIVc*^ (0.1 μM for 16 h) did not result in significant dissociation of HDAC from LEF-1(not shown) whereas it increased association between LEF-1 and β-catenin, as discussed earlier (Fig. 2D). *In vivo* observations using PLA therefore suggest ternary HDAC, LEF-1 and β-catenin complex formation upon Wnt3a stimulation and HDAC, LEF-1 and SS18-SSX complex formation in C3H10T1/*2*^*SS18-SSX1-V5*^ cells in the absence of Wnt ligands.

### SS18-SSX enhances nuclear β-catenin translocation and degradation

Because β-catenin does not interact with SS18-SSX *in vivo* yet affects SS18-SSX-dependent induction of *AXIN2* even in the absence of Wnt ligands, albeit not as part of a complex with TCF/LEF1, we addressed its fate in SS18-SSX expressing cells. Localization of β-catenin was primarily cytoplasmic in resting C3H10T1/2^*pLIVc*^ cells (Fig. S7A) but mostly perinuclear and less markedly nuclear in C3H10T1/*2*^*SS18-SSX1-V5*^ cells. Nuclear β-catenin localization was comparable in C3H10T1/*2*^*SS18-SSX1-V5*^ cells, parental C3H10T1/2 cells stimulated for 24 hrs with recombinant murine Wnt3a, unstimulated SS11 cells and SS18-SSX-expressing STO fibroblasts (Fig. S7A). As enhancement of β-catenin translocation to the nucleus occurs in NIH3T3^7^ and STO cells (Fig. S7A) where SS18-SSX does not induce *AXIN2* message, nuclear anti-β-catenin antibody staining alone does not predict transcriptional effects.

The moderate nuclear β-catenin accumulation in C3H10T1/*2*^*SS18-SSX1-V5*^, Wnt3a stimulated C3H10T1/2 and SS11 cells despite enhanced translocation, may reflect increased nuclear degradation. Accordingly, GSK3β-dependent phosphorylation of β-catenin on Thr41, Ser33 and Ser37, which precedes proteosomal degradation^38^, was increased in the presence of SS18-SSX1 (Fig. S7B,b). Treatment with the proteasome inhibitor MG132 restored β-catenin protein levels in C3H10T1/*2*^*SS18-SSX1-V5*^ to those observed in wt and C3H10T1/2^*pLIVc*^ cells consistent with enhanced proteosomal degradation in C3H10T1/*2*^*SS18-SSX1-V5*^ cells (Fig. S7B, MG132). The increase in nuclear β-catenin resulting from SS18-SSX-stimulated translocation may therefore be restricted by correspondingly increased degradation, which may provide a mechanism for limiting nuclear β-catenin levels. Consistent with this notion and with recent observations by others^39^ we observed that β-catenin degradation in SS18-SSX-expressing cells was primarily nuclear (data not shown).

### SS18-SSX promotes nuclear β-catenin-HDAC interactions and affects HDAC activity at the *AXIN2* promoter

**Figure 7:**
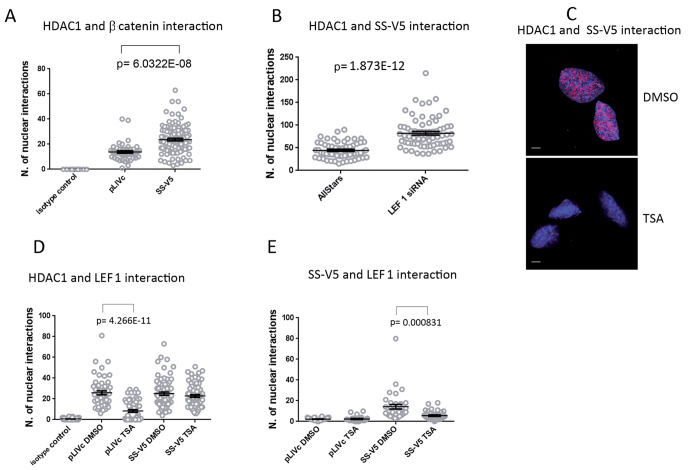
(**A**) SS18-SSX expression promotes interaction between β−catenin and HDAC1 in nuclei. Graphical representation with statistical analysis of PLAs using antibodies against the indicated proteins in C3H10T1/2^*pLIVc*^ (pLIVc) and C3H10T1/*2*^*SS18-SSX1-V5*^ (SS-V5) cells. Correspondent representative fluorescence microscopy images are reported in Fig. S7. (**B**) LEF-1 depletion promotes SS18-SSX-HDAC1 association. Graphical representation of PLA as in (**A**). (**C**) TSA treatment inhibits SS18-SSX association with HDAC1. Representative fluorescence microscopy images of PLA using anti-V5 and anti-HDAC1 antibodies in C3H10T1/*2*^*SS18-SSX1-V5*^ cells stimulated with 250 nM TSA or DMSO for 16hrs. Scale bar: 5 μm. (**D,E**) Effect of TSA treatment on LEF-1 interactions with HDAC (**D**) and SS18-SSX (**E**); graphical representation of PLA as in (**A**).

Based on the observation that β-catenin participates in SS18-SSX-dependent *AXIN2* induction, we interrogated the putative mechanism by which it does so. Nuclear β-catenin is suggested to promote transcriptional de-repression by removing HDAC from LEF-1 or by first disrupting TLE-LEF-1 interaction and then binding and inactivating HDAC^27^. PLA assessment of β-catenin interaction with HDAC in C3H10T1/2 cells in the presence and absence of SS18-SSX (Figs 7A and S8) using mouse anti-β-catenin and rabbit anti-HDAC1 antibody revealed both nuclear and cytoplasmic foci suggesting interaction in both compartments. However, C3H10T1/*2*^*SS18-SSX1-V5*^ cells displayed fewer cytoplasmic but significantly more nuclear foci than C3H10T1/2^*pLIVc*^ cells (Fig. S8A). Because HDAC localization, as assessed by immunofluorescence using anti-HDAC antibody, was comparable and predominantly nuclear in C3H10T1/2^*pLIVc*^ and C3H10T1/*2*^*SS18-SSX1-V5*^ cells (not shown), enhancement of nuclear β-catenin-HDAC1 interactions may be explained at least in part by the moderate SS18-SSX-dependent increase in nuclear β-catenin. As absence of β-catenin-SS18-SSX association suggests that the fusion protein is not part of a complex containing both HDAC and β-catenin, even moderately augmented nuclear β-catenin may help sequester HDAC from LEF-1 and thereby contribute to *AXIN2* promoter de-repression. β-catenin-dependent dissociation of the complex may facilitate subsequent SS18-SSX binding to HDAC and TCF/LEF-1. Consistent with this notion, LEF-1-depleted C3H10T1/*2*^*SS18-SSX1-V5*^ cells (Fig. 4D) subjected to PLA using anti-HDAC and anti-V5 antibodies revealed increased numbers of foci suggesting increased HDAC-SS18-SSX association upon LEF-1 depletion (Fig. 7B and S8).

Because HDAC plays a central role in TCF/LEF repression, we explored the structural basis of SS18-SSX-HDAC interaction and its functional consequences by comparing the effect of the class I and II HDAC inhibitor trichostatin A (TSA) on *AXIN2* transcripts in C3H10T1/2^*pLIVc*^ and C3H10T1/*2*^*SS18-SSX1-V5*^ cells (Fig. S2A). TSA blocks HDAC by binding to its catalytic site^40^. In the presence of SS18-SSX, HDAC inhibition by TSA was partially impaired as only a 3 fold increase in *AXIN2* message occurred in C3H10T1/*2*^*SS18-SSX1-V5*^ cells compared to a 6 fold increase in C3H10T1/2^*pLIVc*^ (Fig. S2A). The reduced efficacy of TSA in C3H10T1/*2*^*SS18-SSX1-V5*^ cells supports a model in which SS18-SSX binds to or modifies the catalytic site of HDAC recognized by TSA and impedes TSA binding. Consistent with this notion, 250 nM TSA markedly reduced the number of HDAC-SS18-SSX foci in C3H10T1/*2*^*SS18-SSX1-V5*^ cells suggesting that either SS18-SSX binds HDAC in the vicinity of its catalytic site and is displaced by TSA or that TSA binding alters HDAC conformation in a way that decreases SS18-SSX binding (Fig. 7C). TSA treatment also strongly inhibited HDAC-LEF-1 interaction in baseline conditions but failed to do so in the presence of SS18-SSX (Figs 7D and S8). PLAs using anti-V5 and anti-LEF-1 antibodies showed 30% reduction in the number of SS18-SSX/LEF-1 interactions, upon TSA treatment (Figs 7E and S8), suggesting their dependence on intact HDAC availability and supporting ternary complex formation.

### SS18-SSX expression in C3H10T1/*2* cells increases Histone H3 acetylation at the *AXIN2* promoter and induces changes in chromatin accessibility

**Figure 8:**
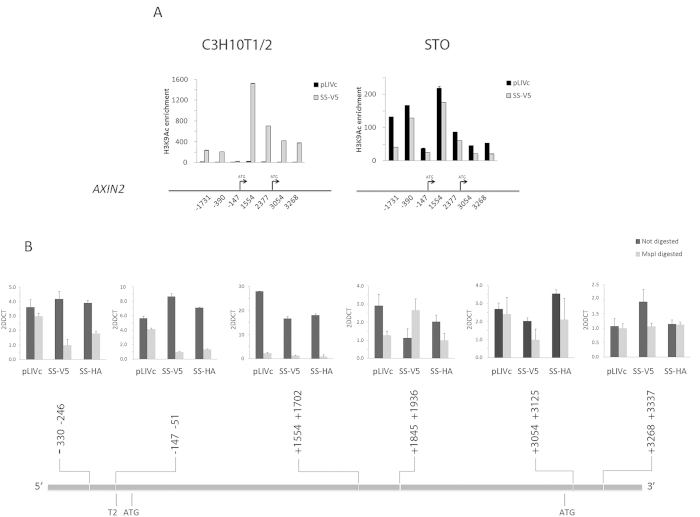
SS18-SSX promotes Histone H3K9 acetylation and chromatin accessibility at the *AXIN2* promoter. (**A**) Histone H3K9^Ac^ ChIP in C3H10T1/2^*pLIVc*^ (pLIVc), C3H10T1/*2*^*SS18-SSX1-V5*^ (SS-V5) and STO cells infected with SS18-SSX-V5 or empty pLIVc. Cross-linked chromatin was sonicated and immunoprecipitated with an anti Histone H3K9^Ac^ antibody or rabbit IgG. Co-immunoprecipitated DNA was quantified by real-time PCR using primer pairs annealing to the mouse *AXIN2* promoter region at the indicated positions. Results are expressed as fold enrichment of values obtained with rabbit IgG precipitates after normalization for the total amount of input chromatin. Results are representative of three independent experiments. Error bars represent the SD of triplicate PCR tests. (**B**) Chromatin accessibility tests in C3H10T1/2^*pLIVc*^ (pLIVc), C3H10T1/*2*^*SS18-SSX1-V5*^ (SS-V5) and C3H10T1/*2*^*SS18-SS1-HA*^ (SS-HA) cells. Positions of primers used for amplification of *MspI* CHART products by qRT-PCR are reported with approximate location of ATGs and transcription factor binding sites. Results are representative of three independent experiments. Error bars represent the SD of triplicate PCR tests.

By modulating HDAC activity, SS18-SSX may alter histone H3 acetylation and thereby participate in establishing a permissive chromatin structure for TCF-LEF-driven transcription. To address the effect of SS18-SSX on HDAC activity at the *AXIN2* promoter, chromatin immunoprecipitation (ChIP) was performed using anti-histone H3K9^Ac^ antibody and purified precipitated DNA was analyzed by qPCR using several primer pairs that anneal to different promoter regions (Table S7). Histone H3K9 acetylation underwent significant changes in response to SS18-SSX expression in C3H10T1/2 cells (Fig. 8A). Acetylation was increased at all the regions analyzed, particularly at those close to the TSS (exon 2) that contain the T4-T8 TCF/LEF binding sites (Fig. 8A, 1554–3268). By contrast, SS18-SSX expression in STO cells (Fig. 8A) resulted in a slight decrease in histone H3K9^Ac^. Thus, *AXIN2* promoter inducibility by SS18-SSX must depend, at least in part, on conditions that allow the fusion protein to affect acetylating and/or de-acetylating enzyme activity. Although these findings do not exclude the possible contribution of histone acetylases (HATs), they support the notion that HDAC activity at the *AXIN2* promoter is reduced upon SS18-SSX expression. Consistent with this notion treatment of C3H10T1/2^*pLIVc*^ and C3H10T1/*2*^*SS18-SSX1-V5*^ cells with 50 μM anacardic acid or 20 μM curcumin did not alter *AXIN2* expression excluding a major role for HATs (not shown). Histone acetylation leads to partial de-condensation of chromosomal domains that augments DNA accessibility and facilitates the action of the transcriptional machinery. To determine whether SS18-SSX1 may affect *AXIN2* transcription by altering chromatin conformation, chromatin accessibility assays were performed using methylation-independent restriction enzyme hydrolysis and real-time PCR (Fig. 8B). Primer pairs spanning sequences containing MspI sites were chosen within several regions of the *AXIN2* promoter and primer pairs spanning sequences of the *AXIN2* promoter that lack MspI sites were used as negative controls. *GAPDH*-specific primer pairs were used for normalization (Table S7). Compared to C3H10T1/2^*pLIVc*^, C3H10T1/*2*^*SS18-SSX1-V5/HA*^ cell-derived DNA displayed increased cleavage by MspI when primers spanning the regions –330 −246 and –147 −51 were used. These observations suggest that SS18-SSX induces histone acetylation with corresponding chromatin conformation changes at the *AXIN2* promoter.

## Discussion

We have shown cell context-independent SS18-SSX association with key transcriptional regulators of the Wnt pathway, including the transcription factors LEF-1 and TCF4 and the transcriptional repressors TLE and HDAC. In a permissive context, provided here by pluripotent mesenchymal C3H10T1/2 cells that may resemble candidate cells of origin of SS^41^,^42^,^43^,^44^,^2^,^45^, SS18-SSX induced a partial Wnt gene expression signature. Particularly striking was the robust induction of *AXIN2*, one of the least context-dependent Wnt target genes, reaching levels comparable to those observed in primary SS cells. Interrogation of the mechanistic basis for these observations revealed that SS18-SSX, TCF/LEF and HDAC are enriched at the same segment of the AXIN promoter, as demonstrated by ChIP analysis, and that SS18-SSX can induce the TOP-Flash reporter in the absence of Wnt ligand stimulation, supporting TCF/LEF-1 implication in SS18-SSX-associated transcriptional activation. However, SS18-SSX did not interact with β-catenin, despite enhancing its recruitment to the nucleus, and formed complexes with TCF/LEF, HDAC and TLE devoid of β-catenin. Expression of ΔN-LEF, which binds DNA but not β-catenin, failed to affect SS18-SSX-mediated induction of *AXIN2* as well as of *LGR5, EDN1, RHOU, NRP2 and DACT1* Wnt target gene expression, arguing against β-catenin-TCF/LEF complex involvement and suggesting that SS18-SSX may substitute for at least some β-catenin functions toward induction of TCF/LEF target genes.

In response to Wnt ligands, β-catenin translocates to the nucleus where it displaces or helps release TCF/LEF-associated HDAC and TLE/Groucho. It then binds TCF/LEF and promotes target gene induction by recruiting transcriptional activators^46^, as well as chromatin remodeling complexes including at the very least Brg1/SWI/SNF and p300CBP^47^. Similar to β-catenin, SS18-SSX not only binds TCF/LEF and HDAC but constitutes a part of the Brg1/SWI/SNF complex whose physiological activity is to create nucleosome-depleted regions at core promoters and regulatory sequences, facilitating transcription factor access to DNA. Thus, SS18-SSX and β-catenin may use analogous strategies to induce Wnt target genes, but elaborate distinct molecular complexes resulting in non-identical effects as illustrated by the partial Wnt signature induced by SS18-SSX. Although SS18-SSX and β-catenin do not associate physically, they appear to have a relevant functional relationship. Depletion of β-catenin in SS18-SSX expressing cells resulted in a 50% decrease in SS18-SSX-mediated upregulation of *AXIN2* expression suggesting retention of its role in de-repression of Wnt target gene promoters. By facilitating nuclear translocation of β-catenin, while excluding it from complexes that it forms with TCF/LEF, SS18-SSX may augment β-catenin availability for HDAC sequestration/inactivation, which is supported by increased nuclear HDAC-β-catenin interactions in SS18-SSX expressing cells, as shown by PLA.

Although SS18-SSX may utilize β-catenin to relieve promoter repression, our observations suggest that SS18-SSX itself participates in *AXIN2* promoter de-repression by inhibiting HDAC in ternary DNA-bound complexes. Formation of ternary complexes composed of SS18-SSX, HDAC and TCF/LEF is supported by co-migration of the three proteins in native gels, their presence in the same fractions of glycerol gradients (not shown) and the observation that HDAC-LEF1 interactions are unaltered by SS18-SSX expression. Moreover, the 30% reduction in the number of SS18-SSX-LEF-1 PLA foci following TSA treatment suggests partial dependence of the interaction on intact HDAC availability. The function of LEF-1 as transcriptional repressor in the presence of SS18-SSX also supports ternary complex formation. Nevertheless, SS18-SSX may also engage in binary complexes with LEF-1 and HDAC, as the increase in the number of HDAC-SS18-SSX interactions upon LEF-1 depletion suggests a dynamic equilibrium between complexes containing all three proteins and only SS18-SSX/HDAC or LEF-1/HDAC. Comparison of SS18-SSX/HDAC and LEF-1/HDAC with SS18-SSX/LEF-1 interactions suggests that this is indeed the case as the first two generate a much higher number of PLA foci than the last. The effect of β-catenin depletion on *AXIN2* expression raises the possibility that SS18-SSX may fulfill its function subsequent to β-catenin-mediated dissociation of HDAC from TCF/LEF-1. The increased SS18-SSX-HDAC interaction upon LEF-1 depletion suggests that SS18-SSX-HDAC association may precede ternary complex formation.

Despite binding Wnt transcription factors, SS18-SSX does not prevent Wnt ligand-induced target gene expression but rather appears to have a synergistic effect with canonical Wnt signaling. These observations are consistent with the possibility that SS18/SSX interacts with TCF/LEF at a fraction of their DNA binding sites on the *AXIN*2 promoter which it de-represses by blocking HDAC activity, inducing local chromatin changes and promoting nuclear translocation of available β-catenin to enhance HDAC inactivation/sequestration. If only a fraction of DNA-bound TCF/LEF-1 is occupied by SS18-SSX, stimulation by Wnt ligands can be expected to further augment *AXIN2* expression by activating canonical β-catenin-Wnt signaling. However, SS18-SSX interactions with TCF/LEF and TOP flash data that support TCF/LEF implication in SS18-SSX-dependent induction of *AXIN2* expression do not exclude the participation of a distinct SS18-SSX-associated transcription factor.

Taken together, our observations suggest that by interacting with TCF/LEF and utilizing β-catenin as a transcriptional de-repressor, SS18-SSX can induce a partial Wnt expression signature in the absence of Wnt ligands without excluding an additive effect of Wnt ligand-dependent stimulation. Synovial sarcoma cells may thus possess a dual mechanism of ensuring Wnt pathway activity that may be essential for defined stages of their development possibly including stemness maintenance and limiting differentiation. The ability of SS18-SSX to engage Wnt signaling without resorting to Wnt ligands may have important therapeutic implications at it suggests that mere impairment of canonical Wnt signaling may not suffice to abrogate SS development.

## Materials and Methods

### Antibodies and reagents

Antibodies used for western blot, immunoprecipitation, immunofluorescence or proximity ligation assay (PLA) were: mouse anti-V5epitope (Invitrogen Carlsbad, CA), rabbit polyclonal anti-V5 tag (Abcam, Cambridge UK), monoclonal anti-HA (Covance Research Product Inc Geneva Switzerland), mouse anti-Xpress (Invitrogen), mouse anti-tubulin (Calbiochem), rabbit anti-α/β tubulin (Cell Signaling), mouse anti-actin clone AC-40 (SIGMA), anti-TCF4 and anti-LEF-1 (Santa Cruz Biotechnology inc. Germany), rabbit anti-TCF4 (C48H11, Cell Signaling), anti-TCF3 + 4 [6F12-3], anti-Histone H3AcK9, anti-HDAC1 Ab7028, anti-HDAC1 Ab46985 and anti-TLE-1 (Abcam, Cambridge UK), anti-TLE1,2,3,4 (Cell Signaling), mouse anti-β-catenin (BD), β-catenin antibody sampler kit (Cell Signaling, Beverly MA). HRP-conjugated secondary antibodies were from Amersham (Goat anti-mouse-HRP) or from Dako, Denmark (goat anti-rabbit and rabbit anti-goat HRP). Fluorocrome-conjugated secondary antibodies were from molecular probes. TSA was from SIGMA, murine recombinant WNT3a from PreproTech, London UK.

### Ethical issues, cell lines, primary cells and SS samples

All experimental protocols were approved by the Ethics Committee of the University of Geneva (protocol 01-172) and of the Canton de Vaud (protocol 131/12) and were carried out in accordance with the approved guidelines. Written informed consent was obtained from all patients from whom tissues were obtained. C3H10T1/2 and STO cells were from ATCC and were cultured in DMEM, 10% FBS; HS-SYII synovial sarcoma cell line were obtained from Riken BioResource Center, Japan, FUJI synovial sarcoma cell line was a gift from Dr Francis Hornicek (Massachusetts General Hospital, Boston MA). Human mesenchymal stem cells were obtained as described^48^ and were cultured at low confluence in IMDM, 10% FCS, 10 ng/ml PDGF-BB (PeProTech, London, UK). Human satellite cells and human myoblasts were a gift of Dr Laumonier (University of Geneva, Switzerland) Human muscle samples, cell dissociation, and clonal culture from satellite cells were prepared as described^49^. Tumor specimens and normal tissues adjacent to cancerous lesions obtained after surgical resection, frozen and stored at −80 °C until use. SS11 and SS12 samples were mechanically dissociated and subjected to enzymatic digestion for 40 minutes at 37 °C in IMDM. Details of culture of these cells can be found in supplemental methods.

### Constructs and cDNA Cloning

cDNA clones, encoding human *SS18-SSX1* in frame with a V5 or HA tags were obtained using previously described constructs^50^ as templates for PCR amplification. The mouse *Axin-2* promoter sequence cloned into the pGL3 basic vector, p043 mTcf-4 B and pcDNA3.1/nV5-beta catenin were obtained from Adgene, SS18 and SS18-SSX mutants and HA-tagged mouse LEF-1 were constructed as described in supplemental methods. The LV-CRE-PLKO.1 and the PLKO.1 vector were from Addgene.

### Q-PCR

Q-PCR analysis was done using a HT-7900 instrument (life technology). Endogenous controls for normalization were mouse GAPDH, mouse and human cyclophilin and 18S. A ΔCT method and protocols for absolute or relative quantification (Applied Biosystems) were used.

### Lentivral infection, protein expression and knockdown

Expression of SS18-SSX1-V5, SS18-SSX1-HA, SS18-V5, *SS18*-1-412-V5, XP-SS18-SSX-161-491, LEF-1-HA was achieved using the self inactivating lentiviral Gene Transfer and Expression system pLIVc which produces a floxed proviral genome (Detailed information on pLIVc is available on demand). SSXfrag.-V5, mouse TCF4 and V5 tagged β-catenin expression were achieved by transfection using Xtreme gene 9 reagent (Roche) using standard transfection protocols. SiRNAs were transfected using Interferin siRNA transfection reagent (Polyplus). siRNAs used were s13510 and s13512 for SS18 (life technology), sc-35805 for mouse LEF1, sc-43526 for mouse TCF4 (Santa Cruz biotechnology), a pool of Mm-Tcf2a, 1,2,3,4 (QUIAGEN) for TCF3 and esiRNA MU-04762-1 (SIGMA)for β-catenin. The shRNA construct for β-catenin knockdown was as described^51^. ShRNA constructs for SS18-SSX were obtained either by cloning the sequences targeting the breakpoint (ATATGACCAGATCATGCCCAAG; TGGATATGACCAGATCATGCCC) in the lentiviral pLVShRNAmir plasmid or by cloning the sequence reported by Kadoch and Crabtree^31^ in the pLKO.1 lentiviral vector. Depletion was verified by qRT-PCR, Western blot or immunofluorescence.

### Affymetrix microarray and bioinformatic analysis

RNA extraction and quality assessment were performed as described^50^; quality-tested total RNA was used by the Lausanne Genomics Technology Facility (GTF) for gene expression profile analysis on Affymetrix Mouse Gene 1.0 ST Arrays (http://www.unil.ch/dafl/). Gene expression levels were obtained with RMA^52^ and differential expression was assessed using a >2 fold change cut off for SS18-SSX expression in C3H10T1/2 cells and limma for SS18-SSX expression in STO cells and WNT3a stimulation. Enrichment of various functional categories was determined with exact Fisher tests. The source of data for the enrichment analysis is reported in the supplemental methods.

### Transient transfection and luciferase assays

Cells were transfected with 2 μg of pGL3 vector or the pGL3-*Axin-2* promoter constructs along with a pGL4 *renilla* luciferase construct using X-tremeGENE 9 DNA transfection reagent (Roche) according to manufacturer’s recommendations. Transfection efficiency was ~60% as assessed using a pMAX-GFP control vector (AMAXA). Reporter *firefly* and *renilla* luciferase activity were measured in triplicate 48 hours later on cleared cell lysates using the dual luciferase assay system (Promega, Madison, WI, USA), according to manufacturer recommendations.

### TOP/FOP-flash assay

TOP/FOP-flash luciferase constructs were a gift from Dr Huelsken (EPFL Lausanne, Switzerland). 70% confluent cells in 6 well plates were transfected with TOP-flash or FOP-flash plasmid DNA along with a pGL4 *renilla* luciferase construct, using X-treme gene 9 transfection reagent. Reporter *firefly* and *renilla* luciferase activity were measured in triplicate 48 hours later on cleared cell lysates using the dual luciferase assay system (Promega, Madison, WI, USA).) as instructed by the manufacturer. After normalization of *firefly* to *renilla* luciferase activity data were represented as the ratio of TOP-flash:FOP-flash activity.

### Native gel electrophoresis

Cell lysates were prepared in lysis buffer (10 mM HEPES pH 7.9, 0.5 mM EDTA, 12.5 mM MgCl2, 1 mM DTT, 0.05% NP40) containing proteases and phosphatase inhibitors by performing 6 cycles of freeze/thawing and shearing DNA with a 22G needle. Equal protein amounts from cleared lysates after centrifugation at 14000 rpm for 10 min at **4 **°**C** were loaded onto a 5% native gel. After transfer to nitrocellulose identical lanes were separated and each protein separately revealed with appropriate primary and secondary antibodies. Proteins were also revealed in the same lanes using sequences of primary and secondary antibodies that guarantee no cross-reactivity, stripping the nitrocellulose after each detection and using secondary antibodies conjugated to different fluorochromes. Signals were revealed using Fusion FX (Vilbert-Loumat).

### Immunoprecipitation

Cell lysates for immunoprecipitation were prepared as above and incubated with anti-V5 conjugated agarose beads (SIGMA) for 3 hrs at **4 **°C. After washing, bound proteins were eluted by the addition of excess V5 peptide, subjected to SDS-PAGE and blotted with anti-HDAC1or anti-TCF4 antibody. For LEF-1 immunoprecipitation cells were lysed in RIPA buffer and incubated with 5 μg of antibody overnight at **4 **°**C**, and then with protein G-sepharose beads. After washing, bound proteins were eluted by boiling in sample buffer and analyzed on SDS-PAGE. After transfer to nitrocellulose mouse anti-V5 and HRP-goat anti-mouse antibodies were used.

### Chromatin immunoprecipitation

ChIP for Histone H3 AcK9 was performed according to Abcam protocols (Abcam, Cambridge, UK). Briefly 10^7^ cells were cross-linked with 1% folmaldehyde for 10 min. After addition of 0.125 M glycine and washing in PBS, cells were lysed and the chromatin fraction was sheared to roughly 600 bp fragments by sonication. About 1/15 of the lysate was stored as input DNA. Immunoprecipitation was performed using antibody ab7028 (Abcam) or rabbit immunoglobulins as negative control and herring sperm DNA blocked protein A-sepharose beads. Cross-linkage was reversed using proteinase K and DNA purified by phenol/chloroform extraction and ethanol precipitation. Quantitative PCR on the immunoprecipitate and on input DNA was performed on a ABI Prism 7700 instrument (Applied Biosystems). Primers complementary to several regions of the mouse Axin-2 promoter within residues –1731 to +3268 were designed using Assay Design Center ProbeFinder (Roche). Primer sequences are reported in Table S7.

### Proximity ligation assay (PLA) and Immunofluorescnce

Proximity ligation assay was performed using a Duolink II Fluorescence PLA kit (Olink Bioscience, Uppsala, Sweden) as instructed by the manufacturer. Cells were seeded at 70% confluence in 0.2 cm^2^ dishes, fixed in 4% paraformaldehyde in PIPES buffer for 13 minutes at RT and permeabilized with 0.3% triton in PBS for 3 minutes. Primary antibodies were used at the following dilutions: 1:2000 for mouse anti-V5, rabbit anti V5, monoclonal anti-HA, rabbit anti-HDAC1 Ab7028, mouse anti-β-catenin and rabbit anti-β-catenin. Anti-HDAC1 Ab46985 1:100, anti LEF-1 1:400, anti TLE-1and anti TLE1,2,3,4 1:100, anti TCF4:1:1000. PLA amplification was labeled with Alexa Fluor 594 (Olink Bioscience, Uppsala, Sweden). Slides were counterstained with DAPI, mounted and imaged using the Zeiss Confocal Fluorescent Microscope LSM710, with oil immersion objective 63X, NA 1.4. For each channel the pin hole was set to 0.9 AU. For each sample the Z-stack was acquired with a line averaging of 2 passages and with a sampling in the XYZ according to the optimal Nyquist criteria. Before analysis the Z-stack was converted with maximum intensity projection. The resulting images were analyzed using ImageJ software (http://rsbweb.nih.gov/ij/) with a script that defines as region of interest (ROI) the DAPI stained nuclei and counts the included PLA fluorescent foci (algorithm described in supplemental methods). For statistical analysis fluorescent foci were counted for each sample in 5 different fields each containing an average of 8–10 cells. Details can be found in supplemental methods.

### Chromatin accessibility tests (ChART-PCR)

ChART-PCR was performed as previously described with slight modifications^53^. Details can be found in supplemental methods.

## Additional Information

**How to cite this article**: Cironi, L. *et al.* The fusion protein SS18-SSX1 employs core Wnt pathway transcription factors to induce a partial Wnt signature in synovial sarcoma. *Sci. Rep.* **6**, 22113; doi: 10.1038/srep22113 (2016).

## Supplementary Material

Supplementary Information
