# Supplementary material for: The fusion protein SS18-SSX1 employs core Wnt pathway transcription factors to induce a partial Wnt signature in synovial sarcoma
Source: Sci Rep. 2016 Feb 24;6:22113. doi: 10.1038/srep22113 (PMC4764983; doi:10.1038/srep22113)
Supplement: Supplementary Information [file srep22113-s1.pdf]

The fusion protein SS18-SSX1 employs core Wnt pathway transcription factors to induce a partial Wnt signature in synovial sarcoma

Luisa Cironi, Tanja Petricevic, Victor Fernandes Vieira, Paolo Provero*, Carlo Fusco, Sandrine Cornaz, Giulia Fregni, Igor Letovanec, Michel Aguet and Ivan Stamenkovic

SUPPLEMENTARY DATA

Supplementary Figure S1

Specificity of SS18-SSX mediated Axin-2 induction. A) Schematic representation of V5 tagged SS18-SSX1 fusion protein and tagged SS18-SSX and SS18 truncated forms. B) Immunofluorescence analysis of C3H10T1/2 infected with the indicated tagged proteins, anti V5, anti Xpress, mouse immunoglobulins and an Alexafluor 488 goat anti mouse secondary antibody were used. Scale bar 5µm. C)Western blot analysis of C3H10T1/2 infected with V5-tagged SS18. Mouse anti V5, anti tubulin and HRP-conjugated goat anti mouse secondary antibody were used.
D) Quantitative RT-PCR analysis of Axin-2 transcripts in C3H10T1/2 infected with the indicated tagged proteins or an empty pLIVc vector. Cells were selected for 10 days with 1µg/ml puromycin. The analysis was done using the relative quantitation method (Applied Biosystems) and normalizing to an endogenous control (GAPDH). Results are representative of three independent experiments. Bar represent the SD of triplicate PCR tests.

Supplementary Figure S2

Divergent occupancy of Histone H3 AcK9 at the *Axin-2* promoter in C3H10T1/2 and STO. A) Q-PCR of *AXIN2* transcripts in C3H10T1/2^{pLIVc} (pLIVc), C3H10T1/2$^{SS18\text{-}SSX1\text{-}V5}$ (SS-V5) and STO cells infected with SS18-SSX-V5 or empty pLIVc. Cells were stimulated with 250nM TSA or DMSO for 16hrs. Results are represented as the 2DDCT ratio of TSA stimulated samples versus DMSO controls. B) Quantitative RT-PCR analysis of Axin-2 transcripts in C3H10T1/2 and STO cell lines. C) Chromatin immunoprecipitation detecting acetylated Histone H3 bound to the Axin-2 promoter. Cross-linked chromatin was sonicated and immunoprecipitated with an anti Histone H3 AcK9 antibody or rabbit IgG. Co-immunoprecipitated DNA was quantified by real-time PCR using primer pairs annealing at the mouse Axin-2 promoter region at the indicated

positions. The results are expressed as fold enrichment over the values obtained with rabbit IgG precipitates, after normalization for the total amount of input chromatin. Results are representative of three independent experiments. Error bars represent the SD of triplicate PCR tests.

Supplementary Figure S3

Representative fluorescence microscopy images of Proximity ligation assays (PLA) in C3H10T1/2^{pLIVc} (pLIVc) and in C3H10T1/2$^{SS18\text{-}SSX1\text{-}V5}$ (SS-V5) using antibodies against the indicated proteins. Only one of the two antibodies was used in the negative controls. Images were obtained with a confocal microscope as described in material and methods. Scale bar 5µm.

Supplementary Figure S4

Expression and localization of SS18-SSX-HA, SS18-SSX-V5, HDAC1, TLE and TCF3/4 proteins. Representative immunofluorescence microscopy images obtained using the indicated antibodies in C3H10T1/2 cells. Proteins are visualized with Alexa fluor488 conjugated secondary antibodies. Images were obtained with a confocal microscope as described in material and methods. Scale bar 5µm.

Supplementary Figure S5

SS18-SSX-V5 interactions with HDAC1, LEF1, TCF4 and TLE in synovial sarcoma cell lines. Representative fluorescence microscopy images of SS18-SSX-V5 immuno-staining (IF) and proximity ligation assays (PLA). FUJI and HS-SYII cells were infected with V5-tagged SS18-SSX1 lentiviral plasmids. For PLA a mouse anti-V5 antibody was used with either rabbit anti-HDAC1, anti-TCF4, anti-TLE1/2/3/4 or a goat anti-LEF1 antibody (as indicated). SS18-SSX-V5 staining was revealed using an alexa fluor 594-conjugated donkey anti-mouse antibody. Cells lacking the tagged protein provided negative controls. Scale bar: 5µm.

Supplementary Figure S6

Preservation of antibodies reactivity with DAPI pre-staining. C3H10T1/2 cells were pre-treated with 5µM DAPI or PBS for 30 minutes, fixed and stained with the indicated antibodies and alexa fluor 488 conjugated secondary antibodies. Representative Images, obtained with a confocal microscope, are shown. Scale bar 5µm.

Supplementary Figure S7

SS18-SSX expression changes β−catenin localization and promotes its degradation.

A) Representative fluorescence microscopy images of β−catenin in C3H10T1/2^{pLIVc} (pLIVc) and C3H10T1/2$^{SS18\text{-}SSX1\text{-}V5}$ (SS-V5) cells, C3H10T1/2 cells stimulated for 24 hrs with recombinant Wnt3a (WNT3a), STO, and primary SS11 cells. Cells were incubated with monoclonal anti-β-catenin antibody or mouse IgG1 as indicated, anti-mouse Alexa fluor 488- conjugated secondary antibody and counter-stained with DAPI. Images were obtained with a confocal microscope as described in material and methods. Scale bar 5µm.(B) Western blot analysis of β-catenin (a) and phospho β-catenin (b) in synchronized wild type C3H10T1/2 (wt), C3H10T1/2^{pLIVc} (pLIVc) and C3H10T1/2$^{SS18\text{-}SSX1\text{-}V5}$ (SS-V5) treated with 10µM MG132 or DMSO for 24 hrs. Mouse anti-tubulin antibody was used as loading control (c).

Supplementary Figure S8

Representative fluorescence microscopy images of Proximity ligation assays (PLA) reported in figure 7. Images were obtained with a confocal microscope as described in material and methods. Scale bar 5µm.

SUPPLEMENTARY EXPERIMENTAL PROCEDURES

Tumor specimens

Tumor samples SS11 and SS12 were mechanically fragmented and dissociated using enzymatic digestion for 40 minutes at 37°C in IMDM containing 0.5mg/ml collagenase type II, 0.5mg/ml collagenase type IV, 0.1mg/ml DNAse I, 1% Antibiotic-Antimycotic (Gibco- Life Technologies). After erythrolysis cells were passed through a 70µm cell strainer and cultured as spheres in IMDM (Life Technologies), supplemented with 20% knockout serum replacement (Life Technologies), 10 ng/mL of recombinant human epidermal growth factor (Invitrogen), 10 ng/mL of recombinant human basic fibroblast growth factor (Invitrogen), 10 ng/mL of leukemia inhibitory factor (Millipore) and 1% penicillin/streptomycin (Life Technologies).

Constructs and cDNA Cloning

cDNA clones, encoding the human *SS18-SSX1* fusion gene in frame with a V5 epitope tag.or in frame with an HA tag were obtained using previously described constructs [50] as template for PCR amplification. For SS18-SSX1-V5, primers used were: SS18-kzFw and SSXnoStopBamHI- R, a N-terminal fragment of *SS18-SSX1* resulting from BstBI digestion

was first inserted into the homemade pLIVc puro lentiviral expression vector into the PmeI blunt site and the BstBI site. Subsequently a C-terminal SS18-SSX1 fragment obtained by digestion with BstBI and BamHI was inserted at the corresponding sites in frame with the V5 epitope tag. For SS18-SSX1-HA primers used were: SS18-kzFw and EcoRI-stop-SSX-HA-R, the resulting fragments were inserted into the pLIVc puro lentiviral expression vector into the PmeI blunt site and the EcoRI site. cDNA clones, encoding the human *SS18* gene, the truncated form *SS18*-1-412-V5and the SSXfrag.-V5 were obtained using a TA cloning system (pEF6/V5-His TOPO, Invitrogen). *SS18*- V5 and *SS18*-1-412-V5 fragments were subsequently re-amplified using primers SS18-kzFw and V5-Stop-EcoRI-R and inserted into the pLIVc puro lentiviral expression vector into the PmeI blunt site and the EcoRI site. XP-SS18-SSX-161-491 template was obtained by sub-cloning into the Xpress pcDNA3.1his C vector (Invitrogen) cDNA fragments produced by cleaving at the BamHI sites a SS18-SSX1 clone previously inserted in the pEF6/V5-His TOPO vector (invitrogen) in the antisense direction. XP-SS18-SSX-161-491 was subsequently re-amplified using primers on T7 and MluI-stopSSX1-R and inserted into the pLIVc puro lentiviral expression vector into the PmeI blunt site and the MluI site. cDNA clone, encoding the mouse *LEF-1* genes in frame with a C-terminal HA tag was obtained by insertion into the pLIVc puro lentiviral expression vector into the PmeI blunt site and the EcoRI site. The dominant negative mutant of LEF-1 Δ*N-LEF-1* (NM_010703; nucleotides 1086–218), the TOP/FOP FLASH plasmids and the pLVRNAmir vector were a gift from Dr Huelsken (EPFL Lausanne, Switzerland). The mouse *Axin-2* promoter sequence from position –2954 to +2688 (Gene ID1203 , +1 is the start codon on exon 1) cloned into the pGL3 basic vector was obtained from Adgene (Adgene plasmid 21275). Integrity of all constructs was verified by sequencing.

RNA extraction and Real time quantitative PCR

Total RNA was isolated from frozen tumors using Trizol reagent (Invitrogen, Carlsbad, CA) according to the manufacturer's recommendations. An RNeasy Mini Kit (Qiagen, Hilden, Germany) was used for total RNA extraction from cultured cells. cDNA was obtained using an M-MLV reverse transcriptase and RNAse H minus (Promega). Typically 500 ng of template total RNA and 250 ng of random hexamers were used per reaction. Real time-PCR amplification was performed in an ABI Prism 7700 instrument (Applied Biosystems) using a Fast start Universal SYBR GREEN master mix (Roche). Primers used are reported in table S7. Relative quantitation of targets, normalized with an endogenous control (mGAPDH, hCyclophilin or 18S), was performed using a comparative Ct method (Applied Biosystems). In some cases the absolute quantitation method (Applied Biosystems) was used.

Affymetrix microarrays and bioinformatic analysis.

RNA extraction and quality assessment were performed as previously described (Cironi et al., 2009), quality-tested total RNA was used by the Lausanne DNA Array Facility (DAFL) to perform gene expression profile analysis on Affymetrix Mouse Gene 1.0 ST Arrays (C3H10T1/2 cells) and the UNI Lausanne Mouse 17K platform, described in the GEO database (http://www.ncbi.nlm.nih.gov/geo) under accession GPL4371 (STO cells),according to the manufacturer's recommendations (http://www.unil.ch/dafl/). Gene expression levels were obtained with RMA (Irizarry et al., 2003) using the Affymetrix Power Tools suite. Differential expression was determined by requiring a fold-change greater than 2 for SS18-SSX expression in C3H10T1/2 and using limma for SS18-SSX expression in STO cells and for WNT stimulation. Enrichment of various functional categories, detailed below, was performed by first translating the lists of differentially expressed probesets into lists of Entrez Gene ids using the annotation files provided by the manufacturer (version na30), and then performing exact Fisher tests. Gene Ontology (Ashburner et al., 2000) annotations were obtained from Entrez Gene (data downloaded march. 19, 2010). We used version 20100703 of the Gene Ontology. The imprinted genes listed by Geneimprint (http://www.geneimprint.com) and the Catalogue of Imprinted Genes at the University of Otago (Morison et al., 2005) http://igc.otago.ac.nz were downloaded from the corresponding web sites on June 15th 2010. Chromatin-associated proteins were downloaded from the chromdb site (Gendler et al., 2008) url on June 15th 2010. All gene symbols from these databases were converted into Entrez Gene ids using the gene_info file obtained from the Entrez Gene ftp site. A list of Wnt targets was obtained from http://www.stanford.edu/~rnusse/pathways/targets.html, considering only mammalian genes and using Homologene to identify orthologous genes in species. KEGG pathway data were downloaded on March 19th, 2010. A list of CpG islands in the mouse genome (sequence version mm9) was obtained from the UCSC genome browser site. We then used the TSS coordinates predicted by UCSC to determine which transcripts have their TSS lying in such CpG islands. This list was converted into a list of Entrez gene ids using the conversion table provided by UCSC. Sarcoma signatures of reference (Francis et al., 2007) were obtained from the manuscript's supplemental material. Embryonic stem cell signatures were downloaded from the supplemental material of reference (Ben-Porath et al., 2008). Human genes included in these lists were translated into mouse genes using the one-to-one orthology relationships defined by Homologene, build 64 (http://www.ncbi.nlm.nih.gov/homologene).

Chromatin accessibility tests (ChART-PCR)

Cells were detached with trypsin and washed twice with ice cold PBS. After counting 10^7 cells were resuspended in 900 µl of buffer containing: 10mM Tris-HCl, pH 7.4, 10mM NaCl and

3mM MgCl2 and left om ice for 10 minutes. After adding 10% NP-40 to 1% final concentration, cells were homogenized in a dounce homogenizer with 15 strokes. Nuclei were recovered by centrifugation at 700g for 10 minutes at 4C, washed once in the same buffer, than washed in a buffer containing: 10mM Tris-HCl, pH 7.9, 50mM NaCl, 10mM MgCl2, 1mM DTT and 0.3M sucrose, and resuspended in the same buffer. Aliquots of 1 milion nuclei were used for digestion reaction in a volume of 50 µl containing NEB buffer 4 and 60 U of MspI (New England Biolab, Ipswich, MA). Digestion was performed from 15 minutes to 1 h at 37C and was terminated by the addition of an equal volume of stop solution (20mM Tris-HCl, pH 7.9, 600mM NaCl, 1% SDS, 10 mM EDTA and 400µg/ml proteinase K) followed by incubation at 55 °C for 16 hours. Control samples in which MspI was not added (not digested) and digestion reactions in which 6 µg of purified genomic DNA was used were included. After digestion DNA was extracted with phenol/chloroform, ethanol precipitated and re-suspended in 100µl of TE by incubating at 70°C for 15 minutes. qPCR was performed in triplicate using 2 and 5 µl of DNA, relative quantitation of target, normalized to GAPDH control, was performed using a comparative Ct method (Applied Biosystems). Primer pairs used for PCR amplification, spanning several regions of the *Axin-2* promoter, are listed in table S7, primers amplifying a fragment not containing any MspI site were also included.

Immunofluorescnce

For βcatenin staining, cells, grown on slides were fixed for 20 min at -20° C in 88% methanol, 12% PBS and permeablized with phosphate buffer saline (PBS) containing 0.05% NP-40 for 5 minutes RT. For SS18-SSX, HDAC, TLE and LEF-1 staining cells were fixed for 15 min in 4% paraformaldehyde and permeablized with phosphate buffer saline (PBS) containing 0.1% Triton X-100 for 20 minutes. Cells were blocked in 5% bovine serum albumin in PBS for 30 min and incubated with primary antibody or isotype controls at RT for 1 hour. Dilutions used were: monoclonal anti-βcatenin (1:500), 1:200 for mouse anti V5, anti Xpress and anti HDAC1, 1:50 for anti LEF-1, 1:100 for TLE1,2,3,4. Alexa fluor 488 conjugated secondary antibodies (molecular probes) were used for detection. Slides were counterstained with DAPI, mounted and imaged as described in the PLA procedure. The Z-stack was acquired with a line averaging of 16 passages and a zoom of 2.2.

Analysis of PLA images

Proximity ligation assay images were analyzed using a script written in ImageJ macro language (http://rsbweb.nih.gov/ij/). Briefly, the script first process the Z-stack with the maximum intensity projection method, then it passes the resulting image to a Gaussian Blur filter (sigma=1), a rolling ball background subtraction algorithm (radius=10 pixels) and to a water-shedding algorithm. Finally the images where segmented using the Max Entropy algorithm (see

ImageJ documentation) and finally the PLA foci where counted using the Analyze Particle function.

SUPPLEMENTARY REFERENCES

Ashburner, M., Ball, C.A., Blake, J.A., Botstein, D., Butler, H., Cherry, J.M., Davis, A.P., Dolinski, K., Dwight, S.S., Eppig, J.T., *et al.* (2000). Gene ontology: tool for the unification of biology. The Gene Ontology Consortium. Nat Genet *25*, 25-29.

Ben-Porath, I., Thomson, M.W., Carey, V.J., Ge, R., Bell, G.W., Regev, A., and Weinberg, R.A. (2008). An embryonic stem cell-like gene expression signature in poorly differentiated aggressive human tumors. Nat Genet *40*, 499-507.

Cironi, L., Provero, P., Riggi, N., Janiszewska, M., Suva, D., Suva, M.L., Kindler, V., and Stamenkovic, I. (2009). Epigenetic features of human mesenchymal stem cells determine their permissiveness for induction of relevant transcriptional changes by SS18-SSX1. PloS one *4*, e7904.

Francis, P., Namlos, H.M., Muller, C., Eden, P., Fernebro, J., Berner, J.M., Bjerkehagen, B., Akerman, M., Bendahl, P.O., Isinger, A., *et al.* (2007). Diagnostic and prognostic gene expression signatures in 177 soft tissue sarcomas: hypoxia-induced transcription profile signifies metastatic potential. BMC genomics *8*, 73.

Gendler, K., Paulsen, T., and Napoli, C. (2008). ChromDB: the chromatin database. Nucleic Acids Res *36*, D298-302.

Irizarry, R.A., Bolstad, B.M., Collin, F., Cope, L.M., Hobbs, B., and Speed, T.P. (2003). Summaries of Affymetrix GeneChip probe level data. Nucleic Acids Res *31*, e15.

Morison, I.M., Ramsay, J.P., and Spencer, H.G. (2005). A census of mammalian imprinting. Trends Genet *21*, 457-465.

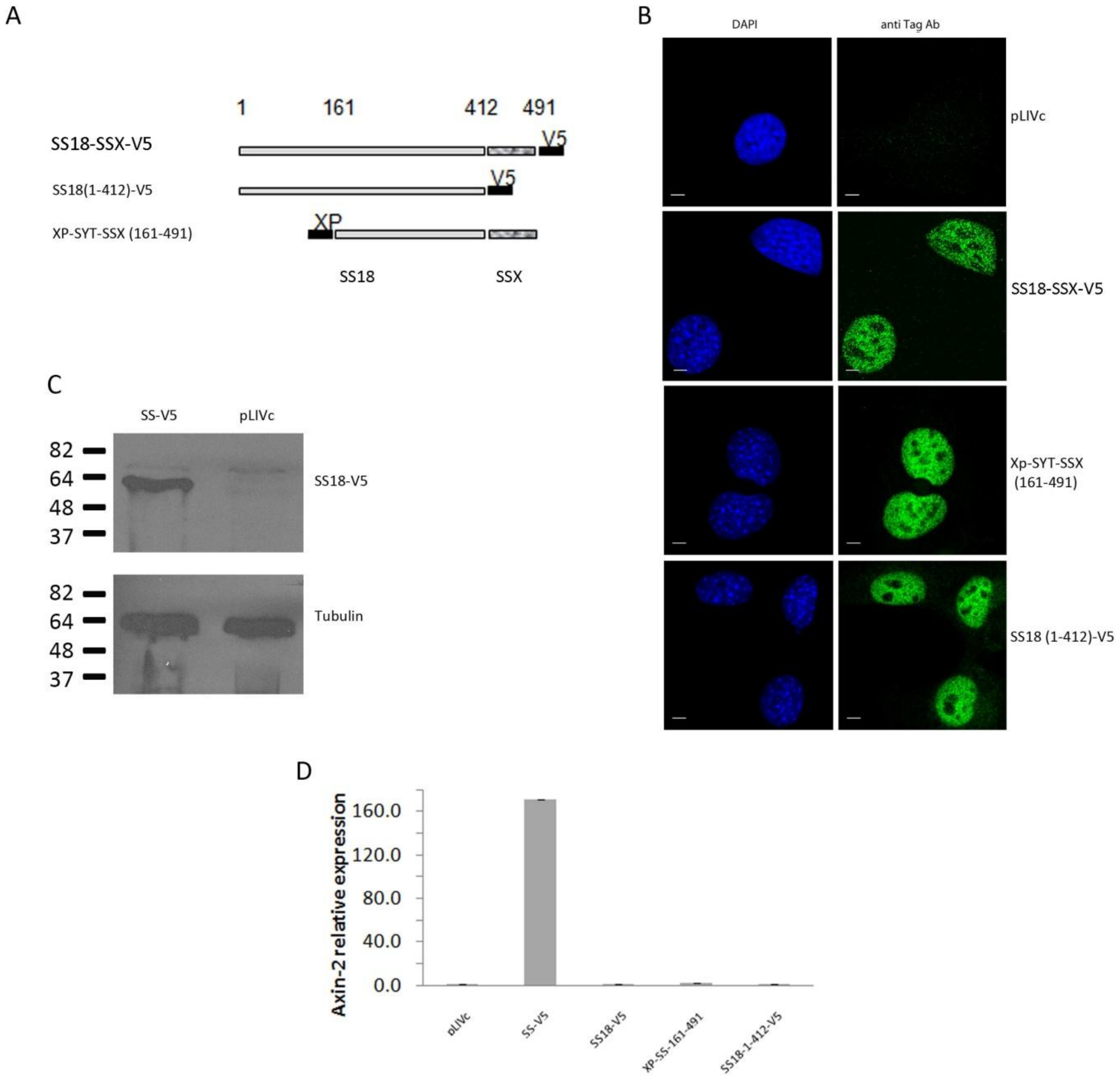

A
1
161
412
491
SS18-SSX-V5
V5
SS18(1-412)-V5
V5
XP-SYT-SSX (161-491)
XP
SS18
SSX
B
DAPI
anti Tag Ab
pLIVc
SS18-SSX-V5
Xp-SYT-SSX
(161-491)
SS18 (1-412)-V5
C
SS-V5
pLIVc
82
64
48
37
SS18-V5
82
64
48
37
Tubulin
D
Axin-2 relative expression
160.0
120.0
80.0
40.0
0.0
pLIVc
SS-V5
SS18-V5
XP-SS-161-491
SS18-1-412-V5

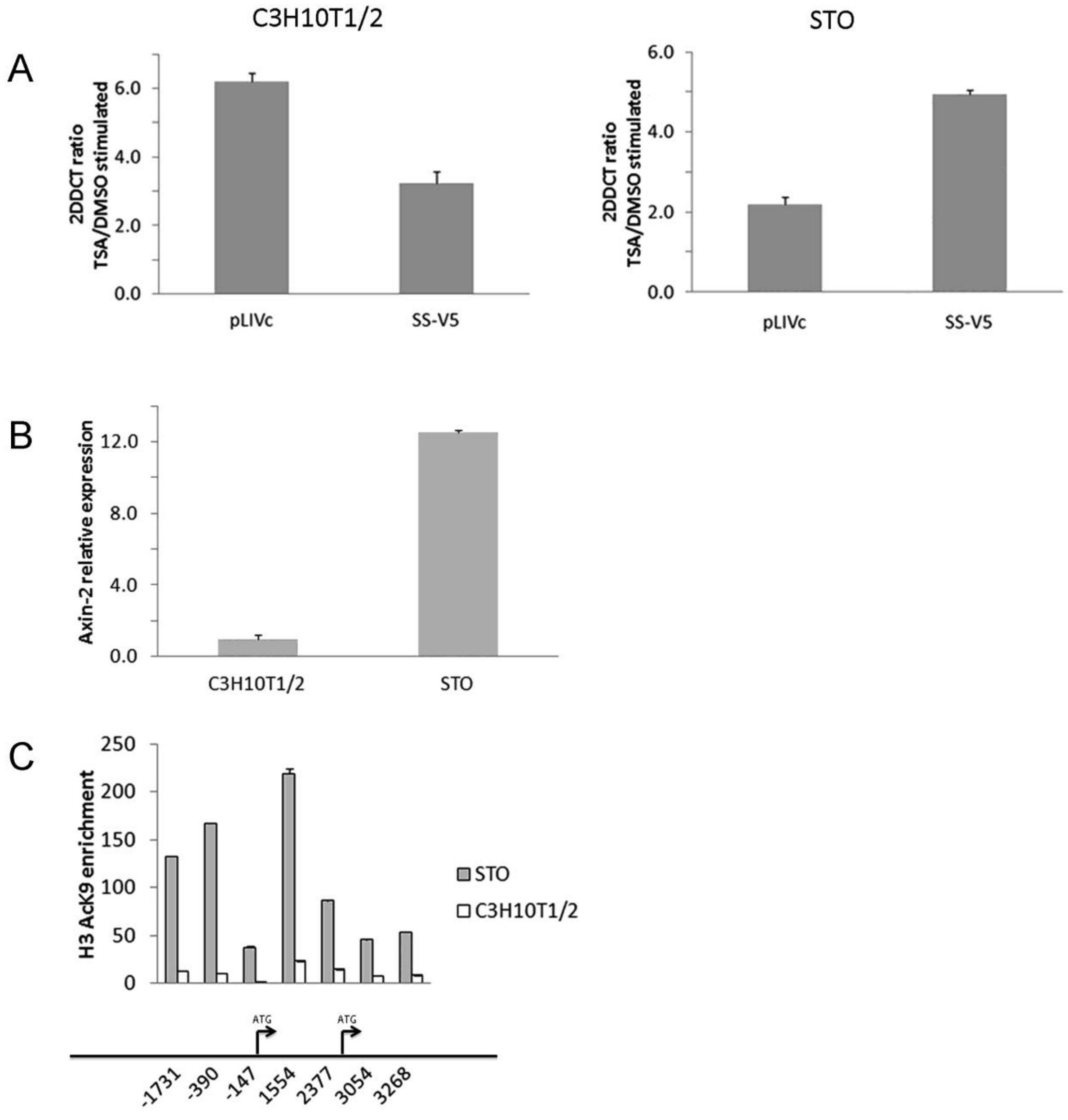
A
C3H10T1/2
2DDCT ratio TSA/DMSO stimulated
6.0
4.0
2.0
0.0
pLIVc
SS-V5
STO
2DDCT ratio TSA/DMSO stimulated
6.0
4.0
2.0
0.0
pLIVc
SS-V5
B
Axin-2 relative expression
12.0
8.0
4.0
0.0
C3H10T1/2
STO
C
H3 AcK9 enrichment
250
200
150
100
50
0
STO
C3H10T1/2
ATG
ATG
-1731
-390
-147
1554
2377
3054
3268

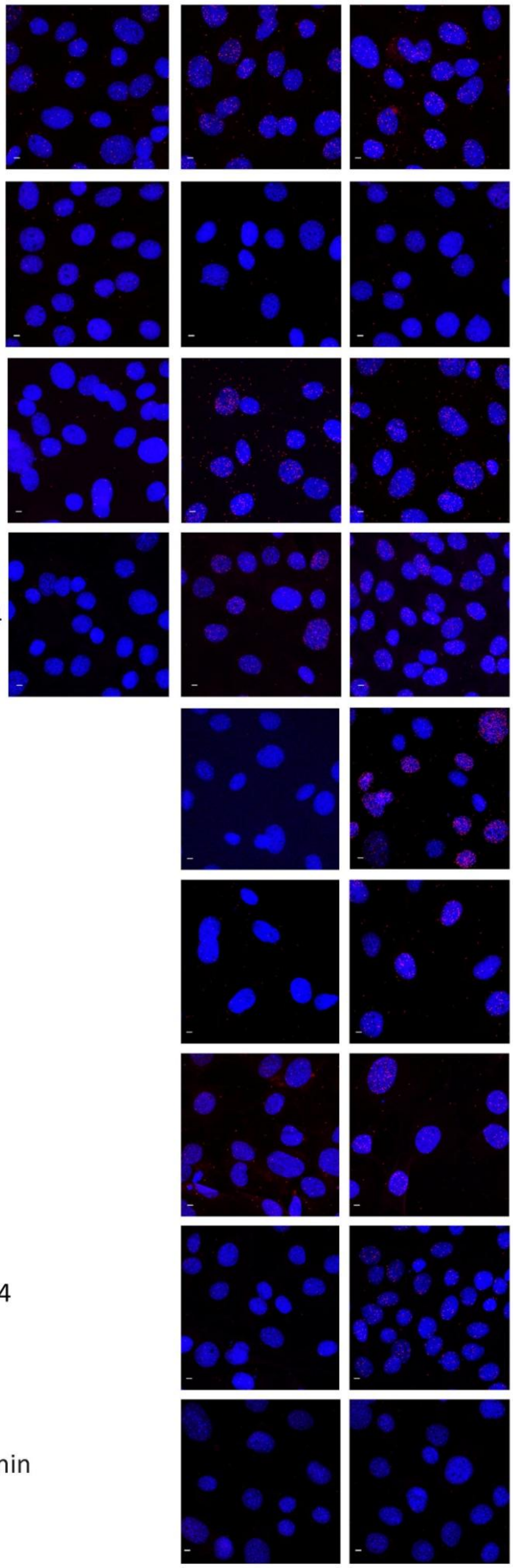
Interactions
negative control
pLIVc
SS-V5
HDAC1 and TLE
LEF-1 and TLE
HDAC1 and LEF-1
HDAC1 and TCF3/4
SS18-SSX and HDAC1
SS18-SSX and TLE
SS18-SSX and LEF-1
SS18-SSX and TCF3/4
SS18-SSX and βcatenin

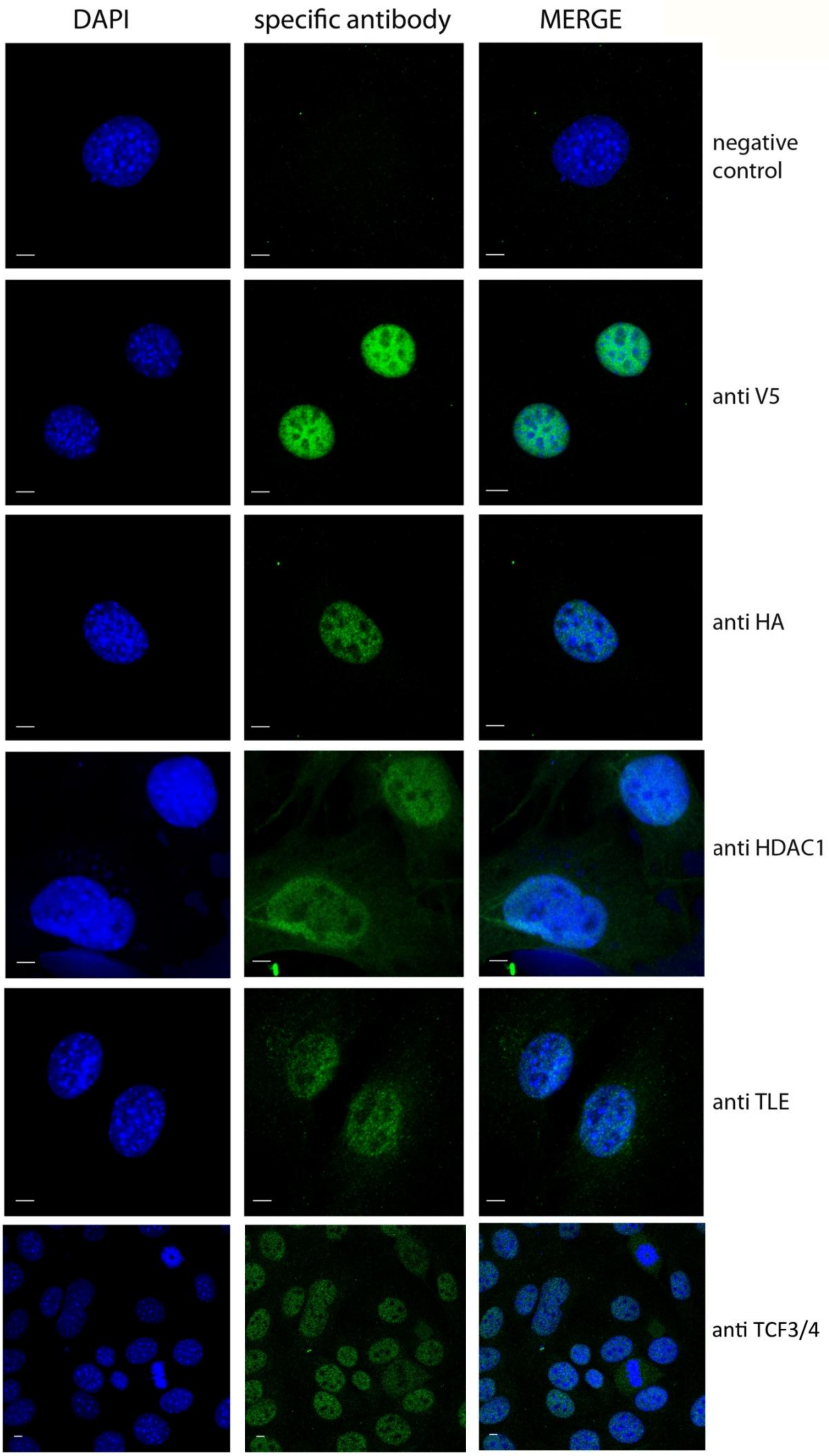

DAPI
specific antibody
MERGE
negative control
anti V5
anti HA
anti HDAC1
anti TLE
anti TCF3/4

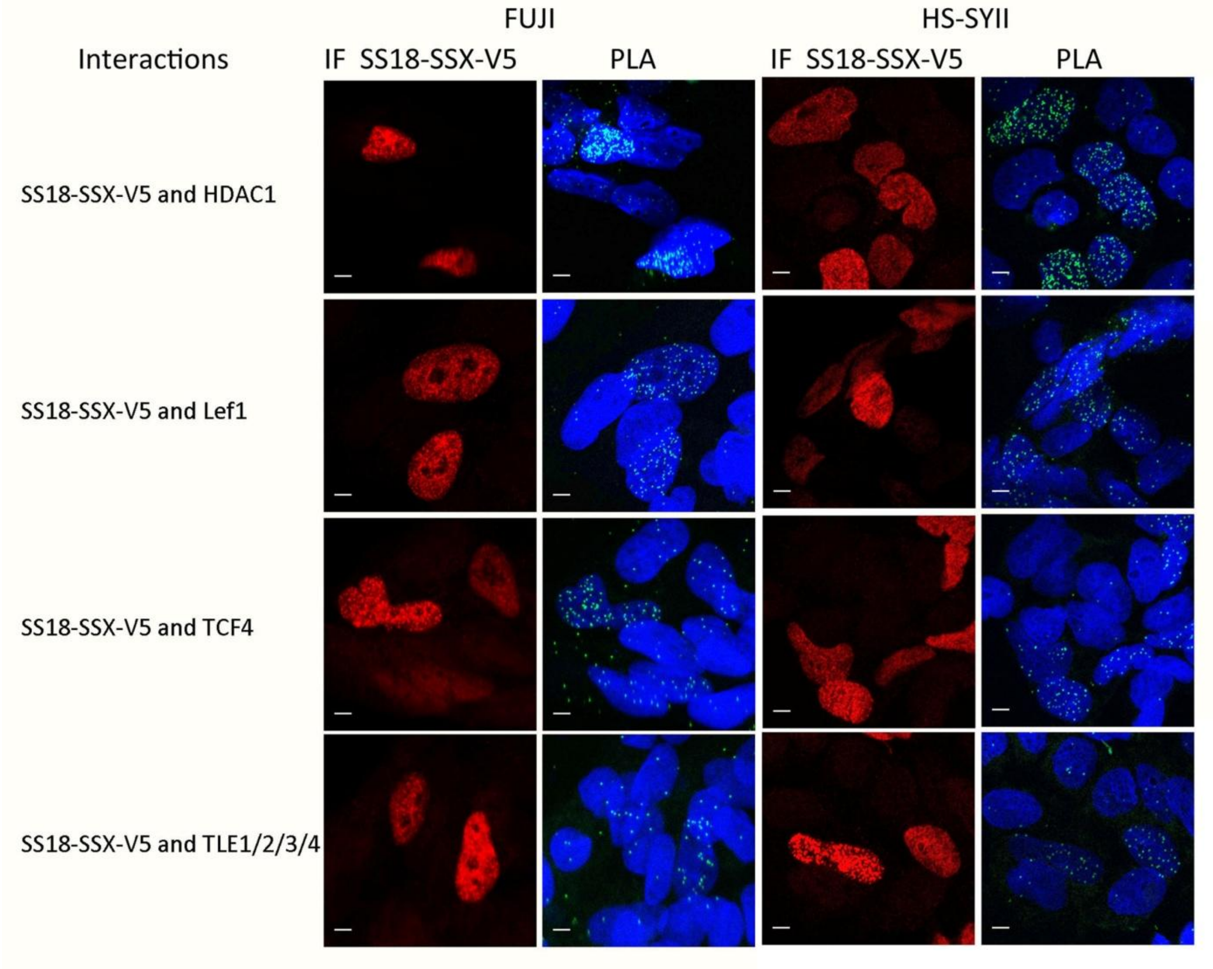
FUJI
HS-SYII
Interactions
IF SS18-SSX-V5
PLA
IF SS18-SSX-V5
PLA
SS18-SSX-V5 and HDAC1
SS18-SSX-V5 and Lef1
SS18-SSX-V5 and TCF4
SS18-SSX-V5 and TLE1/2/3/4

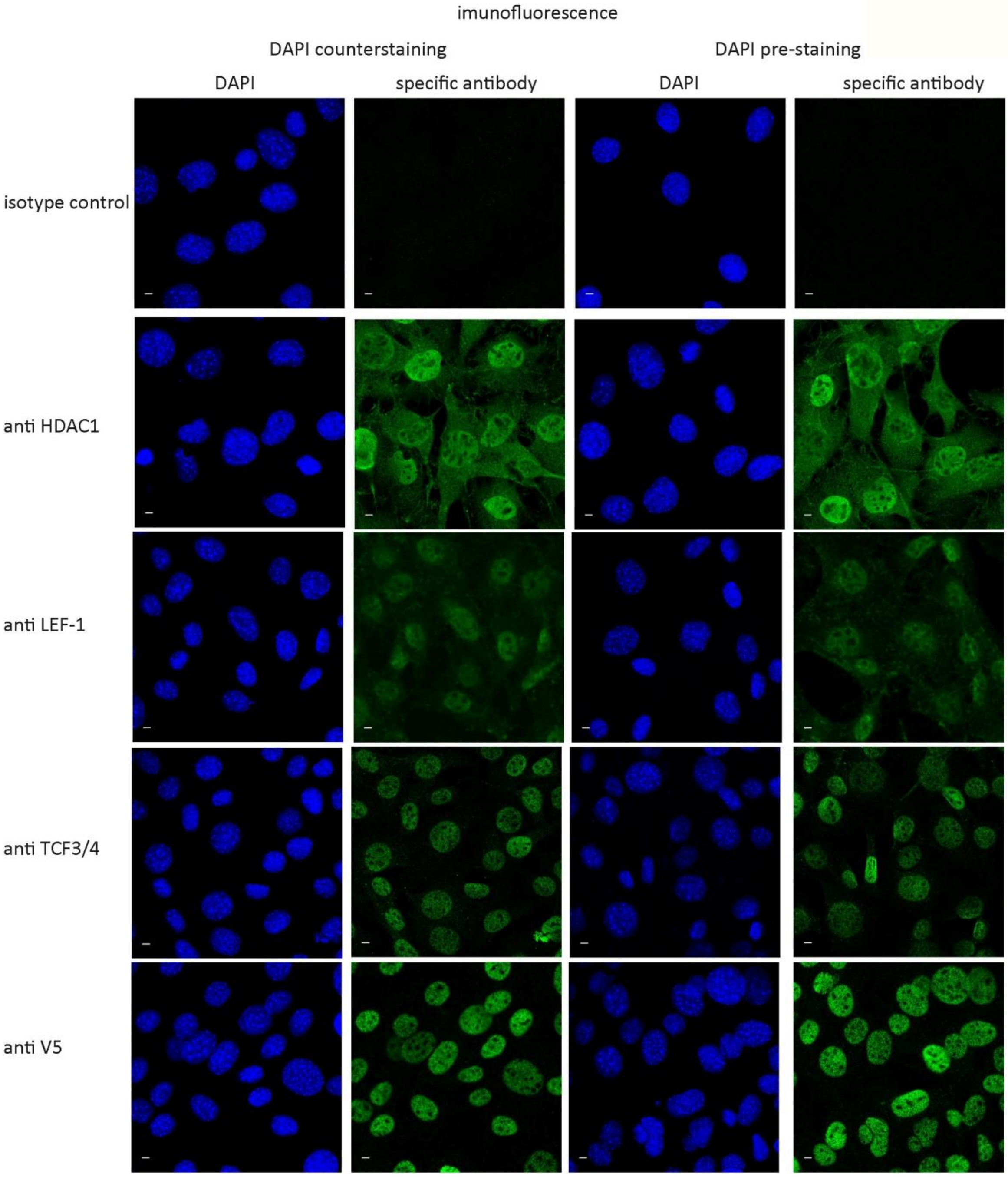
imunofluorescence
DAPI counterstaining
DAPI pre-staining
DAPI
specific antibody
DAPI
specific antibody
isotype control
anti HDAC1
anti LEF-1
anti TCF3/4
anti V5

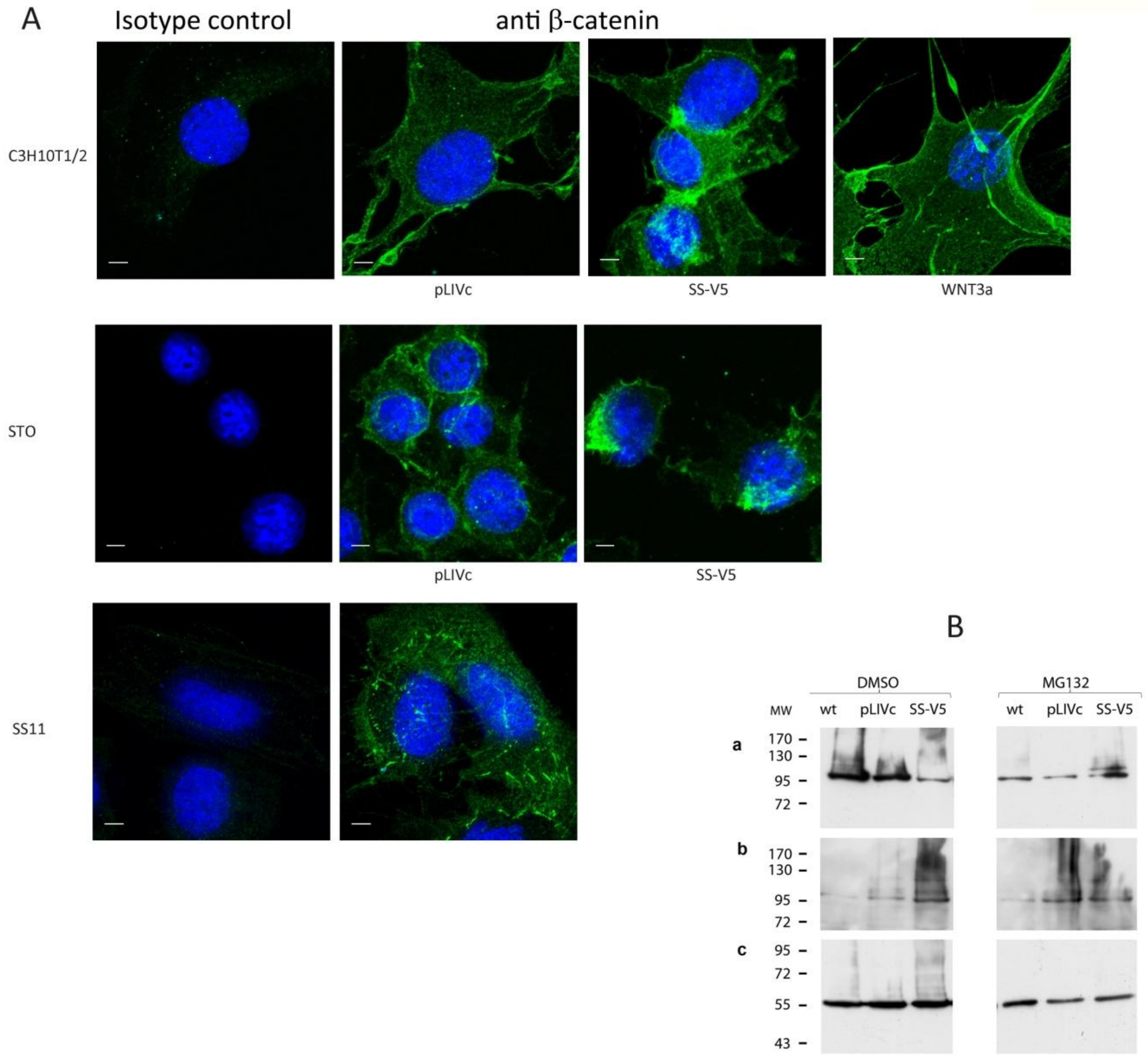

A
Isotype control
anti β-catenin
C3H10T1/2
pLIVc
SS-V5
WNT3a
STO
pLIVc
SS-V5
SS11
B
DMSO
MG132
MW
wt
pLIVc
SS-V5
wt
pLIVc
SS-V5
a
170
130
95
72
b
170
130
95
72
c
95
72
55
43

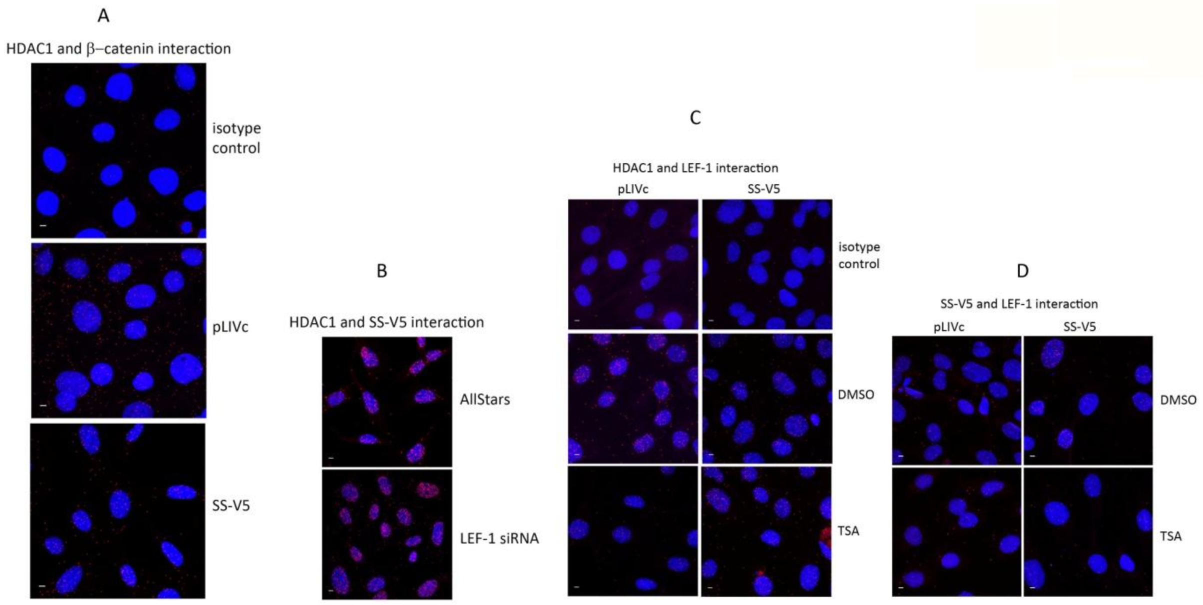

A
HDAC1 and β–catenin interaction
isotype control
pLIVc
SS-V5
B
HDAC1 and SS-V5 interaction
AllStars
LEF-1 siRNA
C
HDAC1 and LEF-1 interaction
pLIVc
SS-V5
isotype control
DMSO
TSA
D
SS-V5 and LEF-1 interaction
pLIVc
SS-V5
DMSO
TSA

Table S1. Lists of the probesets that are differentially expressed In C3H10T1/2 infected with SS18-SSX1 or an empty vector, with mean fold change > 2.

List of genes induced by SS18-SSX1 expression in C3H10T1/2

probeset	gene id	gene symbol	gene name	log FC
10490913	12350	Car3	carbonic anhydrase 3	5.57825
10349147	116872	Serpinb7	serine (or cysteine) peptidase inhibitor, clade B, member 7	4.77078
10347277	16008	Igfbp2	insulin-like growth factor binding protein 2	4.29172
10499545	13638	Efna3	ephrin A3	4.04409
10469457	67448	Plxdc2	plexin domain containing 2	3.90492
10538802	68169	A930038C07Rik	RIKEN cDNA A930038C07 gene	3.79029
10474064	277414	Trp53i11	transformation related protein 53 inducible protein 11	3.77193
10511363	18619	Penk	preproenkephalin	3.683
10372503	14160	Lgr5	leucine rich repeat containing G protein coupled receptor 5	3.62925
10474129	210622	Pamr1	peptidase domain containing associated with muscle regeneration 1	3.60038
10375175	20564	Slit3	slit homolog 3 (Drosophila)	3.57393
10380622	15417	Hoxb9	homeobox B9	3.55726
10454632	12326	Camk4	calcium/calmodulin-dependent protein kinase IV	3.51227
10389929	12291	Cacna1g	calcium channel, voltage-dependent, T type, alpha 1G subunit	3.50484
10584549	235281	Scn3b	sodium channel, voltage-gated, type III, beta	3.35744
10458999	14119	Fbn2	fibrillin 2	3.23883
10372139	67405	Nts	neurotensin	3.19329
10385036	14172	Fgf18	fibroblast growth factor 18	3.16813
10603151	14758	Gpm6b	glycoprotein m6b	3.06302
10517587	11647	Alpl	alkaline phosphatase, liver/bone/kidney	3.06103
10381096	16010	Igfbp4	insulin-like growth factor binding protein 4	3.02654
10436304	320712	Abi3bp	ABI gene family, member 3 (NESH) binding protein	3.0209
10459335	225617	-	-	2.97614
10398319	13386	Dlk1	delta-like 1 homolog (Drosophila)	2.94625
10400006	11622	Ahr	aryl-hydrocarbon receptor	2.85067
10481272	69327	1700007K13Rik	RIKEN cDNA 1700007K13 gene	2.83457
10544610	140488	Igf2bp3	insulin-like growth factor 2 mRNA binding protein 3	2.80732
10569344	16002	Igf2	insulin-like growth factor 2	2.79116
10443276	268935	Scube3	signal peptide, CUB domain, EGF-like 3	2.77638
10579894	15245	Hhip	Hedgehog-interacting protein	2.77222
10407435	105349	Akr1c18	aldo-keto reductase family 1, member C18	2.71987
10485745	228432	Ano3	anoctamin 3	2.68587
10353192	14048	Eya1	eyes absent 1 homolog (Drosophila)	2.62234
10424676	17069	Ly6e	lymphocyte antigen 6 complex, locus E	2.62013
10459353	-	-	-	2.5883
10464400	107376	E330013P04Rik	RIKEN cDNA E330013P04 gene	2.53064

10405058	27047	Omd	osteomodulin	2.53037
10338799	-	-	-	2.50527
10511429	12319	Car8	carbonic anhydrase 8	2.4992
10601848	76219	6530401D17Rik	RIKEN cDNA 6530401D17 gene	2.49538
10435948	67896	Ccdc80	coiled-coil domain containing 80	2.48428
10583809	12797	Cnn1	calponin 1	2.48047
10427272	15421	Hoxc12	homeobox C12	2.47638
10569335	14955	H19	H19 fetal liver mRNA	2.47534
10601846	-	-	-	2.46741
10380629	-	-	-	2.46304
10491721	24063	Spry1	sprouty homolog 1 (Drosophila)	2.45897
10423080	81799	C1qtnf3	C1q and tumor necrosis factor related protein 3	2.44241
10349947	14264	Fmod	fibromodulin	2.4281
10341818	-	-	-	2.40982
10549041	108096	Slco1a5	solute carrier organic anion transporter family, member 1a5	2.38574
10427268	15422	Hoxc13	homeobox C13	2.37894
10587880	76477	Pcolce2	procollagen C-endopeptidase enhancer 2	2.37678
10587616	244954	Prss35	protease, serine, 35	2.35759
10397551	-	-	-	2.35736
10339978	-	-	-	2.35453
10384223	16009	Igfbp3	insulin-like growth factor binding protein 3	2.35305
10526968	243312	Elfn1	leucine rich repeat and fibronectin type III, extracellular 1	2.33758
10553967	18553	Pcsk6	proprotein convertase subtilisin/kexin type 6	2.31955
10393662	18164	Nptx1	neuronal pentraxin 1	2.31446
10358533	545370	Hmcn1	hemicentin 1	2.29641
10459363	667742	Fam38b	family with sequence similarity 38, member B	2.27847
10499932	67718	Lce1h	late cornified envelope 1H	2.26413
10414065	11752	Anxa8	annexin A8	2.25657
10414900	-	-	-	2.2561
10414790	-	-	-	2.2561
10438730	20604	Sst	somatostatin	2.25167
10424691	17071	Ly6f	lymphocyte antigen 6 complex, locus F	2.23462
10532741	231633	Tmem119	transmembrane protein 119	2.22945
10338782	-	-	-	2.22362
10537062	17294	Mest	mesoderm specific transcript	2.21924
10558150	56213	Htra1	HtrA serine peptidase 1	2.20184
10511631	208890	Slc26a7	solute carrier family 26, member 7	2.18341
10396270	59036	Dact1	dapper homolog 1, antagonist of beta-catenin (xenopus)	2.18066
10415396	73181	Nfatc4	nuclear factor of activated T-cells, cytoplasmic, calcineurin-dependent 4	2.16905
10573779	93960	Nkd1	naked cuticle 1 homolog (Drosophila)	2.16042
10484463	12258	Serping1	serine (or cysteine) peptidase inhibitor, clade G, member 1	2.13513
10357833	381290	Atp2b4	ATPase, Ca++ transporting, plasma membrane 4	2.13238
10343914	-	-	-	2.11447
10607646	195727	Nhs	Nance-Horan syndrome (human)	2.10223

10365682	-	-	-	2.08588
10521471	269643	Ppp2r2c	protein phosphatase 2 (formerly 2A), regulatory subunit B (PR 52), gamma isoform	2.08197
10398996	68337	Crip2	cysteine rich protein 2	2.07443
10492798	20319	Sfrp2	secreted frizzled-related protein 2	2.0717
10358476	96875	Prg4	proteoglycan 4 (megakaryocyte stimulating factor, articular superficial zone protein)	2.06102
10427293	15425	Hoxc6	homeobox C6	2.05759
10507152	13118	Cyp4a12b	cytochrome P450, family 4, subfamily a, polypeptide 12B	2.04497
10358577	545370	Hmcn1	hemicentin 1	2.03212
10462442	77125	Il33	interleukin 33	2.03047
10576332	22152	Tubb3	tubulin, beta 3	2.00106
10366180	-	-	-	1.99858
10586079	319480	Itga11	integrin alpha 11	1.99837
10458828	12583	Cdo1	cysteine dioxygenase 1, cytosolic	1.99134
10409579	57266	Cxcl14	chemokine (C-X-C motif) ligand 14	1.99079
10358535	545370	Hmcn1	hemicentin 1	1.98575
10428857	211401	Mtss1	metastasis suppressor 1	1.98486
10585803	20897	Stra6	stimulated by retinoic acid gene 6	1.98344
10481518	64292	Ptges	prostaglandin E synthase	1.98125
10422059	239217	Kctd12	potassium channel tetramerisation domain containing 12	1.97924
10390175	18053	Ngfr	nerve growth factor receptor (TNFR superfamily, member 16)	1.9735
10342271	-	-	-	1.9735
10521759	20563	Slit2	slit homolog 2 (Drosophila)	1.97246
10356172	98496	Pid1	phosphotyrosine interaction domain containing 1	1.97121
10559978	-	-	-	1.96843
10478415	22403	Wisp2	WNT1 inducible signaling pathway protein 2	1.96102
10492448	19288	Ptx3	pentraxin related gene	1.9552
10577164	14456	Gas6	growth arrest specific 6	1.95186
10519886	20348	Sema3c	sema domain, immunoglobulin domain (Ig), short basic domain, secreted, (semaphorin) 3C	1.94387
10427035	15370	Nr4a1	nuclear receptor subfamily 4, group A, member 1	1.94362
10488709	329540	8430427H17Rik	RIKEN cDNA 8430427H17 gene	1.93379
10425945	14114	Fbln1	fibulin 1	1.92676
10340901	-	-	-	1.92636
10516735	94242	Tinagl1	tubulointerstitial nephritis antigen-like 1	1.92492
10424119	18133	Nov	nephroblastoma overexpressed gene	1.92295
10576049	15227	Foxf1a	forkhead box F1a	1.92019
10441093	13876	Erg	avian erythroblastosis virus E-26 (v-ets) oncogene related	1.90836
10505954	21687	Tek	endothelial-specific receptor tyrosine kinase	1.90178
10440091	12837	Col8a1	collagen, type VIII, alpha 1	1.89407
10556297	11535	Adm	adrenomedullin	1.89252
10438769	12737	Cldn1	claudin 1	1.88756
10419015	170677	Cdhr1	cadherin-related family member 1	1.88604
10358561	545370	Hmcn1	hemicentin 1	1.88316
10462035	16832	Ldhb	lactate dehydrogenase B	1.87813

10454601	53603	Tslp	thymic stromal lymphopoietin	1.87677
10523190	231440	Parm1	prostate androgen-regulated mucin-like protein 1	1.86267
10428376	11600	Angpt1	angiopoietin 1	1.86111
10443786	18585	Pde9a	phosphodiesterase 9A	1.86008
10606366	213436	Zcchc5	zinc finger, CCHC domain containing 5	1.85571
10488060	16449	Jag1	jagged 1	1.85444
10365559	16000	Igf1	insulin-like growth factor 1	1.84559
10363541	-	-	-	1.84003
10471154	-	-	-	1.83615
10342166	-	-	-	1.8343
10478048	16803	Lbp	lipopolysaccharide binding protein	1.82958
10339413	-	-	-	1.82767
10605181	19703	Renbp	renin binding protein	1.82505
10562761	20256	Clec11a	C-type lectin domain family 11, member a	1.82125
10462623	15957	Ifit1	interferon-induced protein with tetratricopeptide repeats 1	1.81952
10563812	-	-	-	1.81257
10563810	-	-	-	1.81257
10563808	668774	Gm9350	predicted gene 9350	1.81257
10563806	-	-	-	1.81257
10563804	-	-	-	1.81257
10563802	-	-	-	1.81257
10513722	326623	Tnfsf15	tumor necrosis factor (ligand) superfamily, member 15	1.80512
10391103	16480	Jup	junction plakoglobin	1.80319
10591853	57246	Tbx20	T-box 20	1.78779
10342070	-	-	-	1.78764
10361926	26408	Map3k5	mitogen-activated protein kinase kinase kinase 5	1.78707
10549972	-	-	-	1.78563
10530536	21682	Tec	tec protein tyrosine kinase	1.77261
10485198	241556	Tspan18	tetraspanin 18	1.76779
10549097	16832	Ldhb	lactate dehydrogenase B	1.76542
10341468	-	-	-	1.75907
10357965	329252	Lgr6	leucine-rich repeat-containing G protein-coupled receptor 6	1.75322
10459633	75577	2310002L13Rik	RIKEN cDNA 2310002L13 gene	1.74823
10588691	-	-	-	1.73055
10341054	-	-	-	1.72963
10344144	-	-	-	1.72486
10490923	12349	Car2	carbonic anhydrase 2	1.7244
10358633	545370	Hmcn1	hemicentin 1	1.71976
10485718	228432	Ano3	anoctamin 3	1.71807
10549964	-	-	-	1.71697
10424105	239447	Colec10	collectin sub-family member 10	1.71657
10521498	12933	Crmp1	collapsin response mediator protein 1	1.71638
10339432	-	-	-	1.71151
10355893	13838	Epha4	Eph receptor A4	1.70618
10535559	66898	Baiap2l1	BAI1-associated protein 2-like 1	1.70389

10342239	-	-	-	1.69903
10352143	269152	Kif26b	kinesin family member 26B	1.69625
10468722	14585	Gfra1	glial cell line derived neurotrophic factor family receptor alpha 1	1.69623
10405063	18295	Ogn	osteoglycin	1.68849
10459084	104027	Synpo	synaptopodin	1.68648
10338946	-	-	-	1.68529
10338786	-	-	-	1.68269
10340803	-	-	-	1.68182
10548879	17313	Mgp	matrix Gla protein	1.68126
10429515	23936	Lynx1	Ly6/neurotoxin 1	1.67884
10578572	71069	Stox2	storkhead box 2	1.66546
10363735	13654	Egr2	early growth response 2	1.66473
10340383	-	-	-	1.6632
10341435	-	-	-	1.6624
10343764	-	-	-	1.66103
10351099	240873	Tnfsf18	tumor necrosis factor (ligand) superfamily, member 18	1.64629
10417176	22772	Zic2	zinc finger protein of the cerebellum 2	1.64556
10568873	11501	Adam8	a disintegrin and metallopeptidase domain 8	1.63898
10382228	12006	Axin2	axin2	1.63864
10465619	396184	Flrt1	fibronectin leucine rich transmembrane protein 1	1.6348
10499766	26568	Slc27a3	solute carrier family 27 (fatty acid transporter), member 3	1.6337
10397346	14281	Fos	FBJ osteosarcoma oncogene	1.63193
10547740	50908	C1s	complement component 1, s subcomponent	1.63069
10341414	-	-	-	1.63068
10449258	14570	Arhgdig	Rho GDP dissociation inhibitor (GDI) gamma	1.62946
10520452	16193	Il6	interleukin 6	1.62842
10341654	-	-	-	1.62821
10344553	-	-	-	1.62449
10358527	545370	Hmcn1	hemicentin 1	1.61779
10360920	21808	Tgfb2	transforming growth factor, beta 2	1.61308
10489759	72043	Sulf2	sulfatase 2	1.61219
10431935	105827	Amigo2	adhesion molecule with Ig like domain 2	1.60802
10408629	66895	1300014I06Rik	RIKEN cDNA 1300014I06 gene	1.59456
10546450	101401	Adamts9	a disintegrin-like and metallopeptidase (reprolysin type) with thrombospondin type 1 motif, 9	1.59358
10404479	-	-	-	1.58762
10532784	68666	Svop	SV2 related protein	1.58455
10431812	54003	Nell2	NEL-like 2 (chicken)	1.58439
10343781	-	-	-	1.58426
10451372	71461	Ptk7	PTK7 protein tyrosine kinase 7	1.58367
10358583	545370	Hmcn1	hemicentin 1	1.58071
10344275	-	-	-	1.5806
10339952	-	-	-	1.57763
10489569	-	-	-	1.57378
10339691	-	-	-	1.57139

10339442	-	-	-	1.56069
10343305	-	-	-	1.55821
10447602	22350	Ezr	ezrin	1.55677
10485402	14221	Fjx1	four jointed box 1 (Drosophila)	1.54988
10464917	12794	Cnih2	cornichon homolog 2 (Drosophila)	1.54701
10373218	104080	Nxph4	neurexophilin 4	1.54598
10427286	15427	Hoxc9	homeobox C9	1.54086
10357339	-	-	-	1.53856
10344250	-	-	-	1.53719
10534343	13717	Eln	elastin	1.53506
10342279	-	-	-	1.52978
10406845	15229	Foxd1	forkhead box D1	1.52948
10342449	-	-	-	1.5278
10340493	-	-	-	1.52764
10446763	77889	Lbh	limb-bud and heart	1.52721
10399470	217410	Trib2	tribbles homolog 2 (Drosophila)	1.5269
10541678	50909	C1ra	complement component 1, r subcomponent A	1.52405
10340801	-	-	-	1.52384
10340631	-	-	-	1.52233
10541307	24110	Usp18	ubiquitin specific peptidase 18	1.51887
10578649	23965	Odz3	odd Oz/ten-m homolog 3 (Drosophila)	1.51779
10405693	69635	Dapk1	death associated protein kinase 1	1.51258
10541683	-	-	-	1.51245
10499924	68694	Lce1e	late cornified envelope 1E	1.5082
10546432	101401	Adamts9	a disintegrin-like and metallopeptidase (reprolysin type) with thrombospondin type 1 motif, 9	1.50636
10375578	14257	Flt4	FMS-like tyrosine kinase 4	1.50512
10402808	16450	Jag2	jagged 2	1.50483
10343835	-	-	-	1.50195
10339289	-	-	-	1.49969
10388254	11484	Aspa	aspartoacylase	1.49375
10404053	68024	Hist1h2bc	histone cluster 1, H2bc	1.49275
10436788	26559	Hunk	hormonally upregulated Neu-associated kinase	1.49107
10366153	237504	Rassf9	Ras association (RalGDS/AF-6) domain family (N-terminal) member 9	1.48862
10341214	-	-	-	1.48795
10576046	15227	Foxf1a	forkhead box F1a	1.4874
10448676	65962	Slc9a3r2	solute carrier family 9 (sodium/hydrogen exchanger), member 3 regulator 2	1.48137
10548996	28250	Slco1a4	solute carrier organic anion transporter family, member 1a4	1.48092
10373179	14632	Gli1	GLI-Kruppel family member GLI1	1.47555
10490129	12162	Bmp7	bone morphogenetic protein 7	1.47538
10427290	15426	Hoxc8	homeobox C8	1.4727
10342300	-	-	-	1.47124
10462281	22359	Vldlr	very low density lipoprotein receptor	1.47096
10338945	-	-	-	1.46942

10447056	70536	Qpct	glutaminyl-peptide cyclotransferase (glutaminyl cyclase)	1.46841
10338948	-	-	-	1.46526
10339995	-	-	-	1.45979
10404464	-	-	-	1.45943
10344075	-	-	-	1.45889
10539894	23945	Mgll	monoglyceride lipase	1.45773
10350733	19734	Rgs16	regulator of G-protein signaling 16	1.4564
10342610	-	-	-	1.45265
10362138	22361	Vnn1	vanin 1	1.44904
10357986	13924	Ptprv	protein tyrosine phosphatase, receptor type, V	1.44489
10546454	101401	Adamts9	a disintegrin-like and metallopeptidase (reprolysin type) with thrombospondin type 1 motif, 9	1.44144
10449608	74762	Mdga1	MAM domain containing glycosylphosphatidylinositol anchor 1	1.43945
10373918	16878	Lif	leukemia inhibitory factor	1.43706
10453178	225028	Map4k3	mitogen-activated protein kinase kinase kinase kinase 3	1.42643
10416181	20855	Stc1	stanniocalcin 1	1.42317
10458046	27528	D0H4S114	DNA segment, human D4S114	1.42191
10449775	18131	Notch3	Notch gene homolog 3 (Drosophila)	1.4217
10343174	-	-	-	1.41386
10537227	-	-	-	1.40929
10361771	22634	Plagl1	pleiomorphic adenoma gene-like 1	1.40783
10536294	170676	Peg10	paternally expressed 10	1.40707
10571467	68797	Pdgfrl	platelet-derived growth factor receptor-like	1.40507
10339863	-	-	-	1.40471
10399973	79221	Hdac9	histone deacetylase 9	1.40407
10422822	16880	Lifr	leukemia inhibitory factor receptor	1.39872
10384233	319939	Tns3	tensin 3	1.39831
10585214	-	-	-	1.39815
10339059	-	-	-	1.39242
10401831	435337	Gm5662	predicted gene 5662	1.39063
10341955	-	-	-	1.3896
10535174	231832	Tmem184a	transmembrane protein 184a	1.38679
10370798	66548	Adamtsl5	ADAMTS-like 5	1.38466
10339842	-	-	-	1.38321
10594044	26968	Islr	immunoglobulin superfamily containing leucine-rich repeat	1.38232
10490384	16776	Lama5	laminin, alpha 5	1.38119
10343514	-	-	-	1.37943
10344952	98711	Rdh10	retinol dehydrogenase 10 (all-trans)	1.3792
10338884	-	-	-	1.37545
10346843	18187	Nrp2	neuropilin 2	1.37317
10343119	-	-	-	1.37128
10342856	-	-	-	1.36911
10351623	16456	F11r	F11 receptor	1.36764
10342367	-	-	-	1.36735
10338455	-	-	-	1.35841

10343972	-	-	-	1.35799
10540523	30937	Lmcd1	LIM and cysteine-rich domains 1	1.35788
10555174	434215	Lrrc32	leucine rich repeat containing 32	1.35747
10416023	71145	Scara5	scavenger receptor class A, member 5 (putative)	1.35646
10343327	-	-	-	1.35535
10401673	21809	Tgfb3	transforming growth factor, beta 3	1.355
10584165	67703	Kirrel3	kin of IRRE like 3 (Drosophila)	1.35424
10564624	20450	St8sia2	ST8 alpha-N-acetyl-neuraminide alpha-2,8-sialyltransferase 2	1.34637
10338643	-	-	-	1.34499
10359571	14261	Fmo1	flavin containing monooxygenase 1	1.343
10566583	668139	Gm8995	predicted gene 8995	1.34277
10410547	72293	Nkd2	naked cuticle 2 homolog (Drosophila)	1.34232
10508614	14077	Fabp3	fatty acid binding protein 3, muscle and heart	1.34222
10463476	107250	Kazald1	Kazal-type serine peptidase inhibitor domain 1	1.34161
10341382	-	-	-	1.34005
10551185	21803	Tgfb1	transforming growth factor, beta 1	1.33974
10499952	74175	Crct1	cysteine-rich C-terminal 1	1.33808
10492355	17380	Mme	membrane metallo endopeptidase	1.33598
10343825	-	-	-	1.33535
10410039	19206	Ptch1	patched homolog 1	1.33435
10343982	-	-	-	1.33309
10342998	-	-	-	1.33248
10361887	64058	Perp	PERP, TP53 apoptosis effector	1.33089
10512279	12804	Cntfr	ciliary neurotrophic factor receptor	1.32978
10420362	-	-	-	1.32874
10405587	21810	Tgfbi	transforming growth factor, beta induced	1.32778
10344571	-	-	-	1.32698
10467766	67573	Loxl4	lysyl oxidase-like 4	1.32265
10521796	-	-	-	1.31867
10351455	19737	Rgs5	regulator of G-protein signaling 5	1.31646
10398412	-	-	-	1.31641
10540472	20893	Bhlhe40	basic helix-loop-helix family, member e40	1.3154
10476740	94249	Slc24a3	solute carrier family 24 (sodium/potassium/calcium exchanger), member 3	1.31408
10339951	-	-	-	1.31038
10344566	-	-	-	1.31018
10512022	214944	Mobkl2b	MOB1, Mps One Binder kinase activator-like 2B (yeast)	1.30704
10470529	56177	Olfm1	olfactomedin 1	1.30317
10385770	257871	Olfr1372-ps1	olfactory receptor 1372, pseudogene 1	1.30142
10355984	20720	Serpine2	serine (or cysteine) peptidase inhibitor, clade E, member 2	1.3014
10365974	13179	Dcn	decorin	1.30046
10460541	70445	Cd248	CD248 antigen, endosialin	1.29905
10514347	12579	Cdkn2b	cyclin-dependent kinase inhibitor 2B (p15, inhibits CDK4)	1.29754
10342527	-	-	-	1.29275
10512949	11303	Abca1	ATP-binding cassette, sub-family A (ABC1), member 1	1.29255

10344981	94227	Pi15	peptidase inhibitor 15	1.29225
10528227	14677	Gnai1	guanine nucleotide binding protein (G protein), alpha inhibiting 1	1.29076
10405781	-	-	-	1.29062
10409464	56320	Dbn1	drebrin 1	1.29041
10549420	387314	Tmtc1	transmembrane and tetratricopeptide repeat containing 1	1.28882
10344453	-	-	-	1.28809
10357875	12227	Btg2	B-cell translocation gene 2, anti-proliferative	1.2878
10434925	15205	Hes1	hairy and enhancer of split 1 (Drosophila)	1.2857
10414025	14560	Gdf10	growth differentiation factor 10	1.28355
10339310	-	-	-	1.28271
10404472	-	-	-	1.28202
10424686	223631	BC025446	cDNA sequence BC025446	1.28185
10413482	22418	Wnt5a	wingless-related MMTV integration site 5A	1.2806
10502081	13809	Enpep	glutamyl aminopeptidase	1.27935
10433445	268860	Abat	4-aminobutyrate aminotransferase	1.27519
10405047	66695	Aspn	asporin	1.27207
10513008	16600	Klf4	Kruppel-like factor 4 (gut)	1.27197
10435961	100038470	Gm10808	predicted gene 10808	1.27102
10366266	114774	Pawr	PRKC, apoptosis, WT1, regulator	1.26803
10341019	-	-	-	1.26633
10496373	73284	Ddit4l	DNA-damage-inducible transcript 4-like	1.25999
10548030	12527	Cd9	CD9 antigen	1.25882
10494003	399674	Tdpoz3	TD and POZ domain containing 3	1.25727
10358549	545370	Hmcn1	hemicentin 1	1.25644
10343185	-	-	-	1.25639
10402473	94040	Clmn	calmin	1.25463
10536025	-	-	-	1.25131
10523337	-	-	-	1.25131
10342888	-	-	-	1.24935
10343694	-	-	-	1.24254
10505914	236537	Zfp352	zinc finger protein 352	1.24166
10374777	216616	Efemp1	epidermal growth factor-containing fibulin-like extracellular matrix protein 1	1.23749
10599174	16164	Il13ra1	interleukin 13 receptor, alpha 1	1.2371
10339159	-	-	-	1.23674
10339266	-	-	-	1.23527
10385500	15944	Irgm1	immunity-related GTPase family M member 1	1.23281
10344110	-	-	-	1.23169
10339417	-	-	-	1.23131
10521111	14184	Fgfr3	fibroblast growth factor receptor 3	1.23013
10485378	72446	Prr5l	proline rich 5 like	1.22759
10419261	12159	Bmp4	bone morphogenetic protein 4	1.22727
10418193	211623	Plac9	placenta specific 9	1.22691
10339420	-	-	-	1.22596
10407350	14165	Fgf10	fibroblast growth factor 10	1.2257

10458843	20358	Sema6a	sema domain, transmembrane domain (TM), and cytoplasmic domain, (semaphorin) 6A	1.22221
10338589	-	-	-	1.2172
10598626	21912	Tspan7	tetraspanin 7	1.21708
10341517	-	-	-	1.21577
10344178	-	-	-	1.2154
10347491	22420	Wnt6	wingless-related MMTV integration site 6	1.2151
10493449	21827	Thbs3	thrombospondin 3	1.21424
10338426	-	-	-	1.21352
10338874	-	-	-	1.20771
10536143	624931	LOC624931	similar to D5Ertd577e protein	1.20613
10536052	-	-	-	1.20613
10438753	210530	Leprel1	leprecan-like 1	1.20542
10405033	407800	Ecm2	extracellular matrix protein 2, female organ and adipocyte specific	1.20523
10351463	19737	Rgs5	regulator of G-protein signaling 5	1.20186
10342492	-	-	-	1.20007
10343472	-	-	-	1.1957
10404783	13614	Edn1	endothelin 1	1.19524
10425880	109270	Prr5	proline rich 5 (renal)	1.19397
10415319	16391	Irf9	interferon regulatory factor 9	1.19397
10493108	12904	Crabp2	cellular retinoic acid binding protein II	1.19281
10342685	-	-	-	1.19275
10344261	-	-	-	1.19248
10514939	242608	Podn	podocan	1.18752
10418205	211623	Plac9	placenta specific 9	1.1872
10418180	211623	Plac9	placenta specific 9	1.1872
10404458	20710	Serpinb9e	serine (or cysteine) peptidase inhibitor, clade B, member 9e	1.18714
10430660	18591	Pdgfb	platelet derived growth factor, B polypeptide	1.18409
10423293	17909	Myo10	myosin X	1.18333
10341183	-	-	-	1.18233
10338113	-	-	-	1.18076
10527936	14362	Fzd1	frizzled homolog 1 (Drosophila)	1.18047
10479397	18216	Ntsr1	neurotensin receptor 1	1.17998
10340061	-	-	-	1.17943
10597239	19228	Pth1r	parathyroid hormone 1 receptor	1.17789
10364149	20203	S100b	S100 protein, beta polypeptide, neural	1.17717
10400143	217517	Stxbp6	syntaxin binding protein 6 (amisyn)	1.17253
10338744	-	-	-	1.17019
10562251	20266	Scn1b	sodium channel, voltage-gated, type I, beta	1.16973
10569102	54123	Irf7	interferon regulatory factor 7	1.16952
10340935	-	-	-	1.16874
10566358	20128	Trim30	tripartite motif-containing 30	1.16865
10342245	-	-	-	1.16787
10522467	68939	Rasl11b	RAS-like, family 11, member B	1.16532
10343257	-	-	-	1.15993

10344487	-	-	-	1.15824
10571444	11988	Slc7a2	solute carrier family 7 (cationic amino acid transporter, y+ system), member 2	1.1577
10419578	29811	Ndrg2	N-myc downstream regulated gene 2	1.15579
10342923	-	-	-	1.15532
10342223	-	-	-	1.15377
10338425	-	-	-	1.1528
10420957	19229	Ptk2b	PTK2 protein tyrosine kinase 2 beta	1.15233
10565315	14085	Fah	fumarylacetoacetate hydrolase	1.15093
10339832	-	-	-	1.15045
10356084	16367	Irs1	insulin receptor substrate 1	1.14958
10500808	99543	Olfml3	olfactomedin-like 3	1.14897
10393926	67880	Dcxr	dicarbonyl L-xylulose reductase	1.14862
10343481	-	-	-	1.14718
10398039	-	-	-	1.14632
10340718	-	-	-	1.14437
10461057	104383	Rcor2	REST corepressor 2	1.14326
10343277	-	-	-	1.14316
10493798	67860	S100a16	S100 calcium binding protein A16	1.14128
10389752	18121	Nog	noggin	1.13904
10341796	-	-	-	1.1384
10576386	69581	Rhou	ras homolog gene family, member U	1.13661
10351111	474332	Dnm3os	dynamin 3, opposite strand	1.13464
10344197	-	-	-	1.13367
10358670	545370	Hmcn1	hemicentin 1	1.1336
10343436	-	-	-	1.13353
10363161	-	-	-	1.13321
10455761	225518	Prdm6	PR domain containing 6	1.12647
10566205	384701	Dub2a	deubiquitinating enzyme 2a	1.12449
10343558	-	-	-	1.12428
10366653	24117	Wif1	Wnt inhibitory factor 1	1.12419
10568553	77590	Chst15	carbohydrate (N-acetylgalactosamine 4-sulfate 6-O) sulfotransferase 15	1.12335
10339433	-	-	-	1.12272
10338698	-	-	-	1.12118
10358581	545370	Hmcn1	hemicentin 1	1.12096
10562408	68947	Chst8	carbohydrate (N-acetylgalactosamine 4-0) sulfotransferase 8	1.12058
10343301	-	-	-	1.1203
10368495	72780	Rspo3	R-spondin 3 homolog (Xenopus laevis)	1.12023
10550574	13400	Dmpk	dystrophia myotonica-protein kinase	1.11948
10572398	12931	Crlf1	cytokine receptor-like factor 1	1.11809
10415857	219148	Fam167a	family with sequence similarity 167, member A	1.11761
10358529	545370	Hmcn1	hemicentin 1	1.11612
10439612	117606	Boc	biregional cell adhesion molecule-related/down-regulated by oncogenes (Cdon) binding protein	1.11414
10416800	380928	Lmo7	LIM domain only 7	1.11374

10487040	-	-	-	1.11284
10586306	56741	Igdcc4	immunoglobulin superfamily, DCC subclass, member 4	1.11199
10569494	79202	Tnfrsf22	tumor necrosis factor receptor superfamily, member 22	1.11175
10338807	-	-	-	1.11175
10508012	192199	Rspo1	R-spondin homolog (Xenopus laevis)	1.11023
10395466	238130	Dock4	dedicator of cytokinesis 4	1.10906
10403743	16323	Inhba	inhibin beta-A	1.10895
10372831	74694	Tbc1d30	TBC1 domain family, member 30	1.10862
10344121	-	-	-	1.10624
10371502	14077	Fabp3	fatty acid binding protein 3, muscle and heart	1.1059
10538658	73998	Herc3	hect domain and RLD 3	1.10589
10492136	13175	Dclk1	doublecortin-like kinase 1	1.10347
10427744	75646	Rai14	retinoic acid induced 14	1.1005
10339571	-	-	-	1.09633
10341785	-	-	-	1.09584
10493555	140493	Kcnn3	potassium intermediate/small conductance calcium-activated channel, subfamily N, member 3	1.09569
10589654	235633	Als2cl	ALS2 C-terminal like	1.09536
10471247	108897	Aif1l	allograft inflammatory factor 1-like	1.09469
10341440	-	-	-	1.09133
10405779	-	-	-	1.08818
10579812	13617	Ednra	endothelin receptor type A	1.08476
10372891	117600	Srgap1	SLIT-ROBO Rho GTPase activating protein 1	1.08259
10364784	70335	Reep6	receptor accessory protein 6	1.07976
10356712	16560	Kif1a	kinesin family member 1A	1.07704
10365482	21859	Timp3	tissue inhibitor of metalloproteinase 3	1.07472
10343723	-	-	-	1.0746
10548038	18205	Ntf3	neurotrophin 3	1.07395
10455721	67847	Sncaip	synuclein, alpha interacting protein (synphilin)	1.07299
10566767	76954	St5	suppression of tumorigenicity 5	1.06863
10344328	-	-	-	1.06699
10342561	-	-	-	1.0653
10569341	14955	H19	H19 fetal liver mRNA	1.06527
10339881	-	-	-	1.06465
10343062	-	-	-	1.06429
10339771	-	-	-	1.06397
10439009	11815	Apod	apolipoprotein D	1.06319
10340083	-	-	-	1.06272
10398326	17263	Meg3	maternally expressed 3	1.06133
10559509	58804	Cdc42ep5	CDC42 effector protein (Rho GTPase binding) 5	1.06126
10415411	277154	Nynrin	NYN domain and retroviral integrase containing	1.06088
10341411	-	-	-	1.06087
10569017	66141	Ifitm3	interferon induced transmembrane protein 3	1.05931
10343057	-	-	-	1.05891
10342796	-	-	-	1.05876

10340722	-	-	-	1.05793
10529636	64293	Stk32b	serine/threonine kinase 32B	1.0564
10343125	-	-	-	1.05495
10344375	-	-	-	1.05421
10342364	-	-	-	1.0526
10342966	-	-	-	1.05258
10488147	71436	Flrt3	fibronectin leucine rich transmembrane protein 3	1.05168
10348451	12778	Cxcr7	chemokine (C-X-C motif) receptor 7	1.05139
10440344	268902	Robo2	roundabout homolog 2 (Drosophila)	1.05072
10521537	231162	Cytl1	cytokine-like 1	1.05042
10357870	116847	Prelp	proline arginine-rich end leucine-rich repeat	1.0502
10522558	-	-	-	1.05002
10343422	-	-	-	1.04959
10481056	18128	Notch1	Notch gene homolog 1 (Drosophila)	1.04783
10347398	-	-	-	1.04747
10358525	545370	Hmcn1	hemicentin 1	1.04489
10341960	-	-	-	1.04427
10366546	70574	Cpm	carboxypeptidase M	1.04327
10405783	-	-	-	1.04309
10457587	225207	Zfp521	zinc finger protein 521	1.04131
10462507	23972	Papss2	3'-phosphoadenosine 5'-phosphosulfate synthase 2	1.04089
10339120	-	-	-	1.04019
10343358	-	-	-	1.04016
10372028	54712	Plxnc1	plexin C1	1.03985
10578222	-	-	-	1.03978
10344401	-	-	-	1.0397
10415408	277154	Nynrin	NYN domain and retroviral integrase containing	1.03823
10341441	-	-	-	1.03552
10448925	58226	Cacna1h	calcium channel, voltage-dependent, T type, alpha 1H subunit	1.0353
10338405	-	-	-	1.03432
10344123	-	-	-	1.03402
10343886	-	-	-	1.03377
10378549	237847	Rtn4rl1	reticulon 4 receptor-like 1	1.03345
10358660	545370	Hmcn1	hemicentin 1	1.03313
10339529	-	-	-	1.03188
10369702	52463	Tet1	tet oncogene 1	1.03072
10569504	79201	Tnfrsf23	tumor necrosis factor receptor superfamily, member 23	1.03044
10524312	209683	Ttc28	tetratricopeptide repeat domain 28	1.0282
10507099	666048	Gm12824	predicted gene 12824	1.02766
10398420	-	-	-	1.02725
10546430	101401	Adamts9	a disintegrin-like and metallopeptidase (reprolysin type) with thrombospondin type 1 motif, 9	1.02688
10503259	60599	Trp53inp1	transformation related protein 53 inducible nuclear protein 1	1.02682
10604961	14396	Gabra3	gamma-aminobutyric acid (GABA) A receptor, subunit alpha 3	1.02561

10395908	-	-	-	1.02533
10474361	77015	Mpped2	metallophosphoesterase domain containing 2	1.02526
10540408	16438	Itpr1	inositol 1,4,5-triphosphate receptor 1	1.02466
10338696	-	-	-	1.02271
10459905	240427	Setbp1	SET binding protein 1	1.02115
10466886	-	-	-	1.02091
10367400	58223	Mmp19	matrix metallopeptidase 19	1.01917
10369690	52463	Tet1	tet oncogene 1	1.01858
10338562	-	-	-	1.01805
10429140	17988	Ndrg1	N-myc downstream regulated gene 1	1.0176
10580577	16373	Irx3	Iroquois related homeobox 3 (Drosophila)	1.01743
10412921	18074	Nid2	nidogen 2	1.0163
10342727	-	-	-	1.01508
10454514	-	-	-	1.01485
10339663	-	-	-	1.01436
10339223	-	-	-	1.01391
10574545	234673	Ces5	carboxylesterase 5	1.0136
10343056	-	-	-	1.01301
10374366	13649	Egfr	epidermal growth factor receptor	1.01162
10427471	18414	Osmr	oncostatin M receptor	1.0115
10404996	18081	Ninj1	ninjurin 1	1.0111
10524310	209683	Ttc28	tetratricopeptide repeat domain 28	1.01078
10340141	-	-	-	1.01066
10574259	14766	Gpr56	G protein-coupled receptor 56	1.00995
10343688	-	-	-	1.00968
10340407	-	-	-	1.00828
10342063	-	-	-	1.00824
10424683	546644	Ly6g	lymphocyte antigen 6 complex, locus G	1.00597
10341946	-	-	-	1.00557
10340997	-	-	-	1.00531
10361358	56533	Rgs17	regulator of G-protein signaling 17	1.00308
10413171	-	-	-	1.00044

List of genes repressed by SS18-SSX1 expression in C3H10T1/2

probeset	gene id	gene symbol	gene name	logFC
10403579	107849	Prl2c5	prolactin family 2, subfamily c, member 5	-3.79667
10407797	-	-	-	-3.78516
10367582	22353	Vip	vasoactive intestinal polypeptide	-3.44102

10406504	13612	Edil3	EGF-like repeats and discoidin I-like domains 3	-2.52187
10538482	11517	Adcyap1r1	adenylate cyclase activating polypeptide 1 receptor 1	-2.52106
10341932	-	-	-	-2.45341
10342476	-	-	-	-2.42986
10519857	15234	Hgf	hepatocyte growth factor	-2.41857
10547657	12267	C3ar1	complement component 3a receptor 1	-2.38806
10368999	14806	Grik2	glutamate receptor, ionotropic, kainate 2 (beta 2)	-2.17592
10367591	68632	Myct1	myc target 1	-2.13993
10572070	18166	Npy1r	neuropeptide Y receptor Y1	-2.126
10480003	16425	Itih2	inter-alpha trypsin inhibitor, heavy chain 2	-2.11725
10502230	-	-	-	-2.09178
10494043	72634	Tdrkh	tudor and KH domain containing protein	-1.96261
10436666	67374	Jam2	junction adhesion molecule 2	-1.94722
10406982	108154	Adamts6	a disintegrin-like and metallopeptidase (reprolysin type) with thrombospondin type 1 motif, 6	-1.94086
10579012	234356	Csgalnact1	chondroitin sulfate N-acetylgalactosaminyltransferase 1	-1.82749
10344135	-	-	-	-1.77193
10340985	-	-	-	-1.76239
10340018	-	-	-	-1.73756
10343791	-	-	-	-1.70673
10341524	-	-	-	-1.70373
10395155	104943	Fam110c	family with sequence similarity 110, member C	-1.68948
10556302	11717	Ampd3	adenosine monophosphate deaminase 3	-1.66368
10343148	-	-	-	-1.66118
10340650	-	-	-	-1.65625
10426581	239650	AI836003	expressed sequence AI836003	-1.56047
10342497	-	-	-	-1.55956
10491860	269424	Phf17	PHD finger protein 17	-1.55639
10341668	-	-	-	-1.54668
10467191	107765	Ankrd1	ankyrin repeat domain 1 (cardiac muscle)	-1.54296
10554863	83671	Sytl2	synaptotagmin-like 2	-1.53443
10543120	15893	Ica1	islet cell autoantigen 1	-1.52133
10360398	26388	Ifi202b	interferon activated gene 202B	-1.51686
10341974	-	-	-	-1.50212
10341635	-	-	-	-1.49174
10344222	-	-	-	-1.48841
10404422	20708	Serpinb6b	serine (or cysteine) peptidase inhibitor, clade B, member 6b	-1.4762
10386076	14654	Glra1	glycine receptor, alpha 1 subunit	-1.47147
10578880	21892	Tll1	tolloid-like	-1.46388
10339108	-	-	-	-1.44103
10353102	329093	Cpa6	carboxypeptidase A6	-1.42994
10459496	320924	Ccbe1	collagen and calcium binding EGF domains 1	-1.42068
10354432	17912	Myo1b	myosin IB	-1.41668
10601993	102871	D330045A20Rik	RIKEN cDNA D330045A20 gene	-1.40742
10458382	12475	Cd14	CD14 antigen	-1.386

10341915	-	-	-	-1.38516
10351477	26904	Sh2d1b1	SH2 domain protein 1B1	-1.37498
10447732	69310	Pacrg	PARK2 co-regulated	-1.37398
10339720	-	-	-	-1.37349
10341562	-	-	-	-1.36401
10602009	66889	Rnf128	ring finger protein 128	-1.36398
10343879	-	-	-	-1.35077
10375402	11492	Adam19	a disintegrin and metallopeptidase domain 19 (meltrin beta)	-1.34576
10338250	-	-	-	-1.34551
10367982	215798	Gpr126	G protein-coupled receptor 126	-1.34311
10339788	-	-	-	-1.34265
10344501	-	-	-	-1.34056
10343493	-	-	-	-1.33367
10544440	-	-	-	-1.32506
10340560	-	-	-	-1.32454
10545065	243385	Gprin3	GPRIN family member 3	-1.3236
10340974	-	-	-	-1.32316
10342752	-	-	-	-1.32139
10338502	-	-	-	-1.31938
10499941	433619	Kprp	keratinocyte expressed, proline-rich	-1.3169
10423333	66270	Fam134b	family with sequence similarity 134, member B	-1.31599
10338069	-	-	-	-1.31311
10344424	-	-	-	-1.31054
10599562	75404	1100001E04Rik	RIKEN cDNA 1100001E04 gene	-1.30805
10433887	67451	Pkp2	plakophilin 2	-1.30022
10344523	-	-	-	-1.29193
10338775	-	-	-	-1.286
10338267	-	-	-	-1.28024
10338097	-	-	-	-1.27714
10339338	-	-	-	-1.26737
10343012	-	-	-	-1.25777
10344245	-	-	-	-1.25669
10343929	-	-	-	-1.25625
10351206	20344	Selp	selectin, platelet	-1.2538
10339470	-	-	-	-1.25346
10539517	26903	Dysf	dysferlin	-1.25169
10339819	-	-	-	-1.24719
10471721	19224	Ptgs1	prostaglandin-endoperoxide synthase 1	-1.24663
10340015	-	-	-	-1.24551
10406823	-	-	-	-1.24407
10342972	-	-	-	-1.23906
10342694	-	-	-	-1.23642
10341001	-	-	-	-1.23037
10340207	-	-	-	-1.22726
10344033	-	-	-	-1.22685

10338961	-	-	-	-1.21753
10428509	239420	Csmd3	CUB and Sushi multiple domains 3	-1.21523
10501802	99887	Tmem56	transmembrane protein 56	-1.21032
10600901	11835	Ar	androgen receptor	-1.20799
10342082	-	-	-	-1.20771
10368289	18605	Enpp1	ectonucleotide pyrophosphatase/phosphodiesterase 1	-1.20168
10562731	-	-	-	-1.20163
10409833	14451	Gas1	growth arrest specific 1	-1.19062
10567355	64297	Gprc5b	G protein-coupled receptor, family C, group 5, member B	-1.18738
10341287	-	-	-	-1.18132
10340887	-	-	-	-1.18027
10343224	-	-	-	-1.18026
10573747	11513	Adcy7	adenylate cyclase 7	-1.18016
10338417	-	-	-	-1.17947
10338850	-	-	-	-1.17619
10523758	433926	Lrrc8b	leucine rich repeat containing 8 family, member B	-1.17196
10530998	243083	Tmprss11f	transmembrane protease, serine 11f	-1.17106
10344329	-	-	-	-1.16768
10340262	-	-	-	-1.15648
10421932	211712	Pcdh9	protocadherin 9	-1.15611
10341140	-	-	-	-1.15456
10338341	-	-	-	-1.1536
10502156	66815	Ccdc109b	coiled-coil domain containing 109B	-1.15281
10395596	-	-	-	-1.15039
10340099	-	-	-	-1.14906
10503448	17389	Mmp16	matrix metallopeptidase 16	-1.14517
10343995	-	-	-	-1.14476
10342174	-	-	-	-1.14112
10544438	-	-	-	-1.13725
10338489	-	-	-	-1.13659
10341902	-	-	-	-1.12949
10340949	-	-	-	-1.11953
10506470	242594	1700024P16Rik	RIKEN cDNA 1700024P16 gene	-1.11937
10479950	14007	Cugbp2	CUG triplet repeat, RNA binding protein 2	-1.11833
10344409	-	-	-	-1.1114
10341631	-	-	-	-1.1112
10380289	67468	Mmd	monocyte to macrophage differentiation-associated	-1.10997
10341521	-	-	-	-1.10947
10428453	239420	Csmd3	CUB and Sushi multiple domains 3	-1.10684
10339596	-	-	-	-1.10565
10523717	20750	Spp1	secreted phosphoprotein 1	-1.10511
10339129	-	-	-	-1.1001
10411459	380863	Tmem171	transmembrane protein 171	-1.09856
10344233	-	-	-	-1.09562
10366376	353025	Caps2	calcyphosphine 2	-1.09406

10340806	-	-	-	-1.09396
10341767	-	-	-	-1.09087
10417579	74430	4930452B06Rik	RIKEN cDNA 4930452B06 gene	-1.0906
10338379	-	-	-	-1.08517
10541075	20315	Cxcl12	chemokine (C-X-C motif) ligand 12	-1.08255
10581813	74568	Mlkl	mixed lineage kinase domain-like	-1.0804
10344299	-	-	-	-1.07695
10571907	71306	Mfap3l	microfibrillar-associated protein 3-like	-1.07638
10340021	-	-	-	-1.07622
10341751	-	-	-	-1.07392
10584259	235180	Fez1	fasciculation and elongation protein zeta 1 (zygin I)	-1.07282
10338302	-	-	-	-1.07026
10342570	-	-	-	-1.06931
10342173	-	-	-	-1.06838
10414269	12176	Bnip3	BCL2/adenovirus E1B interacting protein 3	-1.0667
10343269	-	-	-	-1.06486
10341457	-	-	-	-1.06269
10342291	-	-	-	-1.06097
10522368	70701	Nipal1	NIPA-like domain containing 1	-1.06063
10340128	-	-	-	-1.06045
10339087	-	-	-	-1.05301
10343059	-	-	-	-1.05231
10358434	18783	Pla2g4a	phospholipase A2, group IVA (cytosolic, calcium-dependent)	-1.05181
10339060	-	-	-	-1.04957
10375443	171285	Havcr2	hepatitis A virus cellular receptor 2	-1.04894
10340726	-	-	-	-1.04768
10518147	14726	Pdpn	podoplanin	-1.04456
10536505	17295	Met	met proto-oncogene	-1.04302
10344384	-	-	-	-1.04206
10427590	20512	Slc1a3	solute carrier family 1 (glial high affinity glutamate transporter), member 3	-1.04148
10340351	-	-	-	-1.03904
10472923	11639	Ak3l1	adenylate kinase 3-like 1	-1.03836
10550877	16534	Kcnn4	potassium intermediate/small conductance calcium-activated channel, subfamily N, member 4	-1.03769
10344552	-	-	-	-1.03702
10338787	-	-	-	-1.03424
10389214	20308	Ccl9	chemokine (C-C motif) ligand 9	-1.0338
10395457	14009	Etv1	ets variant gene 1	-1.03085
10344588	-	-	-	-1.03022
10501586	13609	S1pr1	sphingosine-1-phosphate receptor 1	-1.02963
10338538	-	-	-	-1.02909
10607888	333605	Frmpd4	FERM and PDZ domain containing 4	-1.0282
10339429	-	-	-	-1.02775
10340133	-	-	-	-1.02698
10504670	-	-	-	-1.02621

10355312	22779	Ikzf2	IKAROS family zinc finger 2	-1.02612
10342496	-	-	-	-1.0182
10421924	211712	Pcdh9	protocadherin 9	-1.01532
10511703	192656	Ripk2	receptor (TNFRSF)-interacting serine-threonine kinase 2	-1.01184
10344598	-	-	-	-1.01075
10341448	-	-	-	-1.01067
10341990	-	-	-	-1.00327

Table S2. Overlap of the lists of differentially expressed genes with the lists of various recently published stemness markers, with the genes whose TSS lies within a CpG island (CpG), with the databases of chromatin-related genes (ChromoDB) and with the databases of imprinted genes Geneimprint and Otago.

STEMNESS MARKERS

	Repressed	Induced
ES_exp1	n=3 (1.3) P=0.15	n= 11 (6) P=0.04
ES_exp2	n=0 (0.1) P=1	n=0 (0.7) P=1
Eed_targets	n= 9(3.6) P=0.0089	n= 55 (16.2) P=1.5e-15
H3K27_bound	n= 6 (3.8) P=0.19	n= 49 (17.5) P=7.6e-11
Myc_targets1	n= 3 (0.7) P=0.037	n= 6 (3.3) P=0.12
Myc_targets2	n=1 (2.5) P=0.92	n= 2 (11.4) P=1
NOS_TFs	n= 1 (0.1) P=0.12	n= 1 (0.6) P=0.45
NOS_targets	n= 3 (0.6) P=0.022	n= 6 (2.7) P=0.055
Nanog_targets	n= 5 (3.4) P=0.25	n= 26 (15.5) P=0.0072
Oct4_targets	n= 4 (1) P=0.017	n= 8 (4.5) P=0.081
PRC2_targets	n= 6 (2.3) P=0.026	n= 30 (10.3) P=1.9e-07
Sox2_targets	n=4 (2.5) P=0.24	n= 19 (11.4) P=0.022
Suz12_targets	n= 7 (3.6) P=0.069	n= 51 (16.4) P=5.8e-13

IMPRINTING

	Repressed	Induced
chromDB	n=0 (1.1) P=1	n= 1 (4.9) P=0.99
CpG	n= 30 (36.4) P=0.94	n= 185 (166) P=0.026
GENEIMPRINT	n=0 (0.2) P=1	n= 8 (1) P=7.2e-06
OTAGO	n= 1 (0.2) P=0.22	n= 9 (1.1) P=1.6e-06

Statistical analysis: n = number of found genes. In parenthesis number of expected by chance . P-values are indicated, significant P-values (P<0.02) are in bold. Links provide the corresponding lists of genes.

Table S3. Overlap of the lists of differentially expressed genes with the sarcoma signatures identified in Francis et al, 2007, BMC Genomics 8: 73 and in Baird et al, 2005.
Statistical analysis: n=number of found genes. In parenthesis number of expected by chance . P-values are indicated, significant P-values (P<0.02) are in bold. Links provide the corresponding lists of genes.

**BAIRD**				
	**repressed**		**induced**	
DFSP	n=3 (0.7)	P=0.032	n=13 (3.1)	**P=1.7e-05**
EWS	n=2 (0.8)	P=0.21	n=6 (3.8)	P=0.19
FIBRO	n=0 (0.3)	P=1	n=2 (1.5)	P=0.45
GIST	n=1 (0.9)	P=0.61	n=12 (4.3)	**P=0.0013**
HPC	n=0 (0.7)	P=1	n=7 (3.2)	P=0.043
LIPO	n=2 (0.6)	P=0.12	n=5 (2.8)	P=0.14
LMS	n=1 (0.7)	P=0.53	n=4 (3.4)	P=0.44
MFH	n=3 (1.1)	P=0.097	n=7 (5.0)	P=0.23
MPNST	n=0 (0.5)	P=1	n=1 (2.5)	P=0.92
MULL	n=2 (0.4)	P=0.065	n=3 (1.9)	P=0.29
OS	n=0 (0.4)	P=1	n=7 (1.9)	**P=0.0032**
RMS	n=1 (0.5)	P=0.42	n=11 (2.5)	**P=4e-05**
SS	n=0 (0.6)	P=1	n=12 (2.5)	**P=9.2e-06**

Abbreviations: DFSP = dermatofibrosarcoma; EWS = Ewing's sarcoma; FIBRO = fibrosarcoma; GIST = gastrointestinal stromal tumor; HPC = hemangiopericytoma; LMS = leiomyosarcoma; LIPO = liposarcoma; MFH = malignant fibrous histiocytoma; OS = osteosarcoma; MPNST = malignant peripheral nerve sheath tumor; RMS = rhabdomyosarcoma; SS = synovial sarcoma.

FRANCIS					
		**repressed**		**induced**	
DIPLIPO	**repressed**	n=0 (0.2)	P=1	n=0 (0.9)	P=1
	**induced**	n=0 (0.3)	P=1	n=4 (1.2)	P=0.036
FIBRO	**repressed**	n=1 (0.2)	P=0.16	n=3 (0.8)	P=0.043
	**induced**	n=0 (0.4)	P=1	n=12 (2.0)	**P=9.1e-07**
GIST	**repressed**	n=5 (2.7)	P=0.13	n=28 (12.3)	**P=5.2e-05**
	**induced**	n=2 (2.1)	P=0.62	n=17 (9.6)	**P=0.016**
LEIO	**repressed**	n=2 (0.7)	P=0.16	n=11 (3.3)	**P=0.00052**
	**induced**	n=3 (0.9)	P=0.055	n=4 (3.9)	P=0.55
MFHUPS	**repressed**	n=0 (0.7)	P=1	n=8 (3.4)	P=0.021
	**induced**	n=0 (0.8)	P=1	n=5 (3.8)	P=0.33
MPNST	**repressed**	n=3 (0.8)	P=0.041	n=4 (3.5)	P=0.46
	**induced**	n=0 (0.8)	P=1	n=10 (3.6)	**P=0.0038**
MYXLIPO	**repressed**	n=4 (1.7)	P=0.097	n=13 (7.9)	P=0.057
	**induced**	n=2 (1.4)	P=0.4	n=17 (6.2)	**P=0.0002**
SS	**repressed**	n=21 (6.7)	**P=1.8e-06**	n=48 (30.5)	**P=0.0012**
	**induced**	n=2 (5.6)	P=0.98	n=59 (25.3)	**P=9.8e-10**

Abbreviations: SS = synovial sarcoma. Dplipo=differentiated/pleomorphic liposarcoma. Fibro= fibrosarcoma. Gist= gastrointestinal stromal tumor. Leio= leiomyosarcoma. Mfhups=malignant fibrous histiocytomal undifferentiated pleomorphic sarcoma. Mpnst= malignant peripheral nerve sheat tumor. myxlipo= myxoid liposarcoma

Table S4. List of genes affected by SS18-SSX1 in C3H10T1/2 and included in the KEGG pathway term 04310 (Wnt signaling pathway), in the Wnt targets database or in the lists of genes affected by stimulation of C3H10T1/2 with recombinant WNT3a.

KEGG pathway term 04310 (Wnt signaling pathway)

Induced. Statistical analysis: 9 elements, expected 2.5, P = 0.00106899.

Axin2	axin2
Fzd1	frizzled homolog 1 (Drosophila)
Nfatc4	nuclear factor of activated T-cells, cytoplasmic, calcineurin-dependent 4
Nkd1	naked cuticle 1 homolog (Drosophila)
Nkd2	naked cuticle 2 homolog (Drosophila)
Sfrp2	secreted frizzled-related protein 2
Wif1	Wnt inhibitory factor 1
Wnt5a	wingless-related MMTV integration site 5A
Wnt6	wingless-related MMTV integration site 6

Wnt targets

Induced. Statistical anlysis: 17 elements, expected 1.6, P = 5.2e-13.

Axin2	axin2
Bmp4	bone morphogenetic protein 4
Cldn1	claudin 1
Edn1	endothelin 1
Egfr	epidermal growth factor receptor
Fgf18	fibroblast growth factor 18
Igf1	insulin-like growth factor 1
Igf2	insulin-like growth factor 2
Il6	interleukin 6
Irx3	Iroquois related homeobox 3 (Drosophila)
Islr	immunoglobulin superfamily containing leucine-rich repeat
Jag1	jagged 1
Lgr5	leucine rich repeat containing G protein coupled receptor 5
Rhou	ras homolog gene family, member U
Sfrp2	secreted frizzled-related protein 2
Stra6	stimulated by retinoic acid gene 6
Wisp2	WNT1 inducible signaling pathway protein 2

Genes affected by recombinant WNT3a in C3H10T1/2.

Induced. Statistical analysis: 26 elements, expected 1.94, P = 3.529E-22

Adm	adrenomedullin

Axin2	axin2
Ccdc80	coiled-coil domain containing 80
Col8a1	collagen, type VIII, alpha 1
Edn1	endothelin 1
Fjx1	four jointed box 1 (Drosophila)
Gpm6b	glycoprotein m6b
Il33	interleukin 33
Inhba	inhibin beta-A
Lce1h	late cornified envelope 1H
Lgr5	leucine rich repeat containing G protein coupled receptor. 5
Lif	leukemia inhibitory factor
Lmo7	LIM domain only 7
Myo10	myosin X
Ndnf	neuron-derived neurotrophic factor
Nfatc4	nuclear factor of activated T cells, cytoplasmic, calcineurin dependent 4
Nkd2	naked cuticle 2 homolog (Drosophila)
Nrp2	neuropilin 2
Prr5l	proline rich 5 like
Rhou	ras homolog gene family, member U
Sema3c	sema domain, immunoglobulin domain (Ig), short basic domain, secreted, (semaphorin) 3C
Spry1	sprouty homolog 1 (Drosophila)
Timp3	tissue inhibitor of metalloproteinase 3
Tnfsf15	tumor necrosis factor (ligand) superfamily, member 15
Trabd2b	TraB domain containing 2B
Wnt5a	wingless-related MMTV integration site 5A
**Repressed**. Statistical anlysis: 9 elements, expected 0.500, P = 1.769E-09	
Etv1	ets variant gene 1
Gprin3	GPRIN family member 3
Hgf	hepatocyte growth factor
Ifi202b	interferon activated gene 202B
Ripk2	receptor (TNFRSF)-interacting serine-threonine kinase 2
S1pr1	sphingosine-1-phosphate receptor 1
Spp1	secreted phosphoprotein 1
Sytl2	synaptotagmin-like 2
Vip	vasoactive intestinal polypeptide

Table S5. Lists of the probesets that are differentially expressed In C3H10T1/2 stimulated with WNT3a
or wehicle with FDR 10%

List of genes induced by WNT3a stimulation in C3H10T1/2

probeset	gene id	gene symbol	gene name	logFC
10576386	69581	Rhou	ras homolog gene family, member U	1.641027215
10404783	13614	Edn1	endothelin 1	3.483504073
10372503	14160	Lgr5	leucine rich repeat containing G protein coupled receptor 5	3.24276336
10382228	12006	Axin2	axin2	3.871944035
10523021	54403	Slc4a4	solute carrier family 4 (anion exchanger), member 4	1.499687698
10538802	68169	Ndnf	neuron-derived neurotrophic factor	3.407334502
10545707	11468	Actg2	actin, gamma 2, smooth muscle, enteric	1.280873088
10491721	24063	Spry1	sprouty homolog 1 (Drosophila)	0.895440822
10595836	319859	E030011O05Rik	RIKEN cDNA E030011O05 gene	1.005053862
10539907	24100	Tpra1	transmembrane protein, adipocyte asscociated 1	1.078873958
10467425	20411	Sorbs1	sorbin and SH3 domain containing 1	1.474897413
10501879	99526	Usp53	ubiquitin specific peptidase 53	1.108759513
10588283	270192	Rab6b	RAB6B, member RAS oncogene family	0.837243901
10453049	260409	Cdc42ep3	CDC42 effector protein (Rho GTPase binding) 3	1.204164512
10505489	18491	Pappa	pregnancy-associated plasma protein A	1.248437514
10423471	18163	Ctnnd2	catenin (cadherin associated protein), delta 2	0.75521302
10395320	22160	Twist1	twist basic helix-loop-helix transcription factor 1	1.060775979
10513722	326623	Tnfsf15	tumor necrosis factor (ligand) superfamily, member 15	0.917312502
10428912	399603	Fam84b	family with sequence similarity 84, member B	0.97566484
10405211	23882	Gadd45g	growth arrest and DNA-damage-inducible 45 gamma	1.857243988
10474700	21825	Thbs1	thrombospondin 1	0.865857609
10514338	723895	Mir31	microRNA 31	0.870405769
10590623	80901	Cxcr6	chemokine (C-X-C motif) receptor 6	4.106906529
10423293	17909	Myo10	myosin X	0.738755164
10366746	320398	Lrig3	leucine-rich repeats and immunoglobulin-like domains 3	1.073467045
10507101	666048	Trabd2b	TraB domain containing 2B	0.702206199
10440091	12837	Col8a1	collagen, type VIII, alpha 1	1.362755501
10365845	13998	Fgd6	FYVE, RhoGEF and PH domain containing 6	1.082642221
10485402	14221	Fjx1	four jointed box 1 (Drosophila)	1.73958185
10344263	#N/A	#N/A	#N/A	0.773869622
10505143	#N/A	#N/A	#N/A	0.65013357
10482500	74194	Rnd3	Rho family GTPase 3	0.860359345
10505120	242481	Palm2	paralemmin 2	0.598300133
10403743	16323	Inhba	inhibin beta-A	1.806638762
10472097	71409	Fmnl2	formin-like 2	1.202756285
10383684	16886	Limk2	LIM motif-containing protein kinase 2	0.900351349
10503695	12014	Bach2	BTB and CNC homology 2	0.6696736
10513884	21885	Tle1	transducin-like enhancer of split 1, homolog of Drosophila E(spl)	0.648070891

10483000	16420	Itgb6	integrin beta 6	1.060596108
10439710	208177	Phldb2	pleckstrin homology-like domain, family B, member 2	0.564281178
10340020	#N/A	#N/A	#N/A	0.771932598
10464391	13797	Emx2	empty spiracles homeobox 2	0.591930976
10356593	55927	Hes6	hairy and enhancer of split 6	0.584662269
10595831	245007	Zbtb38	zinc finger and BTB domain containing 38	0.962463101
10509790	212647	Aldh4a1	aldehyde dehydrogenase 4 family, member A1	0.858332876
10385776	21414	Tcf7	transcription factor 7, T cell specific	1.232161435
10519998	74511	Lrrc17	leucine rich repeat containing 17	1.239793014
10606654	331524	Xkrx	X Kell blood group precursor related X linked	1.073681241
10556297	11535	Adm	adrenomedullin	1.048774274
10502469	56376	Pdlim5	PDZ and LIM domain 5	0.555081614
10469404	12296	Cacnb2	calcium channel, voltage-dependent, beta 2 subunit	0.839127147
10435948	67896	Ccdc80	coiled-coil domain containing 80	1.791906592
10365482	21859	Timp3	tissue inhibitor of metalloproteinase 3	0.668664667
10416371	67168	Lpar6	lysophosphatidic acid receptor 6	0.704915884
10499932	67718	Lce1h	late cornified envelope 1H	1.510215917
10536494	12390	Cav2	caveolin 2	0.778948943
10444890	15937	Ier3	immediate early response 3	1.239659688
10603151	14758	Gpm6b	glycoprotein m6b	1.746606962
10507099	666048	Trabd2b	TraB domain containing 2B	0.711732624
10423548	15529	Sdc2	syndecan 2	0.783301512
10383853	75828	Hormad2	HORMA domain containing 2	0.485151649
10552380	243958	Siglecg	sialic acid binding Ig-like lectin G	0.522903983
10374464	114716	Spred2	sprouty-related, EVH1 domain containing 2	0.620126031
10362201	14219	Ctgf	connective tissue growth factor	1.183846489
10413482	22418	Wnt5a	wingless-related MMTV integration site 5A	0.802280903
10416800	380928	Lmo7	LIM domain only 7	1.132327932
10570236	17207	Mcf2l	mcf.2 transforming sequence-like	0.711443991
10365230	21665	Tdg	thymine DNA glycosylase	0.484349446
10431974	223864	Rapgef3	Rap guanine nucleotide exchange factor (GEF) 3	0.615806413
10393904	77583	Notum	notum pectinacetylesterase homolog (Drosophila)	1.084416872
10343823	#N/A	#N/A	#N/A	0.543471232
10415396	73181	Nfatc4	nuclear factor of activated T cells, cytoplasmic, calcineurin dependent 4	0.479474791
10394488	17182	Matn3	matrilin 3	0.653485502
10368883	21665	Tdg	thymine DNA glycosylase	0.48754414
10467068	208449	Sgms1	sphingomyelin synthase 1	1.106900018
10534927	231805	Pilra	paired immunoglobin-like type 2 receptor alpha	1.050969064
10580807	16582	Kifc3	kinesin family member C3	0.401536362
10489343	30963	Ptpla	protein tyrosine phosphatase-like (proline instead of catalytic arginine), member a	0.666305267
10607225	210297	Lrch2	leucine-rich repeats and calponin homology (CH) domain containing 2	1.068684412
10571312	319520	Dusp4	dual specificity phosphatase 4	1.76248882
10395328	217463	Snx13	sorting nexin 13	0.451023979
10462102	72351	Ptar1	protein prenyltransferase alpha subunit repeat containing 1	0.748049953
10346843	18187	Nrp2	neuropilin 2	0.741766494

10340228	#N/A	#N/A	#N/A	1.133415014
10454514	225341	Lims2	LIM and senescent cell antigen like domains 2	1.96864688
10340902	#N/A	#N/A	#N/A	0.633929527
10595840	235534	Acpl2	acid phosphatase-like 2	1.093167273
10355312	22779	Ikzf2	IKAROS family zinc finger 2	1.12257754
10514133	69863	Ttc39b	tetratricopeptide repeat domain 39B	0.652306631
10480121	66960	Fam188a	family with sequence similarity 188, member A	0.587318839
10472436	26877	B3galt1	UDP-Gal:betaGlcNAc beta 1,3-galactosyltransferase, polypeptide 1	0.806730248
10569646	12443	Ccnd1	cyclin D1	0.390173068
10579744	16795	Large	like-glycosyltransferase	0.573510038
10489299	#N/A	#N/A	#N/A	0.449187384
10519886	20348	Sema3c	sema domain, immunoglobulin domain (Ig), short basic domain, secreted, (semaphorin) 3C	1.98059327
10437151	16516	Kcnj15	potassium inwardly-rectifying channel, subfamily J, member 15	0.470663457
10521602	231207	Cpeb2	cytoplasmic polyadenylation element binding protein 2	0.638540996
10382341	20606	Sstr2	somatostatin receptor 2	0.702350037
10504234	22249	Unc13b	unc-13 homolog B (C. elegans)	0.474665843
10462442	77125	Il33	interleukin 33	2.217156284
10514128	69863	Ttc39b	tetratricopeptide repeat domain 39B	0.628266548
10430871	21665	Tdg	thymine DNA glycosylase	0.481487873
10365729	237459	Cdk17	cyclin-dependent kinase 17	0.505964246
10347792	#N/A	#N/A	#N/A	0.768853034
10400095	15982	Ifrd1	interferon-related developmental regulator 1	0.682085005
10397002	217692	Sipa1l1	signal-induced proliferation-associated 1 like 1	0.688066899
10464997	#N/A	#N/A	#N/A	0.420345812
10485378	72446	Prr5l	proline rich 5 like	1.419482127
10491279	18759	Prkci	protein kinase C, iota	0.519391924
10410547	72293	Nkd2	naked cuticle 2 homolog (Drosophila)	2.552372174
10583870	73230	Bmper	BMP-binding endothelial regulator	0.982955802
10428407	239408	Tmem74	transmembrane protein 74	1.541569072
10588263	24059	Slco2a1	solute carrier organic anion transporter family, member 2a1	0.613998748
10338444	#N/A	#N/A	#N/A	0.484678229
10390897	69664	Krtap1-5	keratin associated protein 1-5	0.708592102
10548105	12444	Ccnd2	cyclin D2	0.376320423
10406823	#N/A	#N/A	#N/A	1.964592978
10340282	#N/A	#N/A	#N/A	0.406941245
10373918	16878	Lif	leukemia inhibitory factor	1.135376503
10504294	100213	Rusc2	RUN and SH3 domain containing 2	0.948096467
10428918	320469	9930014A18Rik	RIKEN cDNA 9930014A18 gene	0.754593782
10455483	240255	Ythdc2	YTH domain containing 2	0.370942036
10542822	66532	Rep15	RAB15 effector protein	0.467981299
10350123	93689	Lmod1	leiomodin 1 (smooth muscle)	1.402734515
10515994	69780	Smap2	stromal membrane-associated GTPase-activating protein 2	0.407904498
10496715	229937	Znhit6	zinc finger, HIT type 6	0.686912628
10494655	242109	Zfp697	zinc finger protein 697	1.162987218

List of genes repressed by WNT3a stimulation in C3H10T1/2				
**probeset**	**gene id**	**gene symbol**	**gene name**	**logFC**
10501586	13609	S1pr1	sphingosine-1-phosphate receptor 1	-1.432545485
10499160	12479	Cd1d1	CD1d1 antigen	-1.227488614
10438769	12737	Cldn1	claudin 1	-2.609045004
10475866	12125	Bcl2l11	BCL2-like 11 (apoptosis facilitator)	-0.91591272
10501608	22329	Vcam1	vascular cell adhesion molecule 1	-0.83912278
10474419	107515	Lgr4	leucine-rich repeat-containing G protein-coupled receptor 4	-1.45617322
10359571	14261	Fmo1	flavin containing monooxygenase 1	-1.011628655
10506701	230587	Glis1	GLIS family zinc finger 1	-1.464867264
10400989	238271	Kcnh5	potassium voltage-gated channel, subfamily H (eag-related), member 5	-1.145414686
10381934	77097	Tanc2	tetratricopeptide repeat, ankyrin repeat and coiled-coil containing 2	-0.938476816
10341539	#N/A	#N/A	#N/A	-1.197057899
10558410	19267	Ptpre	protein tyrosine phosphatase, receptor type, E	-0.876068713
10363970	76877	Rab36	RAB36, member RAS oncogene family	-0.990833695
10517287	230815	Man1c1	mannosidase, alpha, class 1C, member 1	-0.710814207
10516484	14620	Gjb3	gap junction protein, beta 3	-0.944842654
10475643	14178	Fgf7	fibroblast growth factor 7	-1.346921682
10381809	16416	Itgb3	integrin beta 3	-0.921519708
10427816	68070	Pdzd2	PDZ domain containing 2	-1.294022431
10436945	53881	Slc5a3	solute carrier family 5 (inositol transporters), member 3	-1.03861787
10363224	12140	Fabp7	fatty acid binding protein 7, brain	-0.881341912
10496373	73284	Ddit4l	DNA-damage-inducible transcript 4-like	-0.647199401
10422962	68646	Nadk2	NAD kinase 2, mitochondrial	-1.724563431
10461408	74760	Rab3il1	RAB3A interacting protein (rabin3)-like 1	-0.927779544
10595081	26944	Tinag	tubulointerstitial nephritis antigen	-0.899901791
10464917	12794	Cnih2	cornichon homolog 2 (Drosophila)	-0.608190583
10343800	#N/A	#N/A	#N/A	-0.572317431
10436941	121022	Mrps6	mitochondrial ribosomal protein S6	-0.545371979
10342190	#N/A	#N/A	#N/A	-0.957694464
10428376	11600	Angpt1	angiopoietin 1	-2.236153488
10344136	#N/A	#N/A	#N/A	-1.134216516
10394555	105014	Rdh14	retinol dehydrogenase 14 (all-trans and 9-cis)	-0.585462163
10574027	17748	Mt1	metallothionein 1	-0.984846311
10406934	14009	Etv1	ets variant gene 1	-1.179455973
10473058	99031	Osbpl6	oxysterol binding protein-like 6	-0.6355796
10359861	66447	Mgst3	microsomal glutathione S-transferase 3	-1.16231913
10604528	171170	Mbnl3	muscleblind-like 3 (Drosophila)	-0.549411513
10543319	27999	Fam3c	family with sequence similarity 3, member C	-0.676474084
10519857	15234	Hgf	hepatocyte growth factor	-3.504004952
10472162	14571	Gpd2	glycerol phosphate dehydrogenase 2, mitochondrial	-0.515441543
10339037	#N/A	#N/A	#N/A	-0.658751947
10432032	22337	Vdr	vitamin D receptor	-1.145161218

10446763	77889	Lbh	limb-bud and heart	-1.240320279
10557326	16190	Il4ra	interleukin 4 receptor, alpha	-0.525200204
10381939	77097	Tanc2	tetratricopeptide repeat, ankyrin repeat and coiled-coil containing 2	-0.722901696
10357249	68428	Steap3	STEAP family member 3	-0.666789366
10523717	20750	Spp1	secreted phosphoprotein 1	-1.813372481
10586017	72565	Uaca	uveal autoantigen with coiled-coil domains and ankyrin repeats	-0.512625343
10492590	242083	Ppm1l	protein phosphatase 1 (formerly 2C)-like	-0.576823834
10574023	17750	Mt2	metallothionein 2	-1.535225726
10348354	394434	Ugt1a9	UDP glucuronosyltransferase 1 family, polypeptide A9	-1.049038235
10481592	13429	Dnm1	dynamin 1	-0.811608344
10357280	72999	Insig2	insulin induced gene 2	-0.667615729
10543959	19242	Ptn	pleiotrophin	-1.971669517
10595702	68861	1190002N15Rik	RIKEN cDNA 1190002N15 gene	-0.734591609
10604961	14396	Gabra3	gamma-aminobutyric acid (GABA) A receptor, subunit alpha 3	-0.55936884
10340630	#N/A	#N/A	#N/A	-0.472525782
10342116	#N/A	#N/A	#N/A	-0.659994066
10471424	98952	Fam102a	family with sequence similarity 102, member A	-0.619932375
10594802	235461	Fam63b	family with sequence similarity 63, member B	-0.719956526
10359181	30935	Tor3a	torsin family 3, member A	-0.537597873
10410115	66129	Aaed1	AhpC/TSA antioxidant enzyme domain containing 1	-0.545190244
10604620	78755	Fam122b	family with sequence similarity 122, member B	-1.554404439
10420877	71988	Esco2	establishment of cohesion 1 homolog 2 (S. cerevisiae)	-0.49850711
10345046	71877	Efhc1	EF-hand domain (C-terminal) containing 1	-0.610242441
10344390	#N/A	#N/A	#N/A	-0.829303474
10480699	83768	Dpp7	dipeptidylpeptidase 7	-0.465268653
10605303	69537	Dnase1l1	deoxyribonuclease 1-like 1	-0.724636292
10505623	52829	Lurap1l	leucine rich adaptor protein 1-like	-0.479795425
10474437	68201	Ccdc34	coiled-coil domain containing 34	-0.979109272
10601771	78248	Armcx1	armadillo repeat containing, X-linked 1	-0.703159788
10339478	#N/A	#N/A	#N/A	-0.818211451
10409541	#N/A	#N/A	#N/A	-0.505820641
10406663	11881	Arsb	arylsulfatase B	-0.514336241
10492699	229445	Ctso	cathepsin O	-0.976009712
10419034	70045	2610528A11Rik	RIKEN cDNA 2610528A11 gene	-0.821502601
10547217	78752	Csgalnact2	chondroitin sulfate N-acetylgalactosaminyltransferase 2	-0.460907831
10441902	64074	Smoc2	SPARC related modular calcium binding 2	-0.914137839
10573979	14681	Gnao1	guanine nucleotide binding protein, alpha O	-0.57046357
10360398	26388	Ifi202b	interferon activated gene 202B	-1.171843199
10480714	227620	Uap1l1	UDP-N-acteylglucosamine pyrophosphorylase 1-like 1	-0.726651112
10549276	79362	Bhlhe41	basic helix-loop-helix family, member e41	-1.002373115
10394534	23967	Osr1	odd-skipped related 1 (Drosophila)	-0.827524915
10422227	24064	Spry2	sprouty homolog 2 (Drosophila)	-0.575776742
10382300	26399	Map2k6	mitogen-activated protein kinase kinase 6	-1.463835383
10556528	18576	Pde3b	phosphodiesterase 3B, cGMP-inhibited	-0.878412051
10462922	74055	Plce1	phospholipase C, epsilon 1	-0.886234791
10581538	18104	Nqo1	NAD(P)H dehydrogenase, quinone 1	-0.864772661

10409261	68203	Diras2	DIRAS family, GTP-binding RAS-like 2	-1.351855835
10511703	192656	Ripk2	receptor (TNFRSF)-interacting serine-threonine kinase 2	-0.559645354
10400607	320772	Mdga2	MAM domain containing glycosylphosphatidylinositol anchor 2	-1.397032241
10423599	17181	Matn2	matrilin 2	-1.057287363
10605357	210710	Gab3	growth factor receptor bound protein 2-associated protein 3	-0.471316972
10473312	241520	Fam171b	family with sequence similarity 171, member B	-0.825332794
10452419	13640	Efna5	ephrin A5	-0.704618071
10442643	79059	Nme3	NME/NM23 nucleoside diphosphate kinase 3	-0.752753519
10497399	18583	Pde7a	phosphodiesterase 7A	-0.477900531
10375751	216725	Adamts2	a disintegrin-like and metallopeptidase (reprolysin type) with thrombospondin type 1 motif, 2	-0.553545382
10563191	68667	Trpm4	transient receptor potential cation channel, subfamily M, member 4	-0.790448689
10484283	18573	Pde1a	phosphodiesterase 1A, calmodulin-dependent	-0.788394677
10595148	14858	Gsta2	glutathione S-transferase, alpha 2 (Yc2)	-0.467678163
10549842	384763	Zfp667	zinc finger protein 667	-0.410201157
10346882	23792	Adam23	a disintegrin and metallopeptidase domain 23	-1.138730275
10474181	99382	Abtb2	ankyrin repeat and BTB (POZ) domain containing 2	-0.643293564
10359908	19736	Rgs4	regulator of G-protein signaling 4	-2.502309687
10429160	20442	St3gal1	ST3 beta-galactoside alpha-2,3-sialyltransferase 1	-0.991822593
10496338	19055	Ppp3ca	protein phosphatase 3, catalytic subunit, alpha isoform	-0.908012224
10355147	67099	Mettl21a	methyltransferase like 21A	-0.591425994
10470446	20181	Rxra	retinoid X receptor alpha	-0.404090624
10406672	11881	Arsb	arylsulfatase B	-0.442287294
10545065	243385	Gprin3	GPRIN family member 3	-2.78988839
10595560	76365	Tbx18	T-box18	-0.444388177
10344421	#N/A	#N/A	#N/A	-1.051811878
10360391	15950	Ifi203	interferon activated gene 203	-1.526187828
10369531	70423	Tspan15	tetraspanin 15	-0.531196986
10343210	#N/A	#N/A	#N/A	-0.732576481
10451481	210982	Gltscr1l	GLTSCR1-like	-0.38577015
10571362	621080	AI429214	expressed sequence AI429214	-0.48574057
10419566	11731	Ang2	angiogenin, ribonuclease A family, member 2	-0.538965562
10526943	80290	Gpr146	G protein-coupled receptor 146	-0.65743575
10523674	74167	Nudt9	nudix (nucleoside diphosphate linked moiety X)-type motif 9	-0.552703954
10341524	#N/A	#N/A	#N/A	-1.127247342
10554926	66365	Ccdc90b	coiled-coil domain containing 90B	-0.791386485
10339933	#N/A	#N/A	#N/A	-0.829777663
10454831	67869	Paip2	polyadenylate-binding protein-interacting protein 2	-0.420568493
10573115	330812	Rnf150	ring finger protein 150	-1.260240872
10427015	11479	Acvr1b	activin A receptor, type 1B	-0.409559084
10338249	#N/A	#N/A	#N/A	-0.445716871
10461251	68852	Lrrn4cl	LRRN4 C-terminal like	-0.606937258
10490872	71710	Lrrcc1	leucine rich repeat and coiled-coil domain containing 1	-1.202330905
10451123	63959	Slc29a1	solute carrier family 29 (nucleoside transporters), member 1	-0.513680747
10457357	75739	Mpp7	membrane protein, palmitoylated 7 (MAGUK p55 subfamily member 7)	-0.444268039
10499045	80890	Trim2	tripartite motif-containing 2	-0.469394108

10607499	18675	Phex	phosphate regulating gene with homologies to endopeptidases on the X chromosome (hypophosphatemia, vitamin D resistant rickets)	-1.327727836
10460157	12894	Cpt1a	carnitine palmitoyltransferase 1a, liver	-0.556798944
10422598	20363	Sepp1	selenoprotein P, plasma, 1	-1.611077988
10339475	#N/A	#N/A	#N/A	-0.401893307
10343129	#N/A	#N/A	#N/A	-0.673816199
10554863	83671	Sytl2	synaptotagmin-like 2	-1.02555281
10607486	211612	Ptchd1	patched domain containing 1	-0.705110082
10338195	#N/A	#N/A	#N/A	-0.563188236
10442904	72106	Jmjd8	jumonji domain containing 8	-0.365283378
10519607	652925	Tmem243	transmembrane protein 243, mitochondrial	-0.530149564
10551080	71722	Cic	capicua homolog (Drosophila)	-0.367104064
10500736	229658	Vangl1	vang-like 1 (van gogh, Drosophila)	-0.526265821
10544062	330286	D630045J12Rik	RIKEN cDNA D630045J12 gene	-0.388892616
10367582	22353	Vip	vasoactive intestinal polypeptide	-2.010511814
10415413	277154	Nynrin	NYN domain and retroviral integrase containing	-0.352025492
10443940	77652	Zfp955a	zinc finger protein 955A	-0.398141809

Table S6. Lists of the probesets that are differentially expressed In STO infected with SS18-SSX1 or an empty vector, with FDR 15% .

**List of genes induced by SYT-SSX1 expression in STO**				
**probeset**	**gene id**	**gene symbol**	**gene name**	**logFC**
H3011D02	22333	Vdac1	voltage-dependent anion channel 1	1.74412
H3126E02	22333	Vdac1	voltage-dependent anion channel 1	1.88818
H3081E04	233545	2210018M11Rik	RIKEN cDNA 2210018M11 gene	0.83796
H3020B01	#N/A	#N/A	#N/A	1.28382
H3106C06	14677	Gnai1	guanine nucleotide binding protein (G protein), alpha inhibiting 1	0.72684
H3080D12	22384	Eif4h	eukaryotic translation initiation factor 4H	0.78766
H3015B01	16600	Klf4	Kruppel-like factor 4 (gut)	0.6276
H3005A09	14629	Gclc	glutamate-cysteine ligase, catalytic subunit	0.7247
H3057D02	68097	Dynll2	dynein light chain LC8-type 2	1.16374
H3092D03	433864	Gm1040	gene model 1040, (NCBI)	0.6513
H3007A07	16956	Lpl	lipoprotein lipase	0.56444
H3126B11	331563	C130006E23	hypothetical protein C130006E23	0.88512
H3052B06	18669	Abcb1b	ATP-binding cassette, sub-family B (MDR/TAP), member 1B	0.63238
H3114D03	17158	Man2a1	mannosidase 2, alpha 1	0.65246
H3112D02	67458	Ergic1	endoplasmic reticulum-golgi intermediate compartment (ERGIC) 1	0.76596
H3104C02	56353	Rybp	RING1 and YY1 binding protein	0.64246
H3109E08	382985	Rrm2b	ribonucleotide reductase M2 B (TP53 inducible)	0.72178
H3098B01	#N/A	#N/A	#N/A	0.60822
H3011B06	233726	Ipo7	importin 7	0.90732
H3140C06	234736	Rfwd3	ring finger and WD repeat domain 3	0.539
H3026B04	231327	Ppat	phosphoribosyl pyrophosphate amidotransferase	0.5218
H3106H09	#N/A	#N/A	#N/A	0.57418
H3118C07	15510	Hspd1	heat shock protein 1 (chaperonin)	0.55606
H3129G09	22223	Uchl1	ubiquitin carboxy-terminal hydrolase L1	0.5236
H3096B01	#N/A	#N/A	#N/A	0.69562
H3007B10	13681	Eif4a1	eukaryotic translation initiation factor 4A1	0.65714
H3034E02	19139	Prps1	phosphoribosyl pyrophosphate synthetase 1	0.56328
H3037D03	74467	Pus10	pseudouridylate synthase 10	0.62172
H3083B05	14105	Fusip1	FUS interacting protein (serine-arginine rich) 1	0.46958
H3132F05	50887	Nsbp1	nucleosome binding protein 1	0.55454
H3040C04	67103	Ptgr1	prostaglandin reductase 1	0.54018
H3119C08	16562	Kif1c	kinesin family member 1C	0.48056
H3041A09	16648	Kpna3	karyopherin (importin) alpha 3	0.4892
H3151A04	16403	Itga6	integrin alpha 6	0.59874
H3106B12	434459	EG434459	predicted gene, EG434459	0.5105
H3124E04	20315	Cxcl12	chemokine (C-X-C motif) ligand 12	0.45446
H3099A04	494504	Apcdd1	adenomatosis polyposis coli down-regulated 1	0.61182

H3074D09	13495	Drg2	developmentally regulated GTP binding protein 2	0.48316
H3057D01	20684	Sp100	nuclear antigen Sp100	0.61928
H3029H08	52118	Pvr	poliovirus receptor	0.70678
H3016A09	118445	Klf16	Kruppel-like factor 16	0.50694
H3075H07	75221	Dpp3	dipeptidylpeptidase 3	0.62414
H3102E05	#N/A	#N/A	#N/A	0.5941
H3015E11	54132	Pdlim1	PDZ and LIM domain 1 (elfin)	0.49018
H3044B12	108705	Pttg1ip	pituitary tumor-transforming 1 interacting protein	0.5096
H3150G02	18141	Nup50	nucleoporin 50	0.48056
H3151G02	67811	Poldip2	polymerase (DNA-directed), delta interacting protein 2	0.53098
H3105E05	66645	Pspc1	paraspeckle protein 1	0.52016
H3146E06	84004	Mcam	melanoma cell adhesion molecule	0.54394
H3159E05	78929	Polr3h	polymerase (RNA) III (DNA directed) polypeptide H	0.4553
H3012G05	97820	4833439L19Rik	RIKEN cDNA 4833439L19 gene	0.47602
H3039G07	#N/A	#N/A	#N/A	0.47482
H3015B12	70601	Ecd	ecdysoneless homolog (Drosophila)	0.45654
H3084E10	53414	Bysl	bystin-like	0.49806
H3024F01	14725	Lrp2	low density lipoprotein receptor-related protein 2	0.50046
H3083C01	71819	Kif23	kinesin family member 23	0.47126
H3100G07	78394	Ddx52	DEAD (Asp-Glu-Ala-Asp) box polypeptide 52	0.42928
H3134A02	56047	Msln	mesothelin	0.48166
H3107A12	70465	Wdr77	WD repeat domain 77	0.46246
H3027D05	17069	Ly6e	lymphocyte antigen 6 complex, locus E	0.50858
H3017D12	70350	Basp1	brain abundant, membrane attached signal protein 1	0.4695
H3100C04	233726	Ipo7	importin 7	0.72446
H3085G03	13057	Cyba	cytochrome b-245, alpha polypeptide	0.50044
H3088D05	13518	Dst	dystonin	0.46774
H3007B09	13681	Eif4a1	eukaryotic translation initiation factor 4A1	0.69558
H3146F01	11848	Rhoa	ras homolog gene family, member A	0.51868
H3024G02	19139	Prps1	phosphoribosyl pyrophosphate synthetase 1	0.41252
H3055G09	235534	Acpl2	acid phosphatase-like 2	0.51062
H3021F06	319583	Lig4	ligase IV, DNA, ATP-dependent	0.45072
H3005D07	#N/A	#N/A	#N/A	0.58514
H3033G12	319638	Nt5dc1	5'-nucleotidase domain containing 1	0.4134
H3025E08	50497	Hspa14	heat shock protein 14	0.4197
H3113E07	66922	Rras2	related RAS viral (r-ras) oncogene homolog 2	0.47008
H3138F10	18201	Nsmaf	neutral sphingomyelinase (N-SMase) activation associated factor	0.42574
H3043E11	66926	Trmt6	tRNA methyltransferase 6 homolog (S. cerevisiae)	0.45806
H3015A04	16906	Lmnb1	lamin B1	0.54658
H3007B11	13681	Eif4a1	eukaryotic translation initiation factor 4A1	0.61338
H3131D05	53945	Slc40a1	solute carrier family 40 (iron-regulated transporter), member 1	0.44786
H3105E04	66645	Pspc1	paraspeckle protein 1	0.4469
H3002G11	14629	Gclc	glutamate-cysteine ligase, catalytic subunit	0.41144
H3023C10	19291	Purb	purine rich element binding protein B	0.40584
H3059G09	408028	9230115E21Rik	RIKEN cDNA 9230115E21 gene	0.87982

H3134E11	70693	Gpr125	G protein-coupled receptor 125	0.39938
H3052F08	75430	3200002M19Rik	RIKEN cDNA 3200002M19 gene	0.41928
H3026A01	56348	Hsd17b12	hydroxysteroid (17-beta) dehydrogenase 12	1.13186
H3053H10	239405	Rspo2	R-spondin 2 homolog (Xenopus laevis)	0.69374
H3035B12	433956	Heatr2	HEAT repeat containing 2	0.44374
H3128F08	68705	Gtf2f2	general transcription factor IIF, polypeptide 2	0.40918
H3128D11	432940	Fam105b	family with sequence similarity 105, member B	0.47188
H3040C06	20425	Shmt1	serine hydroxymethyltransferase 1 (soluble)	0.41944
H3029C03	27205	Podxl	podocalyxin-like	0.50756
H3092D07	70408	Polr3f	polymerase (RNA) III (DNA directed) polypeptide F	0.45456
H3006G07	77573	Vps33a	vacuolar protein sorting 33A (yeast)	0.40082
H3063D10	74185	Gbe1	glucan (1,4-alpha-), branching enzyme 1	0.44152
H3003E07	#N/A	#N/A	#N/A	0.38992
H3003D08	213539	Bag2	BCL2-associated athanogene 2	0.5305
H3017E11	50497	Hspa14	heat shock protein 14	0.41618
H3048E11	#N/A	#N/A	#N/A	0.47094
H3013C11	21454	Tcp1	t-complex protein 1	0.42758
H3111A05	26441	Psma4	proteasome (prosome, macropain) subunit, alpha type 4	0.40424
H3058G09	17436	Me1	malic enzyme 1, NADP(+)-dependent, cytosolic	0.49864
H3067F10	26909	Exo1	exonuclease 1	0.5662
H3158C06	72828	Ubash3b	ubiquitin associated and SH3 domain containing, B	0.42466
H3033A06	#N/A	#N/A	#N/A	0.40186
H3078C06	408028	9230115E21Rik	RIKEN cDNA 9230115E21 gene	1.24352
H3137F01	#N/A	#N/A	#N/A	0.39862
H3126F01	18845	Plxna2	plexin A2	0.3736
H3076B12	27096	Trappc3	trafficking protein particle complex 3	0.54578
H3011A06	67895	Ppa1	pyrophosphatase (inorganic) 1	0.47916
H3154H06	70546	Zdhhc2	zinc finger, DHHC domain containing 2	0.42692
H3132C12	218138	Gmds	GDP-mannose 4, 6-dehydratase	0.4066
H3026H03	17215	Mcm3	minichromosome maintenance deficient 3 (S. cerevisiae)	0.4456
H3044H09	233315	Mtmr10	myotubularin related protein 10	0.45404
H3148D06	16434	Itpa	inosine triphosphatase (nucleoside triphosphate pyrophosphatase)	0.4084
H3104F05	69408	Dnajc17	DnaJ (Hsp40) homolog, subfamily C, member 17	0.41732
H3128D08	70231	Gorasp2	golgi reassembly stacking protein 2	0.425
H3030G12	15260	Hira	histone cell cycle regulation defective homolog A (S. cerevisiae)	0.43562
H3015F01	66889	Rnf128	ring finger protein 128	0.43832
H3153H08	50786	Hs6st2	heparan sulfate 6-O-sulfotransferase 2	0.40064
H3031G04	56095	Ftsj3	FtsJ homolog 3 (E. coli)	0.3936
H3122H08	338523	Jhdm1d	jumonji C domain-containing histone demethylase 1 homolog D (S. cerevisiae)	0.51594
H3151A07	68966	Ngdn	neuroguidin, EIF4E binding protein	0.36264
H3134B08	17936	Nab1	Ngfi-A binding protein 1	0.42196
H3029D01	20535	Slc4a2	solute carrier family 4 (anion exchanger), member 2	0.41052
H3118F12	233103	4931406P16Rik	RIKEN cDNA 4931406P16 gene	0.37908
H3028F12	68729	Trim37	tripartite motif-containing 37	0.3695
H3079B08	320685	Dctd	dCMP deaminase	0.37788

H3135H04	22117	Tst	thiosulfate sulfurtransferase, mitochondrial	0.47184
H3149G12	57230	Sap30bp	SAP30 binding protein	0.36778
H3076F09	66713	Actr2	ARP2 actin-related protein 2 homolog (yeast)	0.42838
H3148C02	50786	Hs6st2	heparan sulfate 6-O-sulfotransferase 2	0.42522
H3099D06	14725	Lrp2	low density lipoprotein receptor-related protein 2	0.37032
H3080H01	22273	Uqcrc1	ubiquinol-cytochrome c reductase core protein 1	0.3787
H3153E05	26428	Orc4l	origin recognition complex, subunit 4-like (S. cerevisiae)	0.44034
H3110H02	#N/A	#N/A	#N/A	0.50354
H3133H12	233073	U2af1l4	U2 small nuclear RNA auxiliary factor 1-like 4	0.36158
H3049E07	67834	Idh3a	isocitrate dehydrogenase 3 (NAD+) alpha	0.44098
H3108H02	58194	Sh3kbp1	SH3-domain kinase binding protein 1	0.49758
H3010B05	13821	Epb4.1l1	erythrocyte protein band 4.1-like 1	0.37482
H3118A02	66131	Tipin	timeless interacting protein	0.3555
H3073E09	68083	Pak1ip1	PAK1 interacting protein 1	0.34898
H3031H04	16419	Itgb5	integrin beta 5	0.46424
H3024H04	207214	Larp4	La ribonucleoprotein domain family, member 4	0.34904
H3024D12	13723	Emb	embigin	0.41282
H3038B02	73945	Otud4	OTU domain containing 4	0.40466
H3009C05	56207	Uchl5	ubiquitin carboxyl-terminal esterase L5	0.38246
H3009E09	52033	Pbk	PDZ binding kinase	0.36682
H3153F08	107817	Jmjd6	jumonji domain containing 6	0.3932
H3079G03	69902	Mrto4	MRT4, mRNA turnover 4, homolog (S. cerevisiae)	0.34424
H3047B04	#N/A	#N/A	#N/A	0.41728
H3079A09	12428	Ccna2	cyclin A2	0.38502
H3127B05	269424	Phf17	PHD finger protein 17	0.43504
H3077F07	320806	Gfm2	G elongation factor, mitochondrial 2	0.448
H3152A07	233833	Tnrc6a	trinucleotide repeat containing 6a	0.3694
H3085E04	72201	Otud6b	OTU domain containing 6B	0.48422

List of genes repressed by SS18-SSX1 expression in STO				
**probeset**	**gene id**	**gene symbol**	**gene name**	**logFC**
H3109A04	72289	Malat1	metastasis associated lung adenocarcinoma transcript 1 (non-coding RNA)	-1.42866
H3129A01	11668	Aldh1a1	aldehyde dehydrogenase family 1, subfamily A1	-0.77854
H3139E04	12266	C3	complement component 3	-0.92912
H3005A04	14955	H19	H19 fetal liver mRNA	-1.07192
H3144B07	14955	H19	H19 fetal liver mRNA	-1.09972
H3133E08	19280	Ptprs	protein tyrosine phosphatase, receptor type, S	-0.75948
H3036D07	#N/A	#N/A	#N/A	-0.91702
H3106G12	105559	Mbnl2	muscleblind-like 2	-0.8899
H3026H06	22196	Ube2i	ubiquitin-conjugating enzyme E2I	-0.86934
H3003G06	#N/A	#N/A	#N/A	-0.8707
H3148D05	13179	Dcn	decorin	-1.13488
H3131G12	#N/A	#N/A	#N/A	-0.68114
H3052D04	#N/A	#N/A	#N/A	-1.17404
H3040A09	216441	Slc26a10	solute carrier family 26, member 10	-0.83512
H3055F08	21923	Tnc	tenascin C	-1.15208
H3047C02	210711	1110007A13Rik	RIKEN cDNA 1110007A13 gene	-1.21264
H3075F03	50908	C1s	complement component 1, s subcomponent	-0.80254
H3144B06	14955	H19	H19 fetal liver mRNA	-0.97444
H3009F01	81910	Rrbp1	ribosome binding protein 1	-0.84842
H3026H11	19155	Npepps	aminopeptidase puromycin sensitive	-0.65592
H3096D08	433762	LOC433762	hypothetical gene LOC433762	-0.64662
H3093A05	71175	Nipbl	Nipped-B homolog (Drosophila)	-0.70654
H3135A05	15364	Hmga2	high mobility group AT-hook 2	-0.75896
H3076G06	20847	Stat2	signal transducer and activator of transcription 2	-0.6854
H3079D11	20496	Slc12a2	solute carrier family 12, member 2	-0.74194
H3123G09	14955	H19	H19 fetal liver mRNA	-0.99302
H3139C10	#N/A	#N/A	#N/A	-1.09738
H3063C08	209387	AI451617	expressed sequence AI451617	-0.64124
H3156C02	18616	Peg3	paternally expressed 3	-0.9162
H3138F12	20971	Sdc4	syndecan 4	-0.65978
H3010H10	72289	Malat1	metastasis associated lung adenocarcinoma transcript 1 (non-coding RNA)	-1.24618
H3056B01	#N/A	#N/A	#N/A	-1.26848
H3153C03	19712	Rest	RE1-silencing transcription factor	-0.80806
H3026D04	12444	Ccnd2	cyclin D2	-0.58066
H3054D10	#N/A	#N/A	#N/A	-0.96254
H3145H12	20185	Ncor1	nuclear receptor co-repressor 1	-0.5769
H3151G01	81898	Sf3b1	splicing factor 3b, subunit 1	-0.88118
H3055D06	#N/A	#N/A	#N/A	-1.27962
H3054E03	#N/A	#N/A	#N/A	-1.1635
H3053F05	#N/A	#N/A	#N/A	-1.1896
H3134H12	#N/A	#N/A	#N/A	-0.64432
H3053F04	#N/A	#N/A	#N/A	-0.97326

H3027D08	71844	Nupl1	nucleoporin like 1	-0.58932
H3053H09	#N/A	#N/A	#N/A	-0.92874
H3097D08	433762	LOC433762	hypothetical gene LOC433762	-0.61486
H3116C04	20336	Exoc4	exocyst complex component 4	-0.5678
H3052B12	#N/A	#N/A	#N/A	-1.199
H3149H07	16391	Irf9	interferon regulatory factor 9	-0.66974
H3012B10	#N/A	#N/A	#N/A	-0.77512
H3027C09	77087	Ankrd11	ankyrin repeat domain 11	-0.63784
H3056F09	#N/A	#N/A	#N/A	-1.24248
H3061F08	74450	Pank2	pantothenate kinase 2 (Hallervorden-Spatz syndrome)	-0.54916
H3112E12	17118	Marcks	myristoylated alanine rich protein kinase C substrate	-0.64238
H3116F10	#N/A	#N/A	#N/A	-0.97118
H3016H10	20683	Sp1	trans-acting transcription factor 1	-0.79978
H3097A09	#N/A	#N/A	#N/A	-0.71636
H3009C07	15519	Hsp90aa1	heat shock protein 90, alpha (cytosolic), class A member 1	-0.62738
H3132A10	#N/A	#N/A	#N/A	-0.94702
H3156E08	76824	2410166I05Rik	RIKEN cDNA 2410166I05 gene	-0.67154
H3023A06	67039	Rbm25	RNA binding motif protein 25	-0.8308
H3150E08	399510	Map4k5	mitogen-activated protein kinase kinase kinase kinase 5	-0.77622
H3143C06	18764	Pkd2	polycystic kidney disease 2	-0.67012
H3148C04	100434	Slc44a1	solute carrier family 44, member 1	-0.5239
H3071G01	81702	Ankrd17	ankyrin repeat domain 17	-0.52878
H3109A10	56374	Tmem59	transmembrane protein 59	-0.54972
H3124H07	11819	Nr2f2	nuclear receptor subfamily 2, group F, member 2	-0.60778
H3156G08	#N/A	#N/A	#N/A	-0.62182
H3048F02	227331	Gigyf2	GRB10 interacting GYF protein 2	-0.73822
H3154H12	67869	Paip2	polyadenylate-binding protein-interacting protein 2	-0.75394
H3117F07	50791	Magi2	membrane associated guanylate kinase, WW and PDZ domain containing 2	-0.60912
H3069C05	109331	Rnf20	ring finger protein 20	-0.65232
H3157F03	67248	Rpl39	ribosomal protein L39	-0.65936
H3129B08	14955	H19	H19 fetal liver mRNA	-0.96694
H3104C04	#N/A	#N/A	#N/A	-0.55256
H3052H06	67916	Ppap2b	phosphatidic acid phosphatase type 2B	-0.66484
H3153G01	14132	Fcgrt	Fc receptor, IgG, alpha chain transporter	-0.61424
H3053A07	20238	Atxn1	ataxin 1	-0.53
H3111C03	#N/A	#N/A	#N/A	-0.50272
H3099G09	76524	Cln6	ceroid-lipofuscinosis, neuronal 6	-0.59234
H3051A01	#N/A	#N/A	#N/A	-1.19376
H3136A07	12843	Col1a2	collagen, type I, alpha 2	-0.61242
H3002D02	11461	Actb	actin, beta	-0.57722
H3088H06	218952	Fermt2	fermitin family homolog 2 (Drosophila)	-0.5481
H3118F04	67974	Ccny	cyclin Y	-0.81934
H3154B07	16432	Itm2b	integral membrane protein 2B	-0.51112
H3148C08	12388	Ctnnd1	catenin (cadherin associated protein), delta 1	-0.63048
H3042C07	233532	Rsf1	remodeling and spacing factor 1	-0.70672

H3116C02	17998	#N/A	#N/A	-0.4804
H3036B02	74383	Ubap2l	ubiquitin associated protein 2-like	-0.49888
H3110C09	18810	Plec1	plectin 1	-0.50198
H3034H03	66923	Pbrm1	polybromo 1	-0.7592
H3069D01	83946	Phip	pleckstrin homology domain interacting protein	-0.48882
H3022A11	109624	Cald1	caldesmon 1	-0.87976
H3097B10	69188	Mll5	myeloid/lymphoid or mixed-lineage leukemia 5	-0.63786
H3001D01	66691	Gapvd1	GTPase activating protein and VPS9 domains 1	-0.76548
H3087H03	66660	Sltm	SAFB-like, transcription modulator	-0.47934
H3138C11	11426	Macf1	microtubule-actin crosslinking factor 1	-0.50358
H3006G03	19155	Npepps	aminopeptidase puromycin sensitive	-0.5458
H3137A05	218543	Sfrs12	splicing factor, arginine/serine-rich 12	-0.58552
H3149A09	16004	Igf2r	insulin-like growth factor 2 receptor	-0.53858
H3066E04	228005	Ppig	peptidyl-prolyl isomerase G (cyclophilin G)	-0.59768
H3067F01	229700	Rbm15	RNA binding motif protein 15	-0.7538
H3004G02	66360	2310002J21Rik	RIKEN cDNA 2310002J21 gene	-0.53276
H3040E06	15364	Hmga2	high mobility group AT-hook 2	-0.58624
H3131B06	51886	Fubp1	far upstream element (FUSE) binding protein 1	-0.51312
H3062B09	213109	Phf3	PHD finger protein 3	-0.453
H3128A09	208146	Yeats2	YEATS domain containing 2	-0.61818
H3133B12	13179	Dcn	decorin	-0.66162
H3034H02	#N/A	#N/A	#N/A	-0.638
H3040G06	208643	Eif4g1	eukaryotic translation initiation factor 4, gamma 1	-0.62904
H3002D05	56490	Zbtb20	zinc finger and BTB domain containing 20	-0.78504
H3146F11	223649	Nrbp2	nuclear receptor binding protein 2	-0.5559
H3059G04	319604	Fam168a	family with sequence similarity 168, member A	-0.6015
H3038F02	17425	Foxk1	forkhead box K1	-0.47306
H3003G11	14042	Ext1	exostoses (multiple) 1	-0.59812
H3052A11	77652	Zfp422-rs1	zinc finger protein 422, related sequence 1	-0.6516
H3135A01	19055	Ppp3ca	protein phosphatase 3, catalytic subunit, alpha isoform	-0.46444
H3111D03	75605	Jarid1b	jumonji, AT rich interactive domain 1B (Rbp2 like)	-0.49458
H3154B05	15531	Ndst1	N-deacetylase/N-sulfotransferase (heparan glucosaminyl) 1	-0.72358
H3023F02	18720	Pip5k1a	phosphatidylinositol-4-phosphate 5-kinase, type 1 alpha	-0.60892
H3125C04	68799	Rgmb	RGM domain family, member B	-0.65886
H3024C11	15519	Hsp90aa1	heat shock protein 90, alpha (cytosolic), class A member 1	-0.50112
H3148G01	54169	Myst4	MYST histone acetyltransferase monocytic leukemia 4	-0.46926
H3118B11	75302	Asxl2	additional sex combs like 2 (Drosophila)	-0.42534
H3048D02	103583	Fbxw11	F-box and WD-40 domain protein 11	-0.45302
H3008B05	13138	Dag1	dystroglycan 1	-0.64182
H3148A03	28193	Reep3	receptor accessory protein 3	-0.49032
H3084G03	14281	Fos	FBJ osteosarcoma oncogene	-0.48142
H3050D08	75785	Klhl24	kelch-like 24 (Drosophila)	-0.47994
H3017H09	212391	Lcor	ligand dependent nuclear receptor corepressor	-0.6554
H3096H03	#N/A	#N/A	#N/A	-0.69146
H3081H01	241296	Lrrc8a	leucine rich repeat containing 8A	-0.58506

H3056E10	242509	Bnc2	basonuclin 2	-0.42762
H3001E08	#N/A	#N/A	#N/A	-0.57694
H3151H12	66168	Grina	glutamate receptor, ionotropic, N-methyl D-aspartate-associated protein 1 (glutamate binding)	-0.57266
H3040C01	231051	Mll3	myeloid/lymphoid or mixed-lineage leukemia 3	-0.58158
H3028D11	267019	Rps15a	ribosomal protein S15a	-0.45374
H3034C04	326622	Upf2	UPF2 regulator of nonsense transcripts homolog (yeast)	-0.5139
H3025C06	29808	Mga	MAX gene associated	-0.57288
H3103A07	12361	Cask	calcium/calmodulin-dependent serine protein kinase (MAGUK family)	-0.56908
H3016E10	69581	Rhou	ras homolog gene family, member U	-0.43082
H3095H09	320713	Mysm1	myb-like, SWIRM and MPN domains 1	-0.48058
H3036C11	192196	Luc7l2	LUC7-like 2 (S. cerevisiae)	-0.62632
H3146G02	12628	Cfh	complement component factor h	-0.49814
H3002F10	11820	App	amyloid beta (A4) precursor protein	-0.4782
H3035B06	20658	Son	Son DNA binding protein	-0.57292
H3041C04	75605	Jarid1b	jumonji, AT rich interactive domain 1B (Rbp2 like)	-0.4741
H3139F04	105522	Ankrd28	ankyrin repeat domain 28	-0.52222
H3020A08	53323	Ube2k	ubiquitin-conjugating enzyme E2K (UBC1 homolog, yeast)	-0.58836
H3125F12	13726	Emd	emerin	-0.47724
H3025D08	11569	Aebp2	AE binding protein 2	-0.41316
H3004D05	103080	Sept10	septin 10	-0.40456
H3119B01	#N/A	#N/A	#N/A	-0.66466
H3009F02	17999	Nedd4	neural precursor cell expressed, developmentally down-regulated 4	-0.50504
H3064G05	66970	Ssbp2	single-stranded DNA binding protein 2	-0.4384
H3007H04	230584	Yipf1	Yip1 domain family, member 1	-0.48326
H3085G06	219132	D14Ertd668e	DNA segment, Chr 14, ERATO Doi 668, expressed	-0.50506
H3157D02	16145	Igtp	interferon gamma induced GTPase	-0.44396
H3032D03	18595	Pdgfra	platelet derived growth factor receptor, alpha polypeptide	-0.47026
H3133F12	12444	Ccnd2	cyclin D2	-0.4247
H3122G01	20280	Scp2	sterol carrier protein 2, liver	-0.4365
H3121G09	13003	Vcan	versican	-0.42754
H3119G07	76357	Trmt5	TRM5 tRNA methyltransferase 5 homolog (S. cerevisiae)	-0.5032
H3055G07	21808	Tgfb2	transforming growth factor, beta 2	-0.6447
H3117B01	12444	Ccnd2	cyclin D2	-0.4287
H3062G04	229503	BC023814	cDNA sequence BC023814	-0.44534
H3095A12	#N/A	#N/A	#N/A	-0.43466
H3095F01	#N/A	#N/A	#N/A	-0.52986
H3012G06	67554	Slc25a30	solute carrier family 25, member 30	-0.47186
H3030C03	67500	Ccar1	cell division cycle and apoptosis regulator 1	-0.48424
H3024A09	#N/A	#N/A	#N/A	-0.40556
H3053F02	#N/A	#N/A	#N/A	-0.55268
H3069C09	19877	Rock1	Rho-associated coiled-coil containing protein kinase 1	-0.48204
H3044B05	20462	Sfrs10	splicing factor, arginine/serine-rich 10 (transformer 2 homolog, Drosophila)	-0.58348
H3049H10	232341	Wnk1	WNK lysine deficient protein kinase 1	-1.12172
H3042A04	#N/A	#N/A	#N/A	-0.39818

H3114B10	108927	Lhfp	lipoma HMGIC fusion partner	-0.52652
H3002G06	75956	Srrm2	serine/arginine repetitive matrix 2	-0.5679
H3091C01	16974	Lrp6	low density lipoprotein receptor-related protein 6	-0.49074
H3152D12	12825	Col3a1	collagen, type III, alpha 1	-0.57898
H3079A03	#N/A	#N/A	#N/A	-0.7356
H3158G04	20848	Stat3	signal transducer and activator of transcription 3	-0.6976
H3151C09	12520	Cd81	CD81 antigen	-0.4213
H3147B04	14183	Fgfr2	fibroblast growth factor receptor 2	-0.4664
H3033D07	13867	Erbb3	v-erb-b2 erythroblastic leukemia viral oncogene homolog 3 (avian)	-0.56918
H3039A03	11480	Acvr2a	activin receptor IIA	-0.43578
H3040A12	13018	Ctcf	CCCTC-binding factor	-0.6693
H3103D02	241846	Lsm14b	LSM14 homolog B (SCD6, S. cerevisiae)	-0.81898
H3137F02	76199	Med13l	mediator complex subunit 13-like	-0.5966
H3040E01	26965	Cul1	cullin 1	-0.47126
H3002F06	13669	Eif3a	eukaryotic translation initiation factor 3, subunit A	-1.09986
H3050D01	232217	4933427D06Rik	RIKEN cDNA 4933427D06 gene	-0.63044
H3143D11	18027	Nfia	nuclear factor I/A	-0.47372
H3141E01	382643	#N/A	#N/A	-0.41948
H3019H12	26903	Dysf	dysferlin	-0.48736
H3043F04	#N/A	#N/A	#N/A	-0.44918
H3125F02	12395	Runx1t1	runt-related transcription factor 1; translocated to, 1 (cyclin D-related)	-0.59098
H3011G10	77110	Gpbp1l1	GC-rich promoter binding protein 1-like 1	-0.3821
H3050E09	72972	Gcap14	granule cell antiserum positive 14	-0.54782
H3023B10	22589	Atrx	alpha thalassemia/mental retardation syndrome X-linked homolog (human)	-0.70464
H3074B10	66840	Wdr45l	Wdr45 like	-0.48138
H3139G02	98214	#N/A	#N/A	-0.45524
H3127A04	20742	Spnb2	spectrin beta 2	-0.47338
H3123G01	20088	Rps24	ribosomal protein S24	-0.4054
H3133B06	12444	Ccnd2	cyclin D2	-0.47192
H3114A04	68193	Rpl24	ribosomal protein L24	-0.43516
H3040A01	116848	Baz2a	bromodomain adjacent to zinc finger domain, 2A	-0.45854
H3039E07	#N/A	#N/A	#N/A	-0.594
H3009G11	70387	Ttc9c	tetratricopeptide repeat domain 9C	-0.44894
H3138B01	12038	Bche	butyrylcholinesterase	-0.69266
H3131B04	20280	Scp2	sterol carrier protein 2, liver	-0.41232
H3085D04	#N/A	#N/A	#N/A	-0.43906
H3041F11	239647	Fam113b	family with sequence similarity 113, member B	-0.47566
H3056F10	13858	Eps15	epidermal growth factor receptor pathway substrate 15	-0.37546
H3125A01	99543	Olfml3	olfactomedin-like 3	-0.42458
H3071F07	#N/A	#N/A	#N/A	-0.622
H3137F08	70579	Zc3h11a	zinc finger CCCH type containing 11A	-0.72602
H3143C11	72587	Pan3	PAN3 polyA specific ribonuclease subunit homolog (S. cerevisiae)	-0.42102
H3157D03	80859	Nfkbiz	nuclear factor of kappa light polypeptide gene enhancer in B-cells inhibitor, zeta	-0.59714
H3072G10	228005	Ppig	peptidyl-prolyl isomerase G (cyclophilin G)	-0.56218
H3055A03	57377	Gcs1	glucosidase 1	-0.39672

H3005F05	#N/A	#N/A	#N/A	-0.51508
H3153F11	66660	Sltm	SAFB-like, transcription modulator	-0.43878
H3095H01	214899	Jarid1a	jumonji, AT rich interactive domain 1A (Rbp2 like)	-0.81912
H3155H07	#N/A	#N/A	#N/A	-0.48794
H3122F12	20280	Scp2	sterol carrier protein 2, liver	-0.5396
H3044A06	76199	Med13l	mediator complex subunit 13-like	-0.4453
H3149A05	78908	Igsf3	immunoglobulin superfamily, member 3	-0.42464
H3108F11	12412	Cbx1	chromobox homolog 1 (Drosophila HP1 beta)	-0.39058
H3089G07	#N/A	#N/A	#N/A	-0.43706
H3120H02	399510	Map4k5	mitogen-activated protein kinase kinase kinase kinase 5	-0.5531
H3038H12	57261	Brd4	bromodomain containing 4	-0.43414
H3020H02	#N/A	#N/A	#N/A	-0.50488
H3006D04	12767	Cxcr4	chemokine (C-X-C motif) receptor 4	-0.55564
H3089C03	69202	Ptms	parathymosin	-0.49484
H3144H05	71724	Aox3	aldehyde oxidase 3	-0.4162
H3017H07	#N/A	#N/A	#N/A	-0.36564
H3039C02	26441	Psma4	proteasome (prosome, macropain) subunit, alpha type 4	-0.49798
H3044F11	17758	Mtap4	microtubule-associated protein 4	-0.42598
H3145D11	20088	Rps24	ribosomal protein S24	-0.4981
H3082D10	218952	Fermt2	fermitin family homolog 2 (Drosophila)	-0.39416
H3092H01	11964	Atp6v1a	ATPase, H+ transporting, lysosomal V1 subunit A	-0.37936
H3069F09	109910	Zfp91	zinc finger protein 91	-0.37344
H3052B11	18557	Pctk3	PCTAIRE-motif protein kinase 3	-0.62352
H3044E01	72425	2410042D21Rik	RIKEN cDNA 2410042D21 gene	-0.48346
H3070C02	237436	Gas2l3	growth arrest-specific 2 like 3	-0.50584
H3147A06	219024	Tmem55b	transmembrane protein 55b	-0.59738
H3035A11	56484	Foxo3	forkhead box O3	-0.43686
H3045H04	#N/A	#N/A	#N/A	-0.44634
H3099B10	72930	Ppp2r2b	protein phosphatase 2 (formerly 2A), regulatory subunit B (PR 52), beta isoform	-0.4136
H3016D08	12177	Bnip3l	BCL2/adenovirus E1B interacting protein 3-like	-0.39144
H3047D02	11657	Alb	albumin	-0.49404
H3090D10	78801	Ak7	adenylate kinase 7	-0.41964
H3124B09	11820	App	amyloid beta (A4) precursor protein	-0.40286
H3122E09	24086	Tlk2	tousled-like kinase 2 (Arabidopsis)	-0.51008
H3029H07	#N/A	#N/A	#N/A	-0.41874
H3094B06	20658	Son	Son DNA binding protein	-0.49176
H3079D12	18807	Pld3	phospholipase D family, member 3	-0.36686
H3095A11	16563	Kif2a	kinesin family member 2A	-0.55718
H3059B07	16451	Jak1	Janus kinase 1	-0.40752
H3158D11	17390	Mmp2	matrix metallopeptidase 2	-0.36918
H3111H03	18027	Nfia	nuclear factor I/A	-0.56842
H3090F12	#N/A	#N/A	#N/A	-0.46886
H3097H08	19646	Rbbp4	retinoblastoma binding protein 4	-0.64496
H3134D08	18293	Ogdh	oxoglutarate dehydrogenase (lipoamide)	-0.38926
H3135A11	57294	Rps27	ribosomal protein S27	-0.5731

H3018G12	218194	Phactr1	phosphatase and actin regulator 1	-0.53524
H3018H10	18483	Palm	paralemmin	-0.44516
H3055A12	18477	Prdx1	peroxiredoxin 1	-0.3571
H3136H11	399510	Map4k5	mitogen-activated protein kinase kinase kinase kinase 5	-0.38558
H3118C12	267019	Rps15a	ribosomal protein S15a	-0.40486
H3145D12	20088	Rps24	ribosomal protein S24	-0.4316
H3110H11	19055	Ppp3ca	protein phosphatase 3, catalytic subunit, alpha isoform	-0.41714
H3115G11	14026	Evl	Ena-vasodilator stimulated phosphoprotein	-0.4256
H3145E12	15587	Hyal2	hyaluronoglucosaminidase 2	-0.38328
H3141B09	652994	#N/A	#N/A	-0.39464
H3044D09	20249	Scd1	stearoyl-Coenzyme A desaturase 1	-0.44914
H3096B10	50905	Il17rb	interleukin 17 receptor B	-0.41034
H3025H11	245867	Pcmtd2	protein-L-isoaspartate (D-aspartate) O-methyltransferase domain containing 2	-0.36088
H3097A10	229675	Rsbn1	rosbin, round spermatid basic protein 1	-0.39998
H3155D02	66898	Baiap2l1	BAI1-associated protein 2-like 1	-0.3499
H3133H03	11907	Ate1	arginyltransferase 1	-0.41462
H3106B11	69270	Gins1	GINS complex subunit 1 (Psf1 homolog)	-0.37116
H3010E02	242960	Fbxl5	F-box and leucine-rich repeat protein 5	-0.44388
H3157C11	66552	2010106G01Rik	RIKEN cDNA 2010106G01 gene	-0.58414
H3106G02	#N/A	#N/A	#N/A	-0.58768
H3053G10	13030	Ctsb	cathepsin B	-0.39264
H3070G02	56224	Tspan5	tetraspanin 5	-0.39336

Table S7: list of primers

.

PRIMER NAME	5' TO 3' SEQUENCE
**CLONING**	
SS18-kzFw	5'ACGCGTCGACCCACCATGGGCGGCAACATGTGTG3'
SSXnoStopBamHI-R	5'CGGGATCCCCCTCGTCATCTTCCTCAGGGTCACTGATCTC3'
V5-Stop-EcoRI-R	5'GGAATTCTCAACCGGTACGCGTAGAATCGAGACCGAG3'
EcoRI-stop-SSX-HA-R	5'GGAATTCCTCAGCTTGCGTAGTCTGGGACGTCGTATGGGTATCCCTCGTCATCTTCCTCAGGGTCACTGAT3'
MluI-stopSSX1-R	5'CGACGCGTTTACTCGTCATCTTCCTCA3'
mLEF-1KzFw	5'GCCGCCACCATGCCCCAACTTTCCGGAGG3'
mLEF-1-HA-R	5'GGAATTCCTCAGCTTGCGTAGTCTGGGAGGTCGTATGGGTATCCGATGTAGGCAGCTGTCATT3'
**qPCR**	
SS18-SSX Fw	5'ATAGACCAACACAGCCTGGAC 3'
SS18-SSX R	5'CTTCTTGGGCATGATCTGG 3'
hSS18 Fw	5'TGGAGGATATAGACCAACACAGC 3'
hSS18 R	5'TGCTGGTAATTTCCATACTGTCC 3'
Mouse Axin2-Fw	5'CGAGTGTGAGATCCACGGAAA3'
Mouse Axin2-R	5'GACTGGGTCGCTTCTCTTGAA3'
Mouse Axin2-5'UTR-Fw	5'CAAAGGAGAGCTTTGCTGTAAAA3'
Mouse Axin2-5'UTR-R	5'GCGTCTCACTGAGTTTGGATT3'
Human Axin2-Fw	5'GCAGTGTGAAGGCCAATGG3'
Human Axin2-R	5'GCAGGCGGTGGGTTCTC3'
mouseLEF-1 Fw	5'CAGCAACCTTACCCTGAGGTCA3'
mouseLEF-1 R	5'CGGCTTTCTTTTTGGAAGTCG3'
HumanLEF-1 Fw	5'AAACAGGAACATCCCCACAC3'
HumanLEF-1 R	5'CAGAGGCTTCTTAATGTGAGGTC3'
Mouse β-cateninF	5'TGCAGATCTTGGACTGGACA3'
Mouse β-cateninR	5'AAGAACGGTAGCTGGGATCA'3'
Mouse TCF7L1-Fw	5'GCTCCCAGTATTACCCCTCA3'
Mouse TCF7L1-R	5'GGACCTTCTTCGGCTGAGTA3'
Mouse TCF7L2-Fw	5'AAATGGCCACTGCTTGATGT3'
Mouse TCF7L1-R	5'GCACCACCGGTACTTTGTTC3'
**CHROMATIN ACCESSIBILITY**	
mAxin2(-3122-3038)Fw	5'CTGGAGAACTTGAAACATGAAATC3'
mAxin2(-3122-3038)R	5'CTTCCTTGGCCCTCGTCT3' 3 MspI sites
mAxin2(-2939-2843)Fw	5'GAGCGCCTCTGTGATTGG3'
mAxin2(-2939-2843)R	5'TGCCAGGACCTTATCAAAGC3' 3 MspI sites
mAxin2(-1238-1090)Fw	5'CCACCACTTCGGGTTTGT3'
mAxin2(-1238-1090)R	5'GCCAGGGAAGGATCAAACTA3' 3 MspI sites

mAxin2(-947-856)Fw	5’TGGCTCTAGGAGGTGGACTG3’
mAxin2(-947-856)R	5’AACTTAGAGGCTTTGATCTTCAAACT3’ 1 MspI site
mAxin2(+262+333)Fw	5’ACCAGGATGGTGCATACCTC3’
mAxin2(+262+333)R	5’ACCAGAAGTCCAGCGTATTCC3’ 1 MspI site
mAxin2(+476+545)Fw	5’TGGGATCAAGAAGCAACAGA3’
mAxin2(+476+545)R	5’CATCACTGCCTGGATCTCG3’ no MspI sites
mGAPDH-Fw	5’ACCACCAACTGCTTAGCCCCCCTGGCCAA3’
mGAPDH-R	5’GGATGCAGGGATGATGTTCTGGGCAGCCCCAC3’ no MspI sites
**CHROMATIN IP**	
mAxin2pr -1731 Fw	5’GATACAGATGCATTATGGGAACAC3’
mAxin2pr -1731 R	5’GCCTCAAATTCCACAGCCTA3’
mAxin2pr -390 Fw	5’CTGGAGAACTTGAAACATGAAATC3’
mAxin2pr -390 R	5’CTTCCTTGGCCCTCGTCT3’
mAxin2pr -147 Fw	5’GAGCGCCTCTGTGATTGG3’
mAxin2pr -147 R	5’TGCCAGGACCTTATCAAAGC3’
mAxin2pr +182 Fw	5’TAGTAGAGGGGTGCGGATTG3’
mAxin2pr +182 R	5’TCCTGGAAGGGAGGTATGC3’
mAxin2pr +1554 Fw	5’CCACCACTTCGGGTTTGT3’
mAxin2pr +2377 R	5’GCCAGGGAAGGATCAAACTA3’
mAxin2pr +2377 Fw	5’ACCTCCCGTGTCACTGTTTC3’
mAxin2pr +1554 R	5’AGCTCCACACACACTTTTTCC3’
mAxin2pr +3054 Fw	5’ACCAGGATGGTGCATACCTC3’
mAxin2pr +3054 R	5’ACCAGAAGTCCAGCGTATCC3’
mAxin2pr +3268 Fw	5’TGGCATCAAGAAGCAACAGA3’
mAxin2pr +3268 R	5’CATCACTGCCTGGATCTCG3’
Chr6intergenicF	5’GGGCTGGTGGTGATGTCTAC3’
Chr6intergenicR	5’TCAAATGCAGAAGCAGCATAA3’
